# Comprehensive review of tujia “Lian” medicinal botanical drugs: traditional classification system, phytochemical, and pharmacological profile

**DOI:** 10.3389/fphar.2026.1747999

**Published:** 2026-02-18

**Authors:** Nan Kuang, Yu Mao, Muhammad Aamer, Piaopiao Jiang, Feibing Huang, Yupei Yang, Wenbing Sheng, Caiyun Peng, Wei Wang, Bin Li

**Affiliations:** 1 TCM and Ethnomedicine Innovation and Development International Laboratory, Innovative Materia Medica Research Institute, School of Pharmacy, Hunan University of Chinese Medicine, Changsha, China; 2 College of Traditional Chinese Medicine, Hunan Food and Drug Vocational College, Changsha, China; 3 H. E. J. Research Institute of Chemistry, International Center for Chemical and Biological Sciences, University of Karachi, Karachi, Pakistan

**Keywords:** “Lian” medicinal botanical drugs, pharmacology, phytochemistry, traditional classification, Tujia ethnomedicine

## Abstract

Tujia medicine categorizes drugs with similar effects into several major classes based on their ordinal numbers, primarily using the 36 and 72 ordinal number systems for organization. The drugs that mainly function to dispel wind and cold, promote blood circulation and disperse blood stasis, eliminate fire, dispel qi, relieve pain, and address dampness. Additionally, clearing the lymphatic system is collectively known as the “Seventy-Two Lian” method. This narrative review aims to provide a classification of “Seventy-Two Lian” and their attributions to alias, source, nature, flavor, and efficiency. It also summarizes the modern pharmacological effects of each species and its corresponding “Lian” drug. The goal is to provide a comprehensive overview of the current state of the Tujia “Lian” drugs and to promote further research and the use of these resources. The literature search for “Lian” drugs was conducted across various scientific databases, including SciFinder, Web of Science, Elsevier, PubMed, and CNKI, as well as ancient books and monographs. It collected the names, plant sources, and medicinal parts of “Lian” drugs from these sources, and identified the replaced Latin names in Chinese Plant Intelligence (https://www.iplant.cn). Relevant pharmacological studies were searched across various databases using Latin names and common names. The “Seventy-Two Lian” has a long history within Tujia ethnomedicine. Alongside its traditional uses, modern pharmacological effects have gained widespread attention. Recent studies have shown that “Lian” drugs generally exhibit a range of effects, including anti-inflammatory, analgesic, antibacterial, antioxidant, antitumor, antiviral, insecticidal, antidiabetic, neuroprotective, and hepatoprotective effects, as confirmed by *in vivo* and *in vitro* studies. Additionally, this review article addresses the limitations of current “Lian” drug research and other pharmacological aspects, as well as future opportunities for toxicological studies. Tujia ethnomedicines, as an essential part of traditional Chinese medicine (TCM), have developed a unique theoretical framework and drug classification approach through extensive medical practice. This study focuses on the characteristic “Lian” drugs of the Tujia ethnic group, reviewing their botanical origins, medicinal properties (nature, flavor, and meridian tropism), and traditional uses. The “Lian” drugs of the Tujia people exhibit significant efficacy in clearing heat and detoxifying the body, promoting blood circulation to remove stasis, dispelling wind and dampness, stopping bleeding, and promoting diuresis. By integrating modern phytochemical and pharmacological research, this study examines the active metabolites and biological activities of these medicinal botanical drugs, providing critical theoretical foundations and practical guidance for the Tujia ethnic medicines.

## Graphical Abstract

**Figure:**
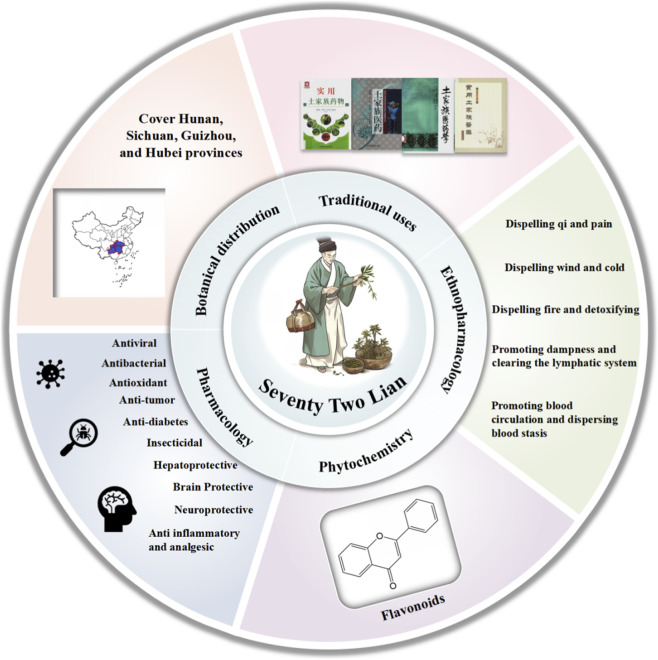

## 1 Introduction

The Tujia people have gained extensive knowledge of medicine and pharmaceuticals through their traditional practices, including tasting grass to identify medicinal botanical drugs and treat illnesses. This has led to the development of a unique medical system known as Tujia medicine. This system features a philosophical medical theory called the “Trialistic Theory,” as well as pharmacological theories such as the “Three-Nature” and “Eight-Flavors.” Additionally, the Tujia have accumulated pharmaceutical experience, folk remedies, and effective formulas through generations of practice (Zongguo, 2013). This wealth of knowledge is crucial for modernizing traditional medicine. The development of various Tujia botanical drugs has further demonstrated the clinical effectiveness of these therapies. However, due to the lack of a formal writing system, much of this valuable information has been passed down orally, resulting in a lack of standardized scientific documentation and organization (Huang et al., 2014).

A comprehensive literature search on Tujia “Lian” medicinal botanical drugs, traditional classification system, phytochemical, and pharmacological profile was conducted across various scientific databases, including SciFinder, Web of Science, Elsevier, PubMed, and CNKI, as well as ancient books and monographs. Search terms included the following keywords: “taxonomy,” “traditional uses,” “pharmacology,” “ethnomedicinal uses,” “medicinal botanical drugs,” “phytochemical metabolites,” “safety,” “efficacy,” and “toxicity,” in combination with the Tujia “Lian”.

### 1.1 Overview of the Tujia ethnic group

The Tujia ethnic group is mysterious. According to China’s seventh national census, the Tujia ethnic group has a population of about 8.0 million, ranking eighth among ethnic minorities. They are known as the “毕兹卡 bil jix kar” and have lived for generations in the Wuling area, where the provinces of Hubei, Hunan, Guizhou, and Chongqing intersect. Due to its subtropical location and complex terrain, the area is suitable for plants with diverse climatic needs. Moreover, most of the plants here are in mountainous regions, and most of them have not been destroyed. Some medicinal botanical drugs that have become extinct elsewhere can survive here. Therefore, this place is known as the “Central China Natural Medicine Warehouse”, with abundant resources of various animals, plants, and mineral medicinal botanical drugs (Xiong and Shen, 2012). Unfortunately, although the Tujia ethnic group has its own language, it lacks a written form; most of its traditional medicinal practices are passed down orally, with little standardized organization and documentation. It is mainly passed down through family inheritance, apprenticeship, oral transmission, and other methods, and most of them are relatively conservative and unwilling to pass it on lightly to others (Huang et al., 2014).

The basic characteristics of Tujia humanistic medicine distinguish it from traditional Chinese medicine and other local ethnic medicines, such as Miao and Dong medicine. It has its own fundamental medical theories, unique medical methods, abundant drug resources, and special application methods (Tian and Pan, 1994). However, living in a region with diverse ethnic groups, Tujia culture, medicine, and economy are constantly exchanging, and some are even integrating to varying degrees. There are similarities and differences between the 36 symptoms of sudden illness in Tujia and Miao medicine. Both use the 36 meridian symptoms and the 72 symptoms to describe them. Still, the names of the diseases are almost identical, while their clinical manifestations differ (Tian and Pan, 1994).

### 1.2 Historical origins and philosophical foundations

From the era of indigenous primitive tribes to the Qin and Han dynasties, Tujia medicine relied entirely on “oral transmission and inheritance from generation to generation,” and this was also a period of its formation. From the Tang and Song dynasties to the late Ming and early Qing dynasties, feudal dynasties gradually implemented the “tusi system” in ethnic minority areas, including those inhabited by the Tujia ethnic group. During the Kangxi reign of the Qing Dynasty, with the introduction of traditional Chinese medicine, Tujia medicine entered a relatively mature stage guided by the theory of sensory dialectics. After the founding of the People’s Republic of China, the Party and the government attached great importance to the development of traditional medicine. They not only included the development of traditional medicine in the Constitution, but also took many measures to gradually excavate and organize the scattered Tujia medicine among the people (Yang D. S. et al., 2016).

The origin and development of Tujia medicine can be traced back to “natural philosophy. Tujia medicine believes that “all things are born for me, all things are for my use, and all things are naturally capable of nurturing and treating people. There is no incurable disease in the world, only an incurable life.” At the same time, it is influenced by dialectical thinking. Tujia medicine uses a simple materialist thinking to understand and explore the physiology and pathology of human life. He also absorbed the essence of traditional medicine. He established the “Trialistic Theory” with the Taiji Yin and Yang (The Book of Changes), the mathematical philosophy of Heluo, and the Yin Yang and Five Elements Theory (Neijing) (Zhao, 2005).

### 1.3 The naming origins of “Lian” and “seventy-two, thirty-six.“

In early Tujia medicine, diseases were divided into Seventy-Two symptoms, Seventy-Two Winds, Seventy-Two Sha, Thirty-Six Tuberculosis, Thirty-Six Sores, Thirty-Six shock, and Thirty-Six injury diseases. Tujia medicine was also divided into Seventy-Two Shen, Seventy-Two Qi, Seventy-Two Lian, Thirty-Six Feng, Thirty-Six Huanyang, Thirty-Six Wugong, and Thirty-Six Xue. The Tujia people’s use of these numbers to name and classify diseases and medicines is not merely a simple mathematical description. It also has unique characteristics. Among them, “Seventy-Two” is derived from the Eight Trigrams of the Tai Chi Scripture of the “Shennong Yi” and the sixty-four hexagrams derived from it. Tujia medicine holds that it can account for the occurrence and development of all things in nature, including human life and diseases, as well as their auspiciousness and inauspiciousness. Originating from Qimen Dunjia, during the late Yin and early Zhou dynasties, Jiang Taigong and Lv Shang observed and calculated that there were three solar terms per section, one yuan per quarter, and twenty-four solar terms per year, seventy-two quarters. Tujia medicine believed in Jiang Taigong’s seventy-two divine calculation quarters. According to legend, the leader of the Tongtian Sect deployed the Thirty-Six Gang Formation, which was broken by Jiang Ziya, leading the Twenty-Eight Star Constellations and the Eight Marshals (Fang et al., 2007). Therefore, the Tujia Medical Thirty-Six were classified into some medicines and diseases. The Tujia medical values of thirty-six and seventy-two are regarded as the beliefs and value orientations of Tujia medical ethics, benevolence, and righteousness, and benevolent techniques.

In the Tujia language, “Lian” does not refer solely to lotus flowers, but is a general term for a specific type of plant that shares similar morphology or functions and is also related to its growth environment. It is usually an botanical drug aceous plant with leaves resembling lotus leaves, and it prefers moist conditions. Some have cleansing and detoxifying effects. These “Lian” drugs are primarily used to clear heat and detoxify, promote blood circulation, relieve pain, dispel dampness and reduce swelling, dispel wind and cold, among other uses. They are the core group of drugs used by the Tujia ethnic group to treat common conditions such as wind-dampness, snakebite, and inflammation. Knowledge of Tujia medicine is mostly passed down orally, heart to heart, and the “72 Lian,” as a medicinal experience, has been passed down from generation to generation, reflecting the profound understanding of the Tujia people towards local plant resources. These drug names are often combined with legends and folk customs (“Shenlian treats snake wounds”), becoming a part of national culture. Although the “Lian” of the Tujia ethnic group is mainly unrelated to lotus flowers, the term “Lian” may still borrow the symbolic meaning of “Lian” in Han culture, metaphorically referring to the “detoxification and purification” function of medicine. In some Tujia areas, influenced by Han culture, lotus flowers are also used in folk rituals. The naming of Tujia medicine combines form, efficacy, ecology, and culture, reflecting detailed observation of the natural world and embodying the simple pharmaceutical philosophy of “naming based on form and classifying based on efficacy.” This classification system crystallizes the wisdom of the Tujia ethnic group in adapting to the ecological environment of the Wuling mountainous area and in accumulating medical experience, with distinct ethnic and regional characteristics (Yuan, 2007).

## 2 The inheritance of quality standards for Tujia medicine

Quality standards are the bridge between traditional experience and modern science. In traditional Chinese medicine, they are the lifeline that ensures drug safety, effectiveness, and uniform, controllable quality.

### 2.1 The inheritance drawbacks of Tujia pharmacists

The Wuling mountainous region, which spans across Hunan, Sichuan, Guizhou, and Hubei provinces, is rich in medicinal resources (Fan et al., 2018). However, Tujia medical knowledge is mostly passed down orally, and errors can occur during its transmission and use. A common issue is the misnaming of medicinal plants, with many species being called by the same name or a single species having multiple names. For example, four different species of Tujia medicinal plants are known as “Guanyinlian,” and three species are called “Huoxuelian.” Additionally, plants from the same family and genus but different species are often mistaken for one another. For instance, “Dengtailian,” which originates from various plants within the Araceae family and genus, and “Tiexianlian,” derived from different plants in the Ranunculaceae family and *Clematis* genus, are listed separately in different Tujia Medical Classics (Zhao, 2005). Moreover, there are inconsistencies in the collected contents.

In Tujia ethnomedicine literature, specific drug names exhibit homophonic variation in written form despite identical pronunciation, primarily due to dialectal transliteration, historical changes, and regional differences. Examples include: “岩桥莲” (Yanqiaolian) *versus* “岩荞莲” (Yanqiaolian), “乱脚莲” (Luanjiaolian) *versus* “乱角莲” (Luanjiaolian), “碧血莲” (Bixuelian) *versus* “鼻血莲” (Bixuelian). This review compiles 96 “Lian” category medicinal materials from 46 plant families, with 101 botanical origins (Fang et al., 2007). For each entry, we record: standardized names and aliases, botanical origins, property and flavor profiles (e.g., “cold, bitter”), and therapeutic uses (Supplementary Table 1).

Due to their age, some plant names and Latin names have been revised and updated. Among these plants, only *Clematis chinensis*, from the Ranunculaceae family, has been included in the list of “Tiexianlian” drugs; other varieties, such as *Clematis peterae*, *Clematis lancifolia* var. *Ternata*, *C. peterae* var. *Trichocarpa*, *Clematis quinquefoliolata*, *Clematis kweichowensis*, and “Tiexianlian” from the Acanthaceae family, have not been included in Supplementary Table 1 and still require further verification and confirmation.

### 2.2 Quality control of Tujia medicine

The development of quality standards for Tujia ethnic medicine is at a critical stage, transitioning from local customs to national norms, and its system is multi-level but not yet fully unified. Due to the traditional Tujia medicine preaching method, which relies mainly on oral transmission, apprenticeship, and folk transmission, the phenomenon of “same name foreign object” and “same object but different names” is common. Owing to regional differences and oral transmission history, the same soil name may correspond to different plant families and genera, and the same medicinal botanical drug may have different Tujia-language names in other regions. Traditional quality evaluation primarily relies on sensory experience (seeing, touching, smelling, and tasting) and pharmacists’ practical experience, and lacks objective, quantitative indicators. At present, the primary basis is the inclusion of local standards. For example, the (Quality Standards for Traditional Chinese Medicine in Hubei Province) includes a large number of Tujia medicinal materials from Enshi and other places in Hubei Province, which is a pioneer and essential metabolite of Tujia medicine standardization; The (Hunan Province Traditional Chinese Medicine Standards) include the medicinal materials of the Tujia ethnic group in the Xiangxi region. The local standards of Chongqing city and Guizhou Province also include Tujia medicine in their respective jurisdictions. These local standards usually include: product name (Chinese name, Tujia language name, Latin scientific name), source (clarifying the family, genus, species, and medicinal parts), trait description (traditional experience identification), identification (microscopic identification, thin-layer chromatography identification), inspection (safety indicators such as moisture, ash content, impurities, heavy metals, and pesticide residues), extract, content determination, taste and functional indications based on Tujia medicine theory such as the “Three Elements” theory. There are also specialized books, such as “Tujia Medicinal Chronicles,” that systematically organize the origins and application experience of medicinal materials, serving as essential foundations for developing standards (Yuan, 2007). Although there is currently no fixed quality standard for Tujia medicine, given the current development trend, efforts are being made to translate Tujia medicine experience into modern scientific standards, using scientific language to safeguard ancient wisdom and enable it to safely and effectively benefit more people.

## 3 Modern pharmacological research on “Lian” drugs

**FIGURE 1:**
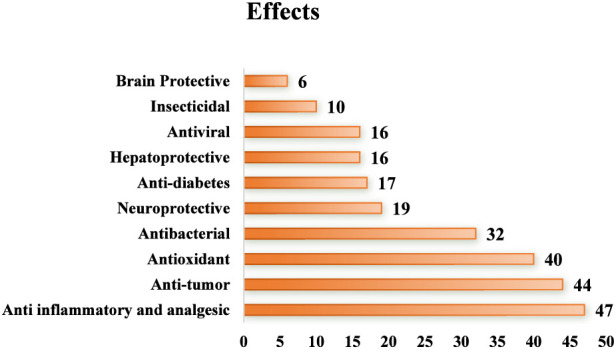
Number of “Lian” drugs for each modern pharmacological effect.

The 96 species of “Lian” drugs mentioned earlier belong to 46 families (Supplementary Table 1). The most common families include Ranunculaceae (10 species), Polygonaceae (7 species), and Lamiaceae (5 species), as shown in Figure 1. Most of these species are classified as having a cold or calming nature, with cold-natured botanical drugs accounting for 61%. Additionally, a significant portion of these drugs has a bitter taste, comprising 60% of the species.

**FIGURE 2:**
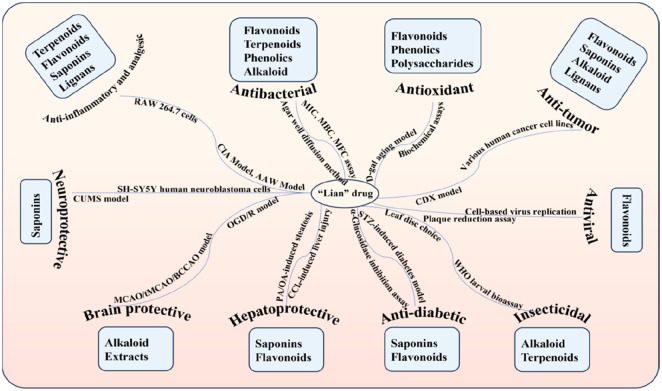
“Lian” medicinal botanical drugs and their phytochemical, and pharmacological profile.

A total of 17 toxic drugs have been documented, which generally show effects such as clearing heat and detoxifying the body, reducing swelling and nodules, cooling blood, relieving pain, and stopping dysentery. Some of these drugs have unique properties, flavors, and effects. Compared to traditional Chinese medicine (TCM), the “Lian” drugs used by the Tujia ethnic group closely resemble the “heat-clearing drugs” in TCM. Examples include *Scutellaria barbata* D. Don, *Lobelia chinensis* Lour., *Arisaema heterophyllum* Blume, and *Sauromatum giganteum* (Engl.) Cusimano & Hett. Furthermore, *Clematis chinensis* Osbeck, *Bistorta officinalis* Raf., *Sedum sarmentosum* Bunge, and *Stephania japonica* (Thunb.) are also classified as a heat-clearing drug in traditional Chinese medicine. Modern pharmacological studies suggest that as research into Tujia medicine advances, “Lian” drugs generally demonstrate anti-inflammatory, analgesic, antibacterial, antioxidant, and antitumor effects (Figure 2).

### 3.1 Anti-inflammatory and analgesic effects

The “Lian” drugs, known for their anti-inflammatory and analgesic effects, along with their corresponding bioactive fractions or metabolites, are shown in Supplementary Table 2. Terpenes are among the most prominent classes of anti-inflammatory metabolites, including neo-clerodane diterpenoids, hederagenin, gypenoside L, XLIX, LXXV, and cucurbitacin-type triterpenoids. Flavonoids are the most widely present anti-inflammatory metabolites and form the material basis for the activity of most plant extracts, including cynaroside, hyperin, scutellarein, luteolin, and quercetin. Anti-inflammatory efficacy is closely related to the number and positions of hydroxyl groups (-OH), especially the adjacent dihydroxy group on the B ring and the 4-carbonyl group in the C ring. Quercetin, one of the most well-known flavonoids, is almost ubiquitous and a potent antioxidant and anti-inflammatory agent. Saponins have surface activity, and many have anti-inflammatory and immunomodulatory effects. Among them, Dioscin and Deltonin are derived from Dioscorea plants and are steroidal saponins with anti-inflammatory and anti-tumor activities. Aescin/escin is a classic anti-inflammatory drug metabolite; lignin metabolites are derived from phenylpropanoid units and often exhibit anti-inflammatory, antioxidant, and hepatoprotective activities. Among them, Podophyllotoxin, a famous anti-tumor lignan, also has anti-inflammatory activity. The vast majority of active metabolites have been reported to inhibit the two central inflammatory signaling pathways, NF-κB and MAPK. This underpins the “broad-spectrum” anti-inflammatory effect (reducing the expression of TNF-α, IL-6, IL-1β, iNOS, and COX-2). Most studies follow the complete logical chain of “network pharmacology prediction, *in vitro* cell validation (RAW 264.7/BV-2), and *in vivo* animal models (LPS, DSS, CIA, MCAO, etc.)”, with a transparent chain of evidence. However, this also highlights the shortcomings of its single, mechanized *in vitro* model. The RAW 264.7 mouse macrophage line has been overused, and its response to LPS may not fully represent the complex behavior of human primary macrophages or specific tissue macrophages. Many studies rely solely on NO inhibition as the primary indicator, neglecting the assessment of other critical inflammatory mediators and cellular functions. Acute models dominate, while chronic and low-grade inflammation models (aging models and high-fat diet-induced metabolic inflammation) are poorly studied, with the latter being closer to the pathological state of most human chronic diseases. A large amount of literature remains at the stage of isolating new metabolites and testing their activity at a single high concentration (10–40 *µ*M). There is a Lack of systematic structural optimization and structure-activity relationship research on metabolites. Although target hypotheses have been proposed through molecular docking and inhibitor experiments, research directly demonstrating the eutectic structure, binding constants, or direct intracellular interactions between metabolites and hypothesized target proteins is rare. Network pharmacology predictions often provide dozens of potential targets, lacking experimental focus. This study believes that more human primary immune cells, organoids, and organ chip models should be used to validate experimental results and better simulate the human microenvironment. Their development and application should also be closer to complex animal models of human diseases. Directly identify and validate biological targets using chemical biology tools, such as active-molecule probes and photoaffinity labels, without relying on correlation-based speculation, and establish a standardized process for screening and evaluating the anti-inflammatory activity of natural products, including a unified positive control, cell model, and detection indicators. It is mandatory to complete pharmacokinetic and safety evaluations of GLP-compliant systems in the preclinical stage, laying a solid foundation for clinical trials.

### 3.2 Antibacterial effects

The “Lian” drugs, known for their antibacterial properties, are also associated with active fractions or metabolites (Table 1). Earlier research mainly focused on flavonoids, saponins, and alkaloids, as well as solvent fractions separated by polarity (ethyl acetate and *n*-butanol). These studies correctly indicated that antibacterial activity was enriched in specific polarity ranges, and as research progressed, characteristic metabolite categories were isolated and identified. Flavonoids (quercetin, quercitrin, afzelin, scutellarein, and flavonoid glycosides) exhibit antibacterial activity, often *via* membrane interference and enzyme inhibition. The glycosylation type and hydroxylation pattern significantly affect their activity and selectivity (making them more effective against Gram-positive bacteria). Terpenes and their derivatives, including triterpenoid saponins, volatile oils, hyperforin, and anemin, have hydrophobic glycosides and hydrophilic sugar chains that together determine their surfactant properties, leading to membrane perforation and content leakage, with outstanding effects on Gram-positive bacteria and fungi. Phenolic acids and polyphenolic metabolites (phenolic acids, phenolic metabolites, polyphenols, ellagic acid, ellagitannins) show activity related to protein precipitation, metal ion chelation, and enzyme inhibition. Alkaloids, such as lappaconitine and benzilisoquinoline alkaloids, have diverse antibacterial mechanisms, often involving interference with nucleic acid or protein synthesis. The study not only reports MIC values but also uses techniques such as molecular docking, enzyme inhibition, and gene knockout/knockdown to link specific chemical structures to key bacterial targets directly. The agar diffusion method (inhibition zone) and the broth dilution method (MIC/MBC) are widely accepted mainstream methods for antibacterial models. However, over 90% of the research remains in static *in vitro* models. The lack of dynamic PK/PD models, validation of *in vivo* infection models, and consideration of the host immune environment results in model singularity and clinical disconnection. The definition of antibacterial activity is too broad, and many studies confuse antibacterial and antifungal activities. However, the mechanisms and targets of the two differ significantly, requiring more precise classification of mechanisms. The future research paradigm must shift from a single *in vitro* activity report to an integrated study centered on depth of mechanistic understanding, *in vivo* validation, drug efficacy evaluation, and clinical needs.

**TABLE 1:** “Lian” drugs and their antibacterial effects.

Sr No.	“Lian” drugs name	Source	Study	Model/Assay	Conc./Dose range	Effective sites or metabolites
1	Chuanxinlian	*Aconitum sinomontanum* Nakai	*In vitro*	MIC assay, Agar well diffusion method	15.60–250 *μ*g/mL	Lappaconitine (Ahmad et al., 2008); Diterpenoid alkaloids (Yuan and Wang, 2012)
2	Xinanyinhualian	*Anemone davidii* Franch	*In vitro*	Agar well diffusion method, MIC assay	8–16 *μ*g/mL	Triterpenoid saponin (Yu et al., 2019)
3	Guanyinlian	*Angiopteris evecta* (G. Forst.) Hoffm	*In vitro*	Agar well diffusion method, MIC assay	62.5–250 *μ*g/mL, 200 *µ*g/well (12–20 mm)	Methanol extracts (Khan and Omoloso, 2008)
4	Dengtailian	*Arisaema heterophyllum* Blume*, Arisaema erubescens* (Wall.) Schott and *Arisaema amurense* Maxim	*In vitro*	MIC assay, Agar well diffusion method, Mycelial Growth Inhibition Assay, Spore germination assay	3.12–50 *μ*g/mL, 1.50–12.50 mg/mL	Anthraquinone, Diphenyl ether derivatives (Wang et al., 2012); Ethyl acetate extract (Lianrui and Mingsan, 2023)
5	Baierlian	*Asparagus cochinchinensis* (Lour.) Merr	*In vitro*	MIC assay, Agar well diffusion method	1.56–12.50 mg/mL	Ethyl acetate fraction (Fang et al., 2012)
6	Baierlian	*Asparagus densiflorus* (Kunth) Jessop	*In vitro*	MIC assay, MBC assay, Agar well diffusion method, Time-kill assay	0.78–12.50 mg/mL	Aqueous-ethanol extract (Mady et al., 2024)
7	Guanyinlian	*Balanophora involucrata* Hook. f	*In vitro*	MIC assay	16–128 *μ*g/mL	Phenolic acids (Wei et al., 2017)
8	Dahanliancao	*Bidens tripartita* L	*In vitro/In vivo*	MIC assay, MFC assay, *Ex vivo* porcine skin permeation and retention study, Agar well diffusion method	0.125–1.0 *μ*L/mL, 5 *µ*L/disc (15–22 mm), 0.5–2.0 mg/mL	Essential oil (Tomczykowa et al., 2018); Extracts, Essential oil (Tomczykowa et al., 2008)
9	Huoxuelian	*Bistorta officinalis* Raf	*In vitro*	MIC assay, Agar well diffusion method, HR-GIP, MBC assay, Anti-biofilm activity assay, Cytotoxicity assay	1.56–250 *μ*g/mL, 50–100 mg/mL, (11.5–18.5 mm)	Volatile oil (Cecotti et al., 2012); Ellagitannins, Agrimoniin (Pawłowska et al., 2020); Quercetin, Quercitrin, Afzelin (Liu Y. et al., 2014); ethanol extract, Ellagic acid, Total flavonoids (Yulan et al., 2025)
10	Tiexianlian	*Clematis chinensis* Osbeck	*In vitro*	MIC assay	1–40 *μ*g/mL	Anemonin and protoanemonin (Lin et al., 2021)
11	Babaolian	*Clerodendrum bungei* Steud	*In vitro/In vivo*	Loach (*Misgurnus anguillicaudatus*) infection model with *Aeromonas hydrophila,* Agar Dilution Method, Kirby-Bauer test, MIC assay, MBC assay, Agar well diffusion method	1–25 mg/mL, 50–200 mg/kg	*n*-Butanol extract (Li et al., 2025); Ethyl acetate fraction (Yin et al., 2008); Ethyl acetate extract, ethanol extract, *n*-butanol extract (He Z. et al., 2022)
12	Guanyinlian	*Dioscorea zingiberensis* C. H. Wright	*In vitro*	MIC assay, Time-kill assay, TEM, SYTOX Green assay, Membrane depolarization assay	0.5–2.0 *μ*g/mL	Diphenyl ether derivatives (Gu et al., 2023)
13	Bajiaolian	*Dysosma versipellis* (Hance) M. Cheng	*In vitro*	MIC assay, Growth curve analysis, GTPase activity assay, Polymerization inhibition assay, Molecular docking and dynamics simulation, FM, Agar well diffusion method	12.7 *µ*M, 6.25–100 *μ*g/mL	Epipodophyllotoxin derivative B2 (Song et al., 2022); Endophytic fungi (Tan et al., 2018)
14	Mohanlian	*Eclipta prostrata* (L.) L	*In vitro*	Agar well diffusion method, Agar Dilution Method, MIC assay, Kirby-Bauer test	12.5–500 *μ*g/mL, 50 *µ*g/disc (14.2–24.3 mm), 1.25–5.0 mg/mL	Ether extract (Feng et al., 2019); Flavonoid, Phenolic, Alkaloid (Timalsina and Devkota, 2021)
15	Honghanlian	*Hypericum ascyron* L	*In vitro*	MIC assay, MBC assay, Time-kill assay, DCFH-DA assay, Flow Cytometry, TUNEL assay, TEM, Caspase-like activity assay	1.56–128 *μ*g/mL	Phenolic metabolites (Li et al., 2015b); Ethyl acetate fraction (Li et al., 2015a)
16	Duiyuelian	*Hypericum sampsonii* Hance	*In vitro*	MIC assay, Agar well diffusion method	0.1–16 *μ*g/mL	Hyperforin, Hypericin, Flavonoids, 7-Epiclusianone, Sampsone A, and Hypericumxanthone A (Sun Z. et al., 2023)
17	Banbianlian	*Lobelia chinensis* Lour	*In vitro*	MIC assay, MABA, LORA, Cytotoxicity Assay	15.63–31.25 *μ*g/mL	N-hexane extract (Choi and Lee, 2016)
18	Qikonglian	*Osbeckia stellata* Buch. Ham. ex D. Don	*In vitro*	Agar well diffusion method, MIC assay, MFC assay	0.25–2.0 mg/mL	Extracts (Darmwal et al., 2025)
19	Qiyelian	*Paris polyphylla* var. *Chinensis* (Franch.) Hara	*In vitro*	MIC assay, MBC assay, Agar well diffusion method	1–50 *μ*g/mL	Polyphyllin D, Steroidal saponins, Ophiopogonin C' (Dantong et al., 2022)
20	Jiujielian	*Peristrophe japonica* (Thunb.) Bremek	*In vitro*	Agar well diffusion method, MIC assay	1.25–25 mg/mL, 20–100 mg/mL (11.2–15.8 mm)	Total flavonoids (Li-juan et al., 2017); ethanol extract (Qin and Luo, 2006)
21	Yanqiaolian	*Persicaria capitata* (Buch. Ham. ex D. Don) H. Gross	*In vitro/In vivo*	Kirby-Bauer test, MIC assay, MBC assay, *Ex vivo* adhesion assay, Time-kill assay, Agar well diffusion method	32–128 *μ*g/mL, 0.195–0.391 mg/mL, 400 mg/kg/day	Ethyl acetate fraction, Quercitrin (Liao et al., 2011); Aqueous extracts (Zhou et al., 2025); Ethyl acetate fraction, Phenolic acids, Flavonoids (YUlan et al., 2021)
22	Qiaokelian	*Persicaria chinensis* (L.) H. Gross	*In vitro*	MIC assay, MBC assay, Crystal violet biofilm assay, CLSM, Time-kill assay, qRT-PCR, Agar well diffusion method	0.78–125 mg/mL	Aqueous extract (Zeng et al., 2022); Water decoction, Total flavonoids (Fengfeng and Hua, 2025)
23	Guanyinzuolian	*Phedimus aizoon* (L.) Hart	*In vitro/In vivo*	Strawberry fruit bioassay against gray mold, Agar well diffusion method, MIC assay, MBC assay, Agar Dilution Method, Tomato fruit bioassay for gray mold control	0.15–8.0 mg/mL	Flavonoids (Ge et al., 2024); Polyphenols, Flavonoids (Lee et al., 2024); Flavonoid glycosides (Wang K. et al., 2022)
24	Dujiaolian	*Pinellia pedatisecta* Schott	*In vitro*	Agar well diffusion method, MIC assay	125–500 *μ*L/mL	Endophytic fungi (Kong et al., 2023)
25	Xionghuanglian	*Pleuropterus ciliinervis* Nakai	*In vitro*	MIC assay, Target Enzyme Inhibition Model	12.5 *µ*M, 3.13–6.25 *μ*g/mL	2′-Benzoyloxycinnamaldehyde (Kang et al., 2007)
26	Shuifulian	*Pontederia crassipes* Mart	*In vitro*	MIC and MBC assay	1–40 *μ*g/mL	Aqueous extracts (de Oliveira Silva et al., 2025)
27	Runxuelian	*Pyrola calliantha* Andres	*In vitro*	Agar well diffusion method, MIC assay, Agar Dilution Method, Spore germination assay	20–25 *μ*g/mL, 3.125–25 mg/mL, 100 mg/mL (15.5–20.5 mm)	Methyl salicylate, Phenolic volatiles, ethanol extract, Pyrolin (He C. et al., 2022)
28	Matilian	*Rheum palmatum* L	*In vitro/In vivo*	MIC assay, MBC assay, Time-kill assay, Crystal violet biofilm assay, Mouse systemic infection model, AFB1 inhibition assay, Spore germination assay	2.34 *µ*M, 8–40 *μ*g/mL, 40 mg/kg	Emodin (Wang J. et al., 2025); Rhein (Wang et al., 2024b)
29	Banzhilian	*Scutellaria barbata* D. Don	*In vitro/In vivo*	Kirby-Bauer test, MSSA, MIC assay, XDR-AB, Time-kill assay, SEM, Membrane integrity assay, Mouse subcutaneous abscess model of XDR-AB infection	6.25–256 *μ*g/mL, 0.78–6.25 mg/mL, 1 mg/disc (10.5–15.2 mm), 125 mg/kg	Volatile oil (Yu et al., 2004); Flavones scutellarein, 4′-hydroxywogonin (Sato et al., 2000); Ethyl acetate fraction, Aqueous extract (Shunqi and Lurong, 2021)
30	Xinyebanzhilian	*Scutellaria indica* L	*In vitro*	Cup-plate method, MIC, and MBC assay	6.25–12.5 mg/mL	Total flavonoids (Juan et al., 2021)
31	Maweilian	*Thalictrum minus* var. *Hypoleucum* (Siebold & Zucc.) Miq	*In vitro*	MIC and MBC assay	4–16 *μ*g/mL	Benzylisoquinoline alkaloid (Mushtaq et al., 2016)
32	Qixinglian	*Viola diffusa* Ging. in DC.	*In vitro*	MIC assay	1–40 *μ*g/mL	Flavonoids and triterpenoids (Zhang C. et al., 2024)

### 3.3 Antioxidant effects

The “Lian” drugs, which exhibit antioxidant properties, contain various bioactive fractions or metabolites (Table 2). These include flavonoids (quercetin glycosides, luteolin-7-O-*β*- D-glucoside, scutellarin), phenolic acids, and polyphenolic metabolites (gallic acid derivatives, tannins, neochlorogenic acid). Except for a small number of single metabolites, most are oversimplified by “total flavonoids/total phenols, with only the content of “total flavonoids” or “total phenols” measured and correlated with antioxidant activity (DPPH scavenging). This approach lacks specificity, making it impossible to determine the truly effective monomers or to rule out synergistic or antagonistic effects among metabolites. The antioxidant mechanisms of the polysaccharide metabolites (acid-assisted polysaccharides, Yam polysaccharides) are usually associated with enhancing endogenous antioxidant enzymes (SOD, CAT) and immune regulation, rather than directly clearing free radicals. Biochemical assays (DPPH, ABTS, FRAP, ORAC, and other *in vitro* chemical assays) are necessary but insufficient for preliminary screening. Very few studies progress to cellular oxidative stress models (H_2_O_2_-induced injury), and even fewer enter animal models to verify their *in vivo* antioxidant efficacy (by detecting SOD, GSH, MDA in liver and brain tissues). The *in vivo* models used include the D-gal aging model, MCAO model, DSS model, and OVX model, which can link antioxidant activity to specific pathophysiological outcomes (cognitive function, infarct size, inflammation, and bone density), thereby significantly strengthening the evidence. However, there are still species differences in animal models, and the vast majority of studies lack data on the pharmacokinetics and bioavailability of metabolites *in vivo*, which is a key bottleneck in translating activity into practical applications.

**TABLE 2:** “Lian” drugs and their antioxidant effects.

Sr No.	“Lian” drugs name	Source	Study	Model/Assay	Conc./Dose range	Effective sites or metabolites
1	Chuanxinlian	*Aconitum sinomontanum* Nakai	*In vitro*	Model of oxidative stress	1–40 *µ*M	Alkaloids and alkaloid salts (Chunyan et al., 2022)
2	Huoxuelian	*Adenocaulon himalaicum* Edgew	*In vitro*	HDFs, HaCaT cells, Antioxidant Measurements, Model of oxidative stress	5–20 μM, 10–50 *μ*g/mL	70% ethanol extract, Neochlorogenic acid (Ahn et al., 2021)
3	Qiyelian	*Aesculus chinensis* Bunge and *Aesculus chinensis* var. *Wilsonii* (Rehder) Turland and N. H. Xia	*In vitro/In vivo*	DSS cycle model, *In vitro* assay	5–10 mg/kg/day	Escin Ia (Yan et al., 2025)
4	Baierlian	*Asparagus cochinchinensis* (Lour.) Merr	*In vitro/In vivo*	D-gal aging model, Model of oxidative stress, APP/PS1 mice	50–200 mg/kg/day	Aqueous extract (Lei et al., 2017); Acid-assisted polysaccharides (Li et al., 2024)
5	Guanyinlian	*Balanophora involucrata* Hook. f	*In vitro*	Network pharmacology analysis	NIL	Phenolic metabolite (She et al., 2013); Polysaccharide (Chao et al., 2024)
6	Dahanliancao	*Bidens tripartita* L	*In vitro*	Biochemical assays	22.55–28.66 *μ*g/mL	Methanol extract (Uysal et al., 2018)
7	Daosilian	*Bistorta amplexicaulis subsp. Sinensis* (F. B. Forbes & Hemsl. ex Steward) Soják	*In vitro*	Biochemical assays	13.40–39.70 *µ*M	5,6-Dihydropyranobenzopyrone (Tantry et al., 2012)
8	Huoxuelian	*Bistorta officinalis* Raf	*In vitro/In vivo*	Biochemical assays, D-gal aging model, tMCAO model	0.10–3.00 mg/mL, 50–400 mg/kg	Total phenolic metabolites, Ethyl acetate extract, N-butanol extract, Flavonoids, Tannins (Yulan et al., 2025)
9	Tiexianlian	*Clematis chinensis* Osbeck	*In vitro*	H/R model, Antioxidant Measurements	6.25–25 *µ*M	Clemaichinenoside (Feng et al., 2020)
10	Babaolian	*Clerodendrum bungei* Steud	*In vitro*	DPPH-HPLC Online Screening Assay, QTOF-MS/MS	58.93 *μ*g/mL	Flavonoids, Phenolic acids, and their derivatives (Qingping and Guanghan, 2018)
11	Niuxuelian	*Dioscorea cirrhosa* Lour	*In vitro*	Model of oxidative stress, Antioxidant, and apoptosis measurements	25–100 *μ*g/mL	Tannin (Zhongjun et al., 2015); Water fraction (Liu et al., 2021)
12	Yeshulian	*Dioscorea polystachya* Turcz	*In vitro/In vivo*	MC3T3-E1 cells, OVX mice	50–200 *μ*g/mL, 100–400 mg/kg/day	Yam polysaccharide (Yu et al., 2025)
13	Bajiaolian	*Dysosma versipellis* (Hance) M. Cheng	*In vitro*	DPPH	7.20–8.50 *µ*M	Flavonols (Sun Y. et al., 2023)
14	Mohanlian	*Eclipta prostrata* (L.) L	*In vitro*	DPPH, Model of oxidative stress	14.21 *µ*M, 10–50 *μ*g/mL	70% ethanol extract, Luteolin-7-O-β-D-glucoside (Yang et al., 2023)
15	Yinxianlian	*Goodyera schlechtendaliana* Rchb. f	*In vitro*	BchE	6.34 *µ*M	Goodyschle A (Dai et al., 2021)
16	Qiyelian	*Gynostemma pentaphyllum* (Thunb.) Makino	*In vitro/In vivo*	CUMS, Model of oxidative stress, Human clinical trial, HEI-OC1 cells, Cisplatin ototoxicity model, Biochemical assays, OFs, GO	12.5–50 μM, 25–100 *μ*g/mL, 0.10–5.00 mg/mL, 10–50 mg/kg/day	Gypenosides (Liu Y. et al., 2025); Polysaccharides (Hu et al., 2025); Extract (Lee J. et al., 2025); Ombuoside (Wu et al., 2024); Gypenoside XLIX (Ping et al., 2025); Saponins (Ma et al., 2022)
17	Baiweilian	*Hemsleya chinensis* Cogn. ex F. B. Forbes & Hemsl	*In vitro*	Biochemical assays	0.10–1.00 mg/mL	Total Alkaloids (Jiang et al., 2023)
18	Duiyuelian	*Hypericum sampsonii* Hance	*In vitro*	Biochemical assays, Model of oxidative stress	10–100 μM, 5–200 *μ*g/mL	Ethanol and hydroalcoholic extracts (Barciela et al., 2025); Ethyl acetate extract, polycyclic polyprenylated acylphloroglucinols (Sun Z. et al., 2023)
19	Yimulian	*Leonurus japonicus* Houtt	*In vitro*	Biochemical assays, HaCaT keratinocytes challenged with H_2_O_2_	12–50 *μ*g/mL	Ethyl acetate fraction, Phenolic, Flavonoids (Zhang L. et al., 2024)
20	Banbianlian	*Lobelia chinensis* Lour	*In vitro*	Biochemical assays	NIL	Scoparone (Kuo et al., 2011)
21	Dabanbianlian	*Lobelia davidii* Franch	*In vitro*	Biochemical assays	0.16–6.15 *µ*M	Lignans (Juelin, 2024)
22	Siyelian	*Marsilea quadrifolia* L	*In vitro/In vivo*	D-gal aging model, Model of oxidative stress, Biochemical assays	28.70–35.20 *μ*g/mL, 25–50 mg/kg/day	C-glycosyl flavones (Zhang et al., 2016)
23	Qikonglian	*Osbeckia stellata* Buch. Ham. ex D. Don	*In vitro*	Biochemical assays	10–500 *μ*g/mL	Extracts (Darmwal et al., 2025)
24	Yanqiaolian	*Persicaria capitata* (Buch.-Ham. ex D. Don) H. Gross	*In vitro*	Biochemical assays, Model of oxidative stress	12.5–200 *μ*g/mL, 0.01–1.60 mg/mL	Ethyl acetate fraction, Flavonoids, Phenolic acids (YUlan et al., 2021)
25	Qiaokelian	*Persicaria chinensis* (L.) H. Gross	*In vitro*	Biochemical assays, Electrochemical detection coupled with HPLC, MS/MS structural identification	10–200 *μ*g/mL, 0.20–1.00 mg/mL	Extract (Wu et al., 2020); Quercetin glycosides, Gallic acid derivatives (Yang et al., 2025); Total phenolics, flavonoids, ethyl acetate fraction, ethanol extract, Aqueous extract (Fengfeng and Hua, 2025)
26	Guanyinzuolian	*Phedimus aizoon* (L.) Hart	*In vitro/In vivo*	Biochemical assays, Model of oxidative stress, STZ mice	10–50 *μ*g/mL, 50–100 mg/kg/day	Phenolic, Flavonoids (Lee et al., 2024); Total Flavonoids (Qi et al., 2022)
27	Luanjiaolian	*Pholidota yunnanensis* Rolfe	*In vitro*	DPPH	28.90 *µ*M	Stilbenes (Dong et al., 2013); 9,10-Dihydrophenanthrene derivatives (Guo et al., 2007)
28	Dujiaolian	*Pinellia pedatisecta* Schott	*In vitro*	Aβ_25-35_-induced neurotoxicity in PC-12 cells	5–20 *µ*M	Alkaloid (Chen et al., 2024)
29	Xionghuanglian	*Pleuropterus ciliinervis* Nakai	*In vitro*	Biochemical assays	1–100 *µ*M	Flavone, Stilbene glycosides (Lee et al., 2003)
30	Shuifulian	*Pontederia crassipes* Mart	*In vitro*	Biochemical assays	0.027–0.096 mg/mL	Total Flavonoids (Ying et al., 2019)
31	Runxuelian	*Pyrola calliantha* Andres	*In vitro*	Biochemical assays, DPPH, HUVEC	6.25–50 *μ*g/mL, 0.02–1.0 mg/mL	Phenolic, Total flavonoids, 70% ethanol extract, Quercitrin, 2′-O-Galloyl-3-β-Galactosyloxy Quercetin (He C. et al., 2022)
32	Matilian	*Rheum palmatum* L	*In vitro/In vivo*	Biochemical assays, Terephthalic acid-induced, Model of oxidative stress, Nephrotoxicity model, Ang II-induced injury in H9c2 cells, Aspergillus flavus, D-gal aging model	5–20 μM, 8–170 *μ*g/mL, 250–1,000 mg/kg, 10–400 mg/kg/day	Aqueous extract (Ma et al., 2024); Chrysophanol (Liu et al., 2025b); Rhein (Wang et al., 2024b); *D*-Galactose (Du et al., 2022)
33	Baihelian	*Saururus chinensis* (Lour.) Baill	*In vitro*	C_2_C1_2_ cell	1–10 *μ*g/mL	Ethanol extract (Eun et al., 2024)
34	Jizhualian	*Sceptridium ternatum* (Thunb.) Lyon	*In vitro*	Standard colorimetric assays, DPPH-HPLC analysis	11.23–21.85 *μ*g/mL	Total phenolic metabolites, Total flavonoids (Zhu et al., 2020)
35	Banzhilian	*Scutellaria barbata* D. Don	*In vitro/In* *vivo*	Model of oxidative stress, Cell lines, Biochemical assays, MI/R model, MCAO/R model, AD mouse models, *C. elegans,* HUVECs	10–50 μM, 25–200 *μ*g/mL, 0.20–1.00 mg/mL, 10–200 mg/kg/day	Scutellarin (Nie et al., 2024); Total flavonoids and polysaccharides (Shunqi and Lurong, 2021)
36	Gouyabanzhilian	*Sedum sarmentosum* Bunge	*In vitro/In vivo*	DOX cardiotoxicity model, DOX-induced injury in H9c2 cells, High-fat diet-induced fatty liver model in Nile tilapia, Model of oxidative stress, Biochemical assays	5–20 μM, 0.10–1.00 mg/mL, 5–10 mg/kg/day, 0.5–1.0 g/kg diet	Sarmentosin (Lin et al., 2024); Total flavanones (Yu et al., 2021); Total flavonoids, Total polyphenols (Ying-Ying et al., 2020)
37	Bagualian	*Sinopodophyllum hexandrum* (Royle) T. S. Ying	*In vitro/In vivo*	Biochemical assays, Whole-body gamma-irradiation model, OVX mice	9.80 *μ*g/mL, 15–400 mg/kg, 100–200 mg/kg/day	Extract (Anand et al., 2022); Total phenolic metabolites, Total flavonoids (Liu W. et al., 2022); ethanol extract (Tan et al., 2025); Aqueous extract (Ganie et al., 2012)
38	Bixuelian	*Stephania japonica* (Thunb.) Miers	*In vitro/In vivo*	Scopolamine mode, Biochemical assays, tMCAO, Model of oxidative stress, OGD/R	1–50 μM, 10–320 *μ*g/mL, 5–20 mg/kg, 100–400 mg/kg/day	Chloroform fraction (Al-Amin et al., 2022); Stepharine (Hao et al., 2020); Ceparanthine (Lin et al., 2019)
39	Jingoulian	*Uncaria sinensis* (Oliv.) Havil	*In vitro/In vivo*	HDFs, Biochemical assays, D-gal aging model, Free radical-induced erythrocyte hemolysis assay, DPPH	1–50 μM, 1–100 *μ*g/mL, 0.1–1.0 mg/mL	Phenolic metabolites (Na et al., 2004); Extract (Sekiya et al., 2002)
40	Shuikulian	*Veronica anagallis-aquatica* L	*In vitro/In vivo*	Biochemical assays, ISO model, Model of oxidative stress	10–200 *μ*g/mL, 10–200 mg/kg/day	Phenolic acids, Flavonoids (Vrca et al., 2024); Iridoid glycosides, Gut microbiota-mediated metabolites (Wang M. et al., 2025)

### 3.4 Anti-tumor effects

The “Lian” drugs, along with their bioactive fractions or metabolites that exhibit anti-tumor effects, are shown in Supplementary Table 3. Flavonoids (quercetin, luteolin, scutellarin, kaempferol-3-O-glucoside) are commonly used in CCK-8, flow cytometry, WB, and CDX models. *In vivo* effects are often weaker than *in vitro* effects due to low bioavailability and rapid metabolism. Although the mechanisms are well studied, low bioavailability makes it difficult to achieve therapeutic concentrations. Terpenoids and their derivatives (triterpenoid saponins, steroid saponins, gypenosides, oleanolic acid, curcurbitacin) have poor oral absorption, unclear *in vivo* metabolism, high toxicity, and a narrow therapeutic index. Cucurbitacin, a classic inhibitor of the JAK2/STAT3 signaling pathway, induces cancer cell apoptosis. Alkaloids such as lappaconitine, cephalantine, and sinomontanine are commonly used for cytotoxicity testing of human cancer cell lines (MTT/CCK-8) and CDX models. Their toxicity to the heart and nervous system may limit their therapeutic window, and they lack targeted validation, have low bioavailability, and require formulation optimization or structural modification. Most studies on lignans, such as podophyllotoxin, sauchinone, and machilin, have been limited to the extract level, and the mechanisms of individual metabolites have not been thoroughly studied. Podophyllotoxin is a classic example of a natural product successfully transformed into modern drugs. Still, its high toxicity also warns us that potent anti-cancer activity often comes with serious side effects. Currently, most research is still at the stage of phenotype observation, lacking in-depth exploration of mechanisms and assessment of transformation potential. Future research should focus on target identification, structural optimization, pharmacokinetic improvement, and systematic analysis of mechanism networks for monomeric metabolites, and on validating these findings using more clinically relevant models.

### 3.5 Antiviral effects

The antiviral effects of “Lian” drugs derived from botanical sources, along with their specific active fractions or pure metabolites, are noteworthy (Table 3). Flavonoids generally exert *µ*M-level activity by inhibiting viral entry (HA/NA/ACE2) and host protease (protease L), which reflects their non-specific effects. They may interfere with multiple viral life processes through slight membrane stabilization, antioxidant effects, or non-specific protein binding. However, in the human body, the concentrations of their original metabolites never reach the adequate micromolar levels observed *in vitro*. In addition, extracts account for a large proportion, and their scientific value is limited to suggesting that the source is worth further research. The vast majority of studies use laboratory-adapted viral strains in single, highly susceptible cell lines (Vero or MDCK), which are far from the complex multicellular environment, immune system stress, and the behavior of clinical isolates in the human body. The current research on plant antiviral metabolites is severely limited, and future breakthroughs will focus on chemical transformations from crude extracts to specific monomers and on biological transformations from simple cell models to physiologically relevant systems as precursors.

**TABLE 3:** “Lian” drugs and their antiviral effects.

Sr No.	“Lian” drugs name	Source	Study	Model/Assay	Conc./Dose range	Effective sites or metabolites
1	Dierlian	*Asparagus filicinus* D. Don	*In vitro*	Virus-induced cytopathic effect inhibition assay, Cytotoxicity assay	1–25 *μ*g/mL	Extracts (Rajbhandari et al., 2009)
2	Guanyinlian	*Balanophora involucrata* Hook. f	*In vitro*	Neuraminidase inhibition assay, Cytotoxicity assays, Cell-based virus replication assay, CCK-8 assay, Molecular docking, Plaque reduction assay	0.39–100 μM, 0.1–100 *μ*g/mL	Epicatechin-3-O-gallate, (+)-Catechin (Sun X. et al., 2020); Extract (Yang et al., 2016)
3	Huoxuelian	*Bistorta officinalis* Raf	*In vitro*	Cytopathic effect inhibition assay	50–100 *μ*g/mL	Extracts (Yulan et al., 2025)
4	Bajiaolian	*Dysosma versipellis* (Hance) M. Cheng	*In vitro/In vivo*	Toxicity model, CCK-8 assay, Flow cytometry, WB analysis, Bioaffinity ultrafiltration-HPLC/MS, Computational (*in silico*) study, HIV-1 integrase (IN)-LEDGF/p75 interaction inhibition assay, HIV-1 replication assay, MTT assay	100–400 nM, 0.02–11.24*µ*M, 4.52–7.34 *μ*g/mL, 1.0–2.0 mg/kg/day	Podophyllotoxin (Liu et al., 2025a); Podophyllotoxin, Diphyllin (Feng et al., 2022); Endophytic fungal (Zhou et al., 2019); Flavonol dimers (Chen et al., 2015)
5	Honghanlian	*Hypericum ascyron* L	*In vitro*	Cell-based virus-induced cytopathic effect reduction assay, Cytotoxicity assays	20 *µ*M	Polyprenylated spirocyclic acylphloroglucinol derivatives (PSAPs) (Zhu et al., 2015)
6	Duiyuelian	*Hypericum sampsonii* Hance	*In vitro/In vivo*	Cell-based virus replication assay, Cytopathic effect inhibition assay, Plaque reduction assay, MTT/CCK-8 assay, Time-of-addition assay, Neuraminidase inhibition assay, Hemagglutination inhibition assay, Molecular docking, H5N1-infected mouse model	0.1–100 μM, 0.1–5 mg/kg, 20–100 mg/kg/day	Polycyclic polyprenylated acylphloroglucinols, Flavonoids, Phloroglucinols, Xanthones, Hypericin (Sun Z. et al., 2023)
7	Banbianlian	*Lobelia chinensis* Lour	*In vitro/In vivo*	Plaque reduction assay, MTT assay, HSV-1 infected mouse model of cutaneous infection	1.60–180 *μ*g/mL, 40–80 mg/kg/day	Ethyl acetate fraction, Water fraction (Kuo et al., 2008)
8	Qiaokelian	*Persicaria chinensis* (L.) H. Gross	*In vitro*	Cytopathic effect reduction assay, Plaque reduction assay, Time-of-addition assay, Hemagglutination inhibition assay, Neuraminidase inhibition assay, Molecular docking, CCK-8 assay	1.56–100 *µ*M	Flavonoid glucuronides, Dicaffeoylquinic acids (Fengfeng and Hua, 2025)
9	Runxuelian	*Pyrola calliantha* Andres	*In vitro*	Cell-based virus replication assay, CCK-8 assay, Molecular docking, Plaque reduction assay	0.1–100 *μ*g/mL	Extract (Yang et al., 2016); Ethyl acetate extract (He C. et al., 2022)
10	Matilian	*Rheum palmatum* L	*In vitro*	MTT assay, IFA	46.28–1,174.00 *μ*g/mL	Polysaccharide (Du et al., 2022)
11	Baihelian	*Saururus chinensis* (Lour.) Baill	*In vitro/In vivo*	Cell-based Virus replication assays, Plaque reduction assay, MTT assay, WB analysis, IFA, qRT-PCR, Time-of-addition assay, Virus attachment and internalization assays, Cellular thermal shift assay, EV71-infected suckling mouse model, CCK-8 assay, Enzyme-linked immunosorbent assay, CVB3-induced viral myocarditis mouse model	0.38–2 μM, 1.02–102.4 *μ*g/mL, 1–40 mg/kg/day	Ethyl acetate fraction (Wang et al., 2015); Manassantin B (Song et al., 2019); Saucerneol (Song et al., 2023)
12	Banzhilian	*Scutellaria barbata* D. Don	*In vitro/In vivo*	Lytic replication inhibition assay, WB analysis, MTT assay, Mechanistic insight assays, Plaque reduction assay, qRT-PCR, Protease activity assay, SARS-CoV-2 infected mouse model, Cell-based HIV-1 infection assay, HIV-1 pseudovirus entry assay, Host protease (Cathepsin L) inhibition assay, Molecular docking, Time-of-addition assay	1–200 μM, 50–600 *μ*g/mL, 1.5 g/kg/day	Neo-clerodane diterpenoids (Wu et al., 2015); Extracts (Ran et al., 2025); Flavonoids (Tang et al., 2023)
13	Bagualian	*Sinopodophyllum hexandrum* (Royle) T. S. Ying	*In vitro*	Cell-based virus yield reduction assay, Viral DNA synthesis inhibition assay, Cytotoxicity assay, Time-of-addition assay, Enzymatic catalysis model, Microbial production model	8.75 *µ*M, 0.05–0.5 *μ*g/mL	Etoposide (Nishiyama et al., 1987); Podophyllotoxin (Liu W et al., 2025); Lignan (Decembrino et al., 2021)
14	Bixuelian	*Stephania japonica* (Thunb.) Miers	*In vitro/In vivo*	Cell-based virus replication assay, Plaque reduction assay, IFA, WB analysis, qRT-PCR, Time-of-addition assay, Virus attachment and internalization assays, Molecular docking, PEDV-infected piglet model	1.56–25 μM, 7.938–241.1 *μ*g/mL, 250 mg/kg/day	Extract, Alkaloids (Zhao Y. et al., 2025); Cepharanthine (Weiyang and Lanjuan, 2023)
15	Shuihuanglian	*Swertia davidii* Franch	*In vitro*	Computational (*in silico*) study	NIL	Sweroside, Swertiamarin (Wang W. et al., 2024)
16	Qixinglian	*Viola diffusa* Ging. in DC.	*In vitro*	Computational genomic sequencing and assembly model	NIL	Flavonoids and triterpenoids (Zhang C. et al., 2024)

### 3.6 Insecticidal effects

Table 4 presents the “Lian” drugs known for their insecticidal effects, along with their active fractions or metabolites. According to the records, 10 distinct “Lian” drugs show insecticidal effects. The primary insecticidal metabolite responsible for these effects is alkaloids. This is highly consistent with the ecological strategy of plant defense against insect feeding. Alkaloids (yajiaguan delcosine, belsoline, lepene, demethylenedelcorine, 18-O-methyligactonine) are the most important class of food repellents, gastric toxins, and nerve agents in the plant kingdom. They are central to research on natural insecticidal activity. Their mechanism is to block the transmission of nerve impulses, leading to paralysis and death. However, their most significant limitation is poor selectivity and an extremely high risk to non-target organisms, which severely limits their prospects for direct application as insecticides. Terpenoid metabolites (*E*)-*β*-farnesene, *α*-pinene, and oleic acid are typically highly volatile and primarily affect behavioral regulation and neurotoxicity. Their fumigation and contact-killing effects are usually non-selective and may harm pollinating insects such as bees and natural enemies. The study of their mechanisms of action usually remains at the behavioral level, lacking precise molecular target evidence. Model singularity, with the vast majority of studies using only 1-2 laboratory model insects, makes it difficult to predict their actual effects on complex pest populations in the field and lacks safety evaluations for non-target organisms such as natural enemies and pollinating insects. The confusion over dosage concepts has led many studies to provide only concentration gradients during screening, without calculating key toxicological parameters or comparing them with those of commercial insecticides, thereby preventing objective evaluation of their activity levels. Future research will focus on systematically studying the structure-activity relationships of the discovered highly active lead metabolites and on optimizing their efficacy, selectivity, and stability through chemical modification. Research on sustained-release technologies, such as microencapsulation and nanocarriers, for volatile and biodegradable metabolites to extend their shelf life, reduce usage frequency, and dosage.

**TABLE 4:** “Lian” drugs and their insecticidal effects.

Sr No.	“Lian” drugs name	Source	Study	Model/Assay	Conc./Dose range	Effective sites or metabolites
1	Chuanxinlian	*Aconitum sinomontanum* Nakai	*In vitro*	Leaf disc choice bioassay	1 mg/mL	Yajiaguang delcosine, Belsoline, Lepenine (Zhang et al., 2022); Demethylenedelcorine, and 18-*O*-Methylgigactonine (Yuan and Wang, 2012)
2	Dengtailian	*Arisaema heterophyllum* Blume*, Arisaema erubescens* (Wall.) Schott and *Arisaema amurense* Maxim	*In vitro*	Direct nematocidal assay, Cell viability, Cytotoxicity assay, Apoptosis detection assays, Hoechst 33,258 staining, Annexin V-FITC/PI double staining	10–320 *μ*g/mL	Flavone-C-glycosides (Du et al., 2011); Total alkaloids (Lianrui and Mingsan, 2023)
3	Shuihuanglian	*Boenninghausenia albiflora* (Hook.) Reichb. ex Meisn	*In vitro*	Topical application bioassay	3.125–50 *µ*g/insect	Prenylated coumarin (Sharma et al., 2006)
4	Babaolian	*Clerodendrum bungei* Steud	*In vivo*	Behavioral bioassay (area preference test) on live insects	0.13–78.63 nL/cm^2^	(E)-β-farnesene, α-pinene (Lu et al., 2021)
5	Guanyinlian	*Dioscorea zingiberensis* C. H. Wright	*In vivo*	WHO larval bioassay, Goldfish immersion therapy model	0.5–25.0 *μ*g/mL, 1.0–3.0 mg/L	Spirobisnaphthalene metabolites (Tian et al., 2016); Ethanolic extracts (Zhang et al., 2018)
6	Dujiaolian	*Pinellia pedatisecta* Schott	*In vivo*	Non-choice bioassay, Antibiosis assay, Host preference (choice) test	NIL	Pinellia pedatisecta agglutinin (Zhao et al., 2022)
7	Xionghuanglian	*Pleuropterus ciliinervis* Nakai	*In vivo*	Plasmodium berghei-Infected mouse model	5 mg/kg/day	(E)-Resveratrol 3-O-β-D-glucopyranoside (Moon and Sim, 2008); (E)-Resveratrol 3,4′-O-β-D-diglucopyranoside (Lee et al., 2008)
8	Shuifulian	*Pontederia crassipes* Mart	*In* *vivo*	Leaf-dip bioassay, In silico molecular docking	625–10000ppm	N-hexane fraction (Abdelkhalek et al., 2022)
9	Bagualian	*Sinopodophyllum hexandrum* (Royle) T. S. Ying	*In vivo*	WHO larval bioassay	0.79–12.50 *μ*g/mL	Podophyllotoxin, Methyl podophyllate (Maleck et al., 2017)
10	Tushanhuanglian	*Thalictrum javanicum* Blume	*In vivo*	HBT, WHO larval bioassay	0.50–6.00 mg/cm^2^	Oleic acid (Gurunathan et al., 2016)

### 3.7 Antidiabetics effects

Table 5 lists the “Lian“-derived antidiabetic drugs and their active fractions or pure metabolites. The data indicate that 17 different “Lian” drugs exhibit antidiabetic effects, primarily due to flavonoids and saponins (escins/escigens, dioscin, gypsides, dammarane-type triterpenoids), which have been widely shown to significantly improve insulin sensitization, glucose and lipid metabolism, and more. The frequent occurrence of flavonoids (quercetin, luteolin, kaempferol, apigenin) and water/alcohol extracts highlights the multitarget regulatory properties of flavonoids in metabolic diseases, reflecting direct research on traditional decoctions or tinctural forms. Natural products are often used as modulators with slow onset and weak efficacy, making it difficult to compete with potent chemical hypoglycemic drugs (such as insulin and sulfonylureas) for acute hypoglycemic effects. Moreover, the depth of the mechanism is insufficient, and many studies focus only on phenotype observation (lowering blood sugar and improving glucose tolerance), lacking in-depth exploration of exact molecular targets, signaling networks, and long-term medication safety. Therefore, natural products are more suitable for intervention at the early stage of diabetes or as adjunctive treatment for type-2 diabetes, combined with first-line drugs, thereby reducing the need for chemical drugs and their side effects. Taking specific saponins as lead metabolites and developing new insulin sensitizers with higher safety is one of the most promising directions at present.

**TABLE 5:** “Lian” drugs and their anti-diabetic effects.

Sr No.	“Lian” drugs name	Source	Study	Model/Assay	Conc./Dose range	Effective sites or metabolites
1	Qiyelian	*Aesculus chinensis* Bunge and *Aesculus chinensis* var. *Wilsonii* (Rehder) Turland and N. H. Xia	*In vitro/In vivo*	α-Glucosidase inhibition assay, STZ-induced diabetes model, Molecular mechanism analysis	1–50 μM, 5–50 mg/kg/day	Escins, Aescigens family (Mei et al., 2024)
2	Guanyinlian	*Angiopteris evecta* (G. Forst.) Hoffm	*In vitro/In vivo*	Glucose uptake assay in muscle cells, Oral glucose tolerance test	1 mg/mL, 500 mg/kg/day	Aqueous extract (Hoa et al., 2009)
3	Babaolian	*Clerodendrum bungei* Steud	*In vitro*	α-Glucosidase inhibition assay, ACE inhibition assay	121.2–654.4 *µ*M	Phenylethanoid glycoside, Diterpenoid (Liu Q. et al., 2014)
4	Yeshulian	*Dioscorea polystachya* Turcz	*In vitro/In vivo*	STZ-induced diabetes model, High-glucose-stimulated podocyte model	2.5–10 μM, 40–80 mg/kg/day	Dioscin (Zong Y. et al., 2022)
5	Mohanlian	*Eclipta prostrata* (L.) L	*In vitro/In vivo*	STZ-induced diabetes model, Biochemical analysis of tissues, Enzyme inhibition assays, α-Glucosidase inhibition assay, Aldose reductase inhibition assay, Oral starch tolerance test	0.60–4.20 *µ*M, 5–400 mg/kg/day	Methanol extract (Feng et al., 2019); Eclalbasaponin VI (Feng et al., 2019); Wedelolactone (Timalsina and Devkota, 2021); Ethanolic extract, Demethylwedelolactone (Timalsina and Devkota, 2021)
6	Qiyelian	*Gynostemma pentaphyllum* (Thunb.) Makino	*In vitro/In vivo*	Network pharmacology analysis, Molecular docking, HFD and STZ-induced diabetes model, Insulin-sensitive cells, Recombinant human PTP1B enzyme inhibition assay, Cellular glucose uptake assay	1–50 μM, 50–300 mg/kg/day	Extract (Yang et al., 2024); Flavonoids and saponins (Xie et al., 2024); Gypenosides (Lee Y. et al., 2025); Dammarane-type triterpenoid saponins (Wang et al., 2024a)
7	Duiyuelian	*Hypericum sampsonii* Hance	*In vitro*	α-Glucosidase inhibition assay, Molecular docking	0.43–1.27 *µ*M	Polyprenylated acylphloroglucinols, Phloroglucinol derivatives (Tao et al., 2023)
8	Yimulian	*Leonurus japonicus* Houtt	*In vitro/In vivo*	STZ-induced diabetes model, Glomerular endothelial cells, Human umbilical vein endothelial cells	5–50 μM, 5–20 mg/kg/day	Leonurine (Yu et al., 2024)
9	Banbianlian	*Lobelia chinensis* Lour	*In vitro*	metabolite screening and target prediction, Network construction and analysis, Molecular docking, α-Glucosidase inhibition assay	29–170 *µ*M	Quercetin, Luteolin, Kaempferol, Apigenin (Ge et al., 2020); Pyrrolidine alkaloids (Shibano et al., 2001)
10	Dabanbianlian	*Lobelia davidii* Franch	*In vitro*	α-Glucosidase inhibition assay	11.41–225.03 *µ*M	Triterpenoids and alkaloids (Juelin, 2024)
11	Siyelian	*Marsilea quadrifolia* L	*In vivo*	Alloxan-induced diabetic rats	200–400 mg/kg/day	Aqueous extract (Karikalan and Rajangam, 2018)
12	Yanqiaolian	*Persicaria capitata* (Buch. Ham. ex D. Don) H. Gross	*In vitro/In vivo*	Molecular mechanism analysis, db/db mice, α-Glucosidase inhibition assay	15–50 μM, 2–8 g/kg/day	Aqueous extract, Lignans (YUlan et al., 2021)
13	Guanyinzuolian	*Phedimus aizoon* (L.) ‘t Hart	*In vivo*	STZ-induced diabetes model	50–100 mg/kg/day	Total flavonoids (Qi et al., 2022)
14	Jixuelian	*Pronephrium penangianum* (Hook.) Holttum	*In vitro*	Spectroscopic methods	NIL	Flavan-4-ol glycosides (Zhao et al., 2006)
15	Matilian	*Rheum palmatum* L	*In vivo*	HFD and STZ-induced diabetes model	100–200 mg/kg/day	Polysaccharide (Du et al., 2022)
16	Baihelian	*Saururus chinensis* (Lour.) Baill	*In vivo*	STZ-induced diabetes model	100–500 mg/kg	Lignans (Chen et al., 2022)
17	Banzhilian	*Scutellaria barbata* D. Don	*In vivo*	HFD-induced diabetes model	1–4 g/kg/day	Alcohol extract (Le et al., 2024)

### 3.8 Hepatoprotective effects

Table 6 presents a detailed overview of “Lian”-derived drugs with hepatoprotective effects, including their active fractions or pure metabolites. The data show that 16 “Lian” drugs possess hepatoprotective effects. Mechanistic research on saponin metabolites (aescin, dioscin, gypenosides, echinocystic acid, eclalbasaponin II) has progressed from simple antioxidant effects to energy metabolism, organelle homeostasis, and cell fate regulation, aligning more closely with the modern understanding of liver disease pathophysiology. Hepatoprotective activity has been confirmed in chemical (CCl_4_, acetaminophen), alcoholic, and immune liver injury models. However, oral bioavailability is low, and some saponins may exhibit hemolytic or hepatorenal toxicity at high doses, resulting in a “double-edged sword” effect. Its effectiveness and safety window in the human body need to be strictly defined; The core mechanism of flavonoids is antioxidant stress. These metabolites have significant free-radical scavenging effects and can directly neutralize reactive oxygen species (ROS). The Nrf2 pathway has become a “classic” target for liver protection by these metabolites, and the evidence chain is relatively complete. However, most studies have focused on total flavonoids or individual known metabolites. There is a lack of systematic comparison of how glycosylation, acylation, and other modifications affect bioavailability, target affinity, and overall efficacy. In addition, their antioxidant effects may be weakened in complex *in vivo* environments, and high doses may produce pro-oxidative effects. The model is too simple, and a large number of studies rely on acute chemical liver injury models induced by CCl_4_ or acetaminophen, which differ from the pathogenesis of major chronic liver diseases in humans (fatty liver, viral hepatitis, and alcoholic liver). Advanced *in vitro* models should be vigorously promoted and standardized, and liver-like organs and microfluidic liver chips should be included in the drug screening and mechanism research system to evaluate the comprehensive effects of metabolites in multidimensional pathological processes such as metabolism, inflammation, and fibrosis.

**TABLE 6:** “Lian” drugs and their hepatoprotective effects.

Sr No.	“Lian” drugs name	Source	Study	Model/Assay	Conc./Dose range	Effective sites or metabolites
1	Qiyelian	*Aesculus chinensis* Bunge and *Aesculus chinensis* var. *Wilsonii* (Rehder) Turland and N. H. Xia	*In vitro/In vivo*	Free fatty acid-induced hepatocyte steatosis and lipotoxicity, Gene knockdown, High-fat diet-induced NAFLD mouse model	5–10 *μ*g/mL, 10–20 mg/kg/day	Aescin (Yu et al., 2023)
2	Leigonglian	*Amydrium sinense* (Engl.) H. Li	*In vitro/In vivo*	TGF-β1-induced activation of hepatic stellate cells, Mechanistic validation using a Stat3 agonist, Carbon tetrachloride-induced liver fibrosis in mice	25–100 *μ*g/mL, 100–200 mg/kg/day	Aqueous extract (Li et al., 2023)
3	Guanyinlian	*Balanophora involucrata* Hook. f	*In vivo*	D-galactose-induced sub-acute aging and liver injury model	100–200 mg/kg/day	Polysaccharide (Zhang T. et al., 2021)
4	Yeshulian	*Dioscorea polystachya* Turcz	*In vivo*	High-fat diet/high-sucrose diet combined with low-dose streptozotocin-induced type II diabetes and NAFLD in rats, Primary hepatocytes isolated from the experimental rats	50 and 100 mg/kg/day	Dioscin (Zhong Z. et al., 2022)
5	Mohanlian	*Eclipta prostrata* (L.) L	*In vitro/In vivo*	Toxin-induced cytotoxicity in human liver cells, Concanavalin A-induced immune-mediated hepatitis in mice, LPS-stimulated inflammation in murine macrophages, Cell proliferation assay, Cytotoxicity assay	10–50 μM, 5–10 mg/kg	Bithiophene derivatives, Coumestan derivatives (Giang et al., 2024); Wedelolactone (Feng et al., 2019); Echinocystic acid and eclalbasaponin II (Feng et al., 2019); Ethanolic extract (Timalsina and Devkota, 2021)
6	Yinxianlian	*Goodyera schlechtendaliana* Rchb. f	*In vitro*	D-galactosamine-induced cytotoxicity in primary rat hepatocytes	0.1–1.0 mM	Goodyeroside A (Du et al., 2008); Aliphatic glycosides (Du et al., 2000)
7	Qiyelian	*Gynostemma pentaphyllum* (Thunb.) Makino	*In vitro/In vivo*	*α*-Naphthyl isothiocyanate-induced cholestatic liver injury in rats, Palmitic acid-induced lipid accumulation and lipotoxicity in human liver cells, Methionine-choline deficient diet-induced NASH mouse model, Carbon tetrachloride-induced acute liver injury in mice, LO2 human normal hepatocyte cell, In silico models	10–50 μM, 100 *μ*g/mL, 10–200 mg/kg/day	Gypenosides (Zhang Z. et al., 2025); Gypenoside XIII (Cheng et al., 2024); Flavonoids and saponins (Xie et al., 2024); Water extract (Hu et al., 2024)
8	Honghanlian	*Hypericum ascyron* L	*In vitro*	D-galactosamine-induced cytotoxicity in primary rat hepatocytes, APAP-induced hepatotoxicity in human liver cells	1.25–20 *µ*M	Hyperascyrins (Zhen et al., 2019)
9	Yimulian	*Leonurus japonicus* Houtt	*In vitro/In vivo*	High-fat diet-induced NAFLD mouse model, PA-induced hepatocyte steatosis model, ADRA1a-overexpression model, APAP-induced hepatotoxicity in human liver cells, APAP-induced acute liver injury mouse model	1.25–80 μM, 50 mg/kg, 5–20 mg/kg/day	Leonurine (Fan et al., 2024); Dibenzocyclooctadiene lignans (Tian et al., 2021)
10	Jiujielian	*Peristrophe japonica* (Thunb.) Bremek	*In vitro/In vivo*	HBV-transfected human hepatoma cell, Primary human hepatocytes infected with HBV, Hydrodynamic injection mouse model of HBV persistence	1–20 μM, 5–20 mg/kg	Ciliatoside A (Fang et al., 2023)
11	Qiaokelian	*Persicaria chinensis* (L.) H. Gross	*In vitro/In vivo*	Acetaminophen-induced cytotoxicity in human liver cells, Acetaminophen-induced acute liver injury in mice, HepG2 human hepatoma cells inflammation model	10–100 *μ*g/mL, 100–200 mg/kg/day	Total flavonoids (Xu et al., 2019); Methanol extract (Hossen et al., 2015)
12	Runxuelian	*Pyrola calliantha* Andres	*In vitro/In vivo*	Tert-butyl hydroperoxide-induced oxidative damage in human liver cells, RNA interference, and carbon tetrachloride-induced acute oxidative liver injury in mice	25–50 μM, 25–50 mg/kg/day	2′-*O*-alloylhyperin (Wang et al., 2017)
13	Matilian	*Rheum palmatum* L	*In vitro/In vivo*	Hepatotoxicity assessment, Anti-fibrotic mechanism, Acute liver injury model, Carbon tetrachloride-induced liver fibrosis in mice	10–30 μM, 10–20 mg/kg/day	Emodin (Guo Y. et al., 2024)
14	Baihelian	*Saururus chinensis* (Lour.) Baill	NIL	NIL	NIL	Extract (Eun et al., 2024)
15	Banzhilian	*Scutellaria barbata* D. Don	*In vivo*	Carbon tetrachloride-induced acute chemical liver injury in mice	100–200 mg/kg/day	Polysaccharide (Shunqi and Lurong, 2021)
16	Gouyabanzhilian	*Sedum sarmentosum* Bunge	*In vitro/In vivo*	FXR reporter gene assay, Taurocholic acid-induced cytotoxicity in hepatocytes, Alpha-naphthyl isothiocyanate-induced cholestatic liver injury in mice, High-fat diet-induced fatty liver disease in fish, Carbon tetrachloride-induced cytotoxicity and oxidative damage in human liver cells	25–200 *μ*g/mL, 50–200 mg/kg, 200–400 mg/kg/day	Extract (Hao et al., 2022); Ethyl acetate extract (Liu Z. et al., 2022); Total flavanones (Yu et al., 2021); Aqueous extract (Zhu et al., 2021); Glycoside (Ying-Ying et al., 2020)

### 3.9 Brain protective effects

Table 7 provides a detailed overview of “Lian”-derived drugs that show efficacy in offering cerebral protection, along with their active fractions or metabolites. The data presented in the table indicate that six “Lian” drugs exhibit cerebral protective activity. However, no clear pattern has been identified among the metabolites responsible for these effects. Only certain alkaloids (stachydrine, indole, oxindole) can directly act on the central nervous system, penetrate the blood-brain barrier, and affect neurotransmitters, receptors, and ion channels. However, alkaloids exert a strong regulatory effect on the nervous system, making their treatment window usually very narrow. For example, alkaloids that act on the dopamine system may induce psychiatric symptoms or motor disorders. The neurotoxic risk of excessive sedation or dependence caused by actions on the GABA system must be rigorously evaluated. More extracts are used as active metabolites, and their mechanisms of action are mainly described in terms of phenotypes such as “antioxidant stress” and “anti-inflammatory”, which reflect the comprehensive effects of multiple metabolites. However, it remains unclear which metabolites achieve this, as the signaling pathways are poorly explored. Moreover, most studies in the literature use young, healthy male animals, ignoring the impact of key biological variables such as age and gender on stroke outcomes, which reduces the potential for clinical translation. In the future, activity-tracking methods should be adopted to isolate and identify the true key functional metabolites from crude extracts. Metabolomics and network pharmacology methods should be used to explore interactions within the metabolite-target group pathway network, establish quality control standards based on key marker metabolites, and ensure the reproducibility of experimental materials.

**TABLE 7:** “Lian” drugs and their brain protective effects.

Sr No.	“Lian” drugs name	Source	Study	Model/Assay	Conc./Dose range	Effective sites or metabolites
1	Guanyinlian	*Dioscorea zingiberensis* C. H. Wright	*In vivo*	OGD/R, MCAO	0.1–20 μM, 20 mg/kg	Deltonin (Zhang Y. et al., 2020)
2	Mohanlian	*Eclipta prostrata* (L.) L	*In vivo*	BCCAO with reperfusion, Scopolamine-induced amnesia model, and aged rat model of cognitive deficits	100–400 mg/kg/day	Hydroalcoholic extract, Luteolin (Timalsina and Devkota, 2021)
3	Qiyelian	*Gynostemma pentaphyllum* (Thunb.) Makino	*In vitro/In vivo*	Primary mouse microglial cells, Mouse brain microvascular endothelial cells, Mouse model of sepsis-associated encephalopathy, OGD/R, CCK-8 assay, Annexin V-FITC/PI staining, JC-1 staining, WB analysis, tMCAO model	0.1–20 μM, 10–40 mg/kg	Gypenoside XLIX (Zhao P. et al., 2025); Gypenoside XVII (Xie et al., 2022)
4	Yimulian	*Leonurus japonicus* Houtt	*In vitro/In vivo*	Primary cortical neurons or neuron-like cells, Microglial cells, Brain microvascular endothelial cells, MCAO model of cerebral I/R injury, Global cerebral ischemia model, Intracerebral hemorrhage model, Neuroinflammatory and neurodegenerative model, Cognitive impairment model	10–500 μM, 20–80 mg/kg, 50–200 mg/kg/day	Stachydrine (Liao et al., 2023)
5	Shuifulian	*Pontederia crassipes* Mart	*In vivo*	I/R Model	200–400 mg/kg/day	Extract (Bhavsar et al., 2020)
6	Jingoulian	*Uncaria sinensis* (Oliv.) Havil	*In vitro/In vivo*	Glutamate-induced excitotoxicity, BCCAO, Photothrombotic cortical ischemia model	0.1–10 μM, 50–250 mg/kg/day	Phenolic metabolites, Procyanidin oligomers, Alkaloids (Shimada et al., 2001; Yokoyama et al., 2004); Indole, Oxindole alkaloids (Shimada et al., 1999); Hexane extracts (Park et al., 2011)

### 3.10 Neuroprotective effects

The “Lian” drugs, known for their neuroprotective effects and active sites or metabolites, are detailed in Table 8. Among them, saponin metabolites (aescin/escin, deltonin, steroid saponins, eclalbasaponin II, gypenosides, ombuoside, gypenoside XVII) exhibit strong anti-inflammatory, antioxidant, and anti-apoptotic activities and can protect neurons through multiple pathways. Saponins are polar macromolecules with an extremely weak ability to penetrate. The neuroprotective effects observed in animal models are likely due to their strong peripheral anti-inflammatory and antioxidant effects, which improve the systemic environment, indirectly reduce the stress load on the central nervous system, or enter pathological models of BBB damage (cerebral ischemia and trauma). For an intact blood-brain barrier (BBB), their ability to achieve effective concentrations in the brain is questionable, severely limiting their potential as direct therapeutic drugs for central nervous system diseases. Many studies have focused only on detecting changes in the expression of a few classic pathway proteins (p-Akt, Bcl-2/Bax), and have not revealed which metabolites in the extract, which upstream receptors or sensors activate this signal, or which disease models use a single approach and do not match clinical heterogeneity. All current studies are mostly acute or subacute intervention experiments, lacking evaluation of the toxicity, tolerability, and long-term neuroprotective effects of metabolites under long-term administration, which is a gap that must be filled before clinical translation.

**TABLE 8:** “Lian” drugs and their neuroprotective effects.

Sr No.	“Lian” drugs name	Source	Study	Model/Assay	Conc./Dose range	Effective sites or metabolites
1	Qiyelian	*Aesculus chinensis* Bunge and *Aesculus chinensis* var. *Wilsonii* (Rehder) Turland and N. H. Xia	*In vitro/In vivo*	mHTT-induced neurotoxicity model (in HT22 cells), CUMS model, Molecular pathway analysis in brain tissue, CCI model	0.5–4 μM, 1–40 mg/kg/day	Triterpenoid saponin (Sun Y. et al., 2023): Aescin (Liu et al., 2024); Phenylethanol glycosides (Zhang N. et al., 2020); Escin (Zhang L. et al., 2020)
2	Baierlian	*Asparagus cochinchinensis* (Lour.) Merr	*In vitro/In vivo*	Glutamate-induced excitotoxicity model (in primary neurons), MTT assay, Hoechst 33,342 staining, WB analysis, Gerbil ischemia model	0.1–10 *μ*g/mL, 200 mg/kg/day	Extract (Jalsrai et al., 2016); Phenols, saponins, and protodiosgenin (Wang M. et al., 2022)
3	Zhuyetiexianlian	*Clematis terniflora* DC.	*In vitro*	Corticosterone-induced neurotoxicity model, MTT assay, Hoechst 33,342 staining, Annexin V-FITC/PI assay, WB analysis	200 μM, 10–50 *μ*g/mL	Ethanol extract (Noh et al., 2018)
4	Guanyinlian	*Dioscorea zingiberensis* C. H. Wright	*In vivo*	MCAO/R model	5–50 mg/kg	Deltonin (Zhang Y. et al., 2020) Total steroidal saponin, Steroidal saponins (Zhang et al., 2018)
5	Baojiaolian	*Dysosma versipellis* (Hance) M. Cheng	*In vitro*	SH-SY5Y human neuroblastoma cells	1–40 *µ*M	Flavonoid metabolites (Sun et al., 2022)
6	Mohanlian	*Eclipta prostrata* (L.) L	*In vivo*	Caesarean-derived rats’ model, PTZ kindling model	5–20 mg/kg, 50–200 mg/kg/day	Ehanol extract, Butanol fraction of methanol extract, Eclalbasaponin II, Luteolin, Wedeloactone (Feng et al., 2019)
7	Yinxianlian	*Goodyera schlechtendaliana* Rchb. f	*In vivo*	HIST, PTZ seizure test	25–50 mg/kg	Flavonoid glycoside (Du et al., 2008); Goodyschle A (Dai et al., 2021); Goodyerin (Du et al., 2002)
8	Qiyelian	*Gynostemma pentaphyllum* (Thunb.) Makino	*In vitro/In vivo*	Lipopolysaccharide-induced anxiety/depression model, Biochemical analysis, Rat hippocampal synaptosomes, KA-induced injury model	10 μM, 1.0 mg/kg, 50–200 mg/kg/day	Gypenosides (Guo M. et al., 2024); Ombuoside (Wu et al., 2024d); Gypenoside XVII (Lu et al., 2024)
9	Honghanlian	*Hypericum ascyron* L	*In vitro*	SH-SY5Y human neuroblastoma cells, Neurotoxin-induced injury model, DCFH-DA assay, Hoechst 33,342 staining, WB analysis, P19 embryonic carcinoma cells	10–40 μM, 10 *μ*g/mL	Rearranged acylphloroglucinol derivatives (Wang S. et al., 2024); Hyperascyrins (Zhen et al., 2019); Ethyl acetate fraction (Wang et al., 2018)
10	Dabanbianlian	*Lobelia davidii* Franch	*In vitro*	PC12 cells	40 *μ*M	Polyacetylene metabolites, alkaloids, and lignin metabolites (Juelin, 2024)
11	Siyelian	*Marsilea quadrifolia* L	*In vivo*	Lithium-pilocarpine model, MSG model	25–400 mg/kg/day	1-triacontanol cerotate (Snehunsu et al., 2015); Methanol extract (Bhadra et al., 2012); Hydroalcoholic extract (Subramanian et al., 2023)
12	Jiujielian	*Peristrophe japonica* (Thunb.) Bremek	*In vitro/In* *vivo*	BV-2 microglial cells, 5xFAD transgenic mice model	1.25–20 μM, 10–20 mg/kg	Ciliatoside A (Guo et al., 2025)
13	Dujiaolian	*Pinellia pedatisecta* Schott	*In vitro*	PC-12 Model, MTT assay	1–50 *µ*M	Alkaloids (Chen et al., 2025b); Norsesquiterpenes (Chen et al., 2025a); Diketopiperazine and purine alkaloids (Chen et al., 2025c)
14	Dujiaolian	*Sauromatum giganteum* (Engl.) Cusimano & Hett	*In vitro/In vivo*	HEK293T/BK channel expression model, Acute brain slices, 4-VO model	1–20 mg/kg	Total alkaloid extract (Chi et al., 2010)
15	Banzhilian	*Scutellaria barbata* D. Don	*In vitro/In vivo*	CUMS, Cell-based neuroprotection assay, MTT assay, WB analysis	1–50 μM, 5–20 mg/kg/day	Hispidulin (Pannu et al., 2024)
16	Bixuelian	*Stephania japonica* (Thunb.) Miers	*In vitro/In vivo*	SH-SY5Y human neuroblastoma cells, Neurotoxin-induced injury model, MTT assay, Oxidative stress, WB analysis, Annexin V-FITC/PI assay, JC-1 staining, PC12 cells, Hoechst 33,342 staining, Scopolamine memory impairment model, AChE inhibition assay, DPPH	0.50–10 μM, 1–100 *μ*g/mL, 100–400 mg/kg/day	Phenolic metabolites, Flavonoids (Hijam et al., 2024); Alkaloid (Xiao et al., 2019); chloroform fraction (Al-Amin et al., 2022); Stepharine (Hao et al., 2020)
17	Jingoulian	*Uncaria sinensis* (Oliv.) Havil	*In vitro/In vivo*	LPS-activated microglia model, tMCAO, Glutamate-induced excitotoxicity model (in primary neurons), PTI model	0.10–10 μM, 1–100 *μ*g/mL, 200 mg/kg/day	1-Methoxyoctadecan-1-ol (Jang et al., 2014); Hexane extracts (Kang et al., 2015)

### 3.11 Other effects

Sauchinone in Baihelian (*Saururus chinensis* (Lour.) Baill.) also exhibits notable pharmacological properties (Shi et al., 2019). Xiang et al. (2011) also found that the extract and metabolite emodin-8-O-*β*-D-glucoside (E.G.,) from Daosilian (*Bistorta amplexicaulis subsp. Sinensis* (F. B. Forbes & Hemsl. ex Steward) Soják) directly stimulates the proliferation and differentiation of osteoblasts. The purified lectin from Dengtailian (*Arisaema erubescens* (Wall.) Schott) agglutinates red blood cells of rabbits (Liu et al., 2011), mice, and dogs but not from chickens and humans. The flavonoid metabolite Schaftoside in Dengtailian inhibits the increase in melanin production in B16F1 cells stimulated by α-melanocyte-stimulating hormone. It also downregulates the expression of tyrosinase (TYR) and tyrosinase-related protein 1 (TRP1) and inhibits melanin production by activating autophagy in melanocytes (Kim et al., 2018). In the drug-induced liver injury (DILI) mouse model (Jiang et al., 2021), Gouyabanzhilian (*Sedum sarmentosum* Bunge) reduces inflammation and regulates the Nrf2-ARE cascade. It not only supports normal liver cell growth and inhibits APAP-induced liver cell apoptosis but also suppresses the expression of Nrf2-ARE proteins in the liver tissue of the DILI mouse model. This suggests that regulating the Nrf2 signaling pathway helps the active metabolites in Gouyabanzhilian prevent drug-induced liver injury. Zhou J et al. (Zhou et al., 2020) found that Guanyinlian (*Dioscorea zingiberensis* C. H. Wright) can significantly reverse the destruction of the blood-testis barrier (BTB), testicular tissue damage, and abnormal sperm morphology in diabetic mice, and protect against diabetes-induced testicular damage. Du et al. (Du et al., 2017) isolated a diarylheptane from *D. zingiberensis* C. H. Wright and observed that its anti-pancreatitis activity against taurocholic acid sodium salt hydrate (NaT)-induced pancreatic acinar necrosis was superior to that of caffeine. Research shows that Jizhualian (*Sceptridium ternatum* (Thunb.) Lyon) extract has a significant therapeutic effect on radiation-induced pulmonary fibrosis (RIPF) *in vitro* and *in vivo*, suggesting that it may exert anti-RIPF effects by regulating the EGFR/p38-MAPK/NF-κB/CEACAM1 signaling pathway (Zhang Y. et al., 2025). It may also exert beneficial effects against pulmonary fibrosis by targeting the SETDB1/STAT3/p-STAT3 pathway, indicating its therapeutic potential (Zou et al., 2023). When administered at 1.4 g/kg for 12 weeks, Mohanlian (*Eclipta prostrata* L.) botanical drug extract (Feng et al., 2019) effectively increased bone mass and suppressed weight gain in ovariectomized rats, with no adverse effects observed. This outcome is attributed to dual inhibition of bone formation and resorption, with resorption being more prominent, possibly involving downregulation of the key osteoclast factor RANKL. Shen et al. (Shen et al., 2024) found that the total glycosides of *Gynostemma pentaphyllum* in Qiyelian (*G. pentaphyllum* (Thunb.) Makino) significantly increased the expression of key proteins in the LOX1-PI3K-AKT-eNOS pathway, thereby improving hyperlipidemia. Leonurine in Yimulian (*Leonurus japonicus* Houtt.) prevents Ang II-induced cardiac remodeling and dysfunction by inhibiting the MAPK/NF-κB pathway (Shen et al., 2023). It also alleviates acute ischemic kidney injury by activating Nrf2 to counteract oxidative stress and inhibiting TLR4/NF-κB-mediated inflammatory gene expression (Han et al., 2022).

## 4 Toxicity

Research indicates that certain “Lian” drugs have toxic effects. “Bixuelian” (*Aristolochia tubiflora* Dunn) and “Zhushalian” (*Aristolochia tuberosa* C. F. Liang and S. M. Hwang) are species in the *Aristolochia* genus. These plants contain aristolochic acid, which is nephrotoxic and can cause acute renal failure in rats (Bellamri et al., 2025). “Dengtailian” (*Arisaema heterophyllum* Blume, *A. erubescens* (Wall.) Schott, and *Arisaema amurense* Maxim.) and “Dujiaolian” (*Pinellia pedatisecta* Schott) belong to the Araceae family. Plants in this family often exhibit significant irritant toxicity, with symptoms such as lip pain and swelling, sore throat, voice loss, drooling, tracheal obstruction, breathing difficulties, and, in severe cases, suffocation and death. Skin contact can lead to itching, eczema, and, in severe cases, contact dermatitis (Na et al., 2021). Calcium oxalate needle crystals, mainly composed of calcium oxalate, proteins, and sugars, are considered one of the primary irritants in this family. Although Chuanxinlian (*Aconitum sinomontanum* Nakai) (Chunyan et al., 2022) does not contain the highly toxic aconitine, it is a TCM with significant toxicity and high efficacy. Improper use can easily cause harmful reactions, as its alkaloids mainly affect the muscles, the central nervous system, and the heart. Research shows that podophyllotoxin from Bajiaolian (*Dysosma versipellis* M. Cheng) remodels the gut microbiome, shifting its metabolic output. This disruption affects metabolic pathways relevant to the heart, including amino acid, nucleotide, and steroid hormone metabolism, ultimately resulting in energy imbalance, apoptosis, and oxidative stress, which can cause cardiotoxicity (Sun et al., 2025). Studies on *S. chinensis* (Lour.) have identified toxic metabolites, including volatile oils such as elemene and nutmeg ether. However, it remains unclear whether these are the active toxic metabolites of *S. chinensis* (Lour.) (Zhen-hua et al., 2013).

In traditional medicine, Tujia pharmacists have long recognized the toxicity of drugs. On the one hand, they use processing and pharmaceutical methods to reduce or remove toxicity. For example, Wudou (*Aconitum carmichaelii* Debeaux) is toxic, and pharmacists use boiling to mitigate its toxicity. MAOhouzi (*Dioscorea bulbifera* L.) has negligible toxicity, and pharmacists use a urine treatment method (soak the medicinal urine overnight, remove, and dry it) to reduce its toxicity. On the other hand, by utilizing the properties of drugs and using compatibility methods to restrict toxicity, for example, siliangre has a hot nature, spicy taste, and negligible toxicity. When treating symptoms such as wind-cold, headache, and phlegm, pharmacists use Yanchuanxiong (cold-natured, slightly bitter taste) in addition to Yejuhua. Because Yejuhua has a cold nature, a bitter taste, and a detoxifying effect, it helps balance the toxicity of the Siliangre. It is combined as a remedy for dispelling wind-cold and relieving pain. The three aspects are that Tujia pharmacists have summarized the anti-fear laws of medicine in a large number of medical practices, such as the fourteen anti, thirteen anti, thirty anti, etc. These patterns have significant implications for clinical medication (Yang, 2007).

## 5 Summary of chemical composition

Reviewing the above ten practical biological activities, we can get the following key bioactive metabolites. Flavonoids (quercetin, luteolin, apigenin, scutellarin) play a broad “multi-target supporting role”, participating in almost all listed activities, especially antioxidant, anti-inflammatory, antiviral (inhibition of entry/replication enzymes), liver protection, and anti-diabetes. They also show excellent antioxidant and anti-inflammatory activities, and are suitable for early prevention of chronic diseases as dietary supplements. They can weakly regulate a large number of kinases and transcription factors (such as PI3K/Akt, NF-κB, Nrf2), making them suitable for multifactorial diseases. However, oral bioavailability is often less than 2%, and the plasma concentration of the prototype metabolite is exceptionally low (nM), far below the effective concentration *in vitro* (*μ*M). Tens of thousands of papers have been published on quercetin and other flavonoids, but clinical translation has been almost non-existent; many studies are low-level replicates. Overall, it is an excellent model molecule for understanding plant chemical defense and cell signaling networks. However, as a single therapeutic drug, development has entered a dead end. Future research should focus on the *in vivo* activity of its actual metabolites or on its use as an adjuvant therapy/sensitizer. Saponin metabolites (dioscin, gypenosides, aescin/escin, etc.) exhibit therapeutic effects and show apparent dose-dependent responses in various disease models. They can simultaneously intervene in multiple pathological core processes, such as oxidation, inflammation, and apoptosis; however, their oral bioavailability is extremely low (often <5%) due to their large molecular weight and high polarity, which makes them difficult to penetrate the intestinal mucosa and blood-brain barrier. Their membrane activity leads to hemolysis, a common risk, and many of their protective effects may stem from systemic anti-inflammatory/antioxidant effects rather than specific effects on target organs (such as the brain). Overall, they are excellent pharmacological tool molecules and lead metabolites. Still, they cannot become successful oral or central nervous system drugs unless they are thoroughly modified using advanced delivery systems (nano targeting). Alkaloids such as lappaconitine, cephalothin, and podophyllotoxin often have clear and potent molecular targets. They not only have good lipid solubility and can effectively penetrate the blood-brain barrier, acting on the central nervous system, but also act on specific ion channels (Na^+^, Ca^2+^), enzymes (topoisomerases), or receptors (opioid receptors), producing rapid and potent effects; However, their effective dose is very close to the toxic dose, and cardiac toxicity, neurotoxicity, and addiction are common problems. They act on conserved targets (sodium channels), making them challenging to distinguish physiological from pathological states, and have significant side effects. Overall, their development must undergo rigorous toxicological screening and rational structural modification to separate efficacy and toxicity. Directly using the original natural alkaloids as drugs is dangerous and impractical. For other active metabolites, their activities span various neighborhoods and act on different targets. Many extracts have good qualitative effects on a particular activity. Before determining the specific active metabolites, we cannot deny that crude extracts are multi-metabolite effective. In future research directions, it is necessary to first clarify the transition from crude extracts to precise active metabolites.

## 6 Discussion

The vast majority of current research remains at the superficial description of “a certain extract/metabolites have different activities,” for example, flavonoids (quercetin) show weak inhibition across almost all pathways (NF-κB, PI3K/Akt, MAPK), but is this due to non-specific binding to multiple proteins or a cascade effect triggered by an upstream main target (kinase or receptor)? Based on high-concentration (10–100 *μ*M) *in vitro* cell experiment data, efficacy was hastily extrapolated to an *in vivo* setting. This completely ignores the constraints of pharmacokinetics (ADME). For example, flavonoids and saponins generally have low oral bioavailability (<5%), poor blood-brain barrier penetration, intense first-pass metabolism, and high plasma protein binding, resulting in *vivo* effective concentrations that are much lower than their *in vitro* EC_50_. Overuse of various pharmacological activity models, such as antibacterial, relies on broth dilution of standard strains, ignoring the effects of biofilm, retained bacteria, the *in vivo* microenvironment, and host immunity; anti-tumor, relies on 2D cultured cancer cell lines and subcutaneous transplant tumor models, which are severely disconnected from the tumor microenvironment, heterogeneity, and the metastasis process; neuroprotection/antidiabetic, relies on acute injury models (CCl_4_ hepatotoxicity, scopolamine-induced memory impairment), which cannot simulate the actual pathology of human chronic, progressive, multifactorial diseases (alzheimer’s disease, type-2 diabetes). The application of advanced models that better simulate the complexity of human diseases, such as organoids, patient-derived xenograft models, genetically modified disease models, and microbiota-host co-culture systems, is seriously insufficient. Only positive results are reported, with cytotoxicity, hemolytic activity, side effects on normal cells/tissues, and selectivity index calculations and discussions. Mechanism research is highly homogeneous and superficial; for example, DPPH/ABTS is used to measure antioxidant activity, TNF-*α*/IL-6 to measure anti-inflammatory activity, and Caspase-3 is measured to assess apoptosis. These are outcome indicators rather than mechanisms. There is a lack of comprehensive assessment of acute toxicity, sub-chronic toxicity, reproductive toxicity, and mutagenicity. For diseases that have been treated for a long time (diabetes and neurodegenerative disease), long-term toxicity data are almost zero. More than 90% of active metabolites fail in animal experiments, not only because some new metabolites lack pharmacological activity, but also because subsequent verification of activity is delayed. For future research work, it is recommended to use unbiased techniques such as thermal proteomics analysis (TPP), drug affinity reaction target stability (DARTS), or light affinity labeling combined with proteomics to search for binding targets at the whole proteome level, and to verify the functional necessity of the targets using methods such as target gene knockout/knockdown cells, specific inhibitors, and site-directed mutations. Research on antibacterial and anti-tumor effects must undergo model upgrades, such as biofilm models, macrophage sterilization models, animal infection models, patient-derived organoids (PDO), patient-derived xenograft models (PDX), and genetically engineered mouse models (GEMM), to better evaluate their activity ranges. According to current research, the standardization levels of raw medicinal plants and their extracts are low, and there is no specialized quality control, resulting in the inability to quantify the related research. The research path is narrow, and the depth is low. Establishing a standardized extraction process for core species is the primary measure for Tujia medicine research. Stop reporting low-value crude extract activity; research must advance to the separation, identification, and confirmation of active monomers, and establish quality standards based on activity/chemical markers. Currently, the field of pharmacological research on natural products is at a crossroads. There is a “broad path” to continue producing massive amounts of low-conversion-value papers, and another “narrow path” to a few truly innovative therapies that are full of challenges. Choosing the “narrow path” means embracing strict scientific standards, pursuing the depth of mechanisms rather than breadth, focusing on the real efficacy of drugs *in vivo* rather than external illusions, and bravely advancing the lengthy and expensive clinical translation process.

## 7 Conclusion

The identities of certain Tujia ethnomedicines, especially those within the same family but from different species, need further confirmation. Meanwhile, many “Lian” drugs remain unexplored, showing significant research and development potential in this field. In this narrative review, we found that pharmacological research does not fully support the effectiveness of many Tujia ethnic medicines. Therefore, it is essential to emphasize the ethnic traits of Tujia medicine, guided by Tujia medical theory and clinical experience. Furthermore, recent studies have identified new pharmacological effects for some known metabolites, including mangiferin, dioscin, quercetin, quercitrin, and oleanolic acid. This highlights the need for an in-depth investigation into the emerging pharmacological roles of key metabolites in Tujia ethnic medicines, with Tujia medical practices serving as a guide for further pharmacological research.

## Glossary

AAW model: Acetic acid-induced mouse writhing model
ACC-M cells: Adenoid cystic carcinoma cells
AD mouse models: Alzheimer’s disease mouse models
AE: Acute endometritis
AFB1 inhibition assay: Aflatoxin B1 production inhibition assay in yeast extract sucrose broth
AIA model: Adjuvant-induced arthritis model
AIRAP Model: Ammonia-induced rat acute pharyngitis model
ALI: Acute lung injury
Annexin V-FITC/PI assay: Flow cytometric analysis of apoptosis using annexin double staining
AP: Acute pancreatitis model
ApoE−/− + HFD model: Apolipoprotein E-deficient mouse model of high-fat diet-induced atherosclerosis
APP/PS1 mice: Transgenic mouse model of alzheimer’s disease
ARDS: Acute respiratory distress syndrome
PC-12 Model: Amyloid peptide-induced neurotoxicity model
ATDC5 cells: Mouse embryonic tumor cells
BBB: Blood-brain barrier
BCCAO: Bilateral common carotid artery occlusion
BchE: Butyrylcholinesterase
BCSC model: Breast cancer Stem cell Model
BU-HPLC/MS: Bio-affinity ultrafiltration coupled with high-performance liquid chromatography/Mass spectrometry
Calu-3 cells: SARS-CoV-2-infected human lung epithelial cells
CCK-8 assay: Cell counting Kit-8 assay
CCI Model: Chronic constriction injury of the sciatic nerve
**CD4** ^**+**^ **T cells**: Cluster of differentiation 4-positive T lymphocytes
CDX: Cell line-derived xenograft
*C. elegans*: *Caenorhabditis elegans*
CER-AP model: Caerulein-induced acute pancreatitis model
CFA model: Complete freund’s adjuvant-induced Inflammatory pain model
CIA-CNP model: Carrageenan-induced chronic non-bacterial prostatitis model
CIA model: *λ*-Carrageenan-induced acute paw edema model
CIA Model (Collagen): Collagen-induced arthritis model
CLSM: Confocal laser scanning microscopy
Crystal violet biofilm assay: Crystal violet staining assay for biofilm formation inhibition
CUMS: Chronic unpredictable mild stress
Cup-plate method: Oxford cup/Cylinder method
db/db mice: Spontaneous type-2 diabetic mice
DCFH-DA assay: Reactive oxygen species detection assay using 2′,7′-dichlorodihydrofluorescein diacetate
D-gal aging model: D-galactose-induced aging model
D-GalN model: D-Galactosamine-induced acute liver injury model
DLA Ascites Model: Dalton’s lymphoma ascites model
DMM: Destabilization of the medial meniscus
DOX cardiotoxicity model: Doxorubicin-induced cardiotoxicity/Cardiomyopathy model
DPPH: 1,1-Diphenyl-2-picrylhydrazyl
DSS cycle model: Dextran sulfate sodium-induced chronic colitis model
DSS model: Dextran sulfate sodium-induced colitis model
EMT: Epithelial-mesenchymal transition
FM: Fluorescence microscopy
FMLP: *N*-Formylmethionyl-leucyl-phenylalanine
GO: Graves’ orbitopathy
GTPase assay: Guanosine triphosphatase assay
HaCaT cells: Human adult low calcium high temperature keratinocyte cell line
HBT: Human bait technique
H460 cells: NCI-H460 human large cell lung carcinoma cells
HCT-116 cells: HCT-116 human colorectal carcinoma cells
HDFs: Human dermal fibroblasts
HEECs: Human endometrial epithelial cells
HepG2 cells: Human hepatocellular carcinoma (HCC) HepG2 cells
HGC-27 cells: HGC-27 human gastric carcinoma cells
HIST: Hexobarbital-induced sleeping time test
HNE activity inhibition assay: Human neutrophil elastase enzymatic inhibition assay
**H**_**2**_ **O**_**2**_ **-PC12**: H_2_O_2_-induced PC12 cell injury model
HPLC: High-performance liquid chromatography
HPT: Hot-plate test
*H. pylori*-infected AGS cells: *Helicobacter pylori* co-culture model with gastric epithelial cells
*H. Pylori* model: Mouse/Rat model of *Helicobacter pylori* infection and gastritis
HR-GIP: High-resolution growth inhibition profiling
HRMCs: Human renal mesangial cells
H/R model: Hypoxia/Reoxygenation-induced injury model
HUVEC: Human umbilical vein endothelial cells
IAV-ALI model: Influenza A virus-induced acute lung injury model
IFA: Immunofluorescence Assay
IL-1β: Interleukin-1*β*
I/R Model: Cerebral ischemia/Reperfusion injury model
ISO model: Isoproterenol-induced cardiac hypertrophy model
Kirby-Bauer test: Kirby-bauer disk diffusion test
L-Arg SAP model: L-Arginine-induced severe acute pancreatitis model
LORA: Low oxygen recovery assay
LPS-AKI model: Lipopolysaccharide-induced acute kidney injury model
LPS-induced sepsis model: Lipopolysaccharide-induced sepsis model
LPS model: Lipopolysaccharide-induced systemic inflammation/Septic shock model
MABA: Microplate alamar blue assay
MBC assay: Broth dilution method for minimum bactericidal concentration determination
MCAO/R model: Middle cerebral artery occlusion and reperfusion model
MCF-7 cells: MCF-7 human breast adenocarcinoma cells
MC903 model: MC903 (calcipotriol)-induced atopic dermatitis model
MDA-MB-231 cells: MDA-MB-231 human triple-negative breast adenocarcinoma cells
MFC assay: Broth dilution method for minimum fungicidal concentration determination
MIC assay: Broth dilution method for minimum inhibitory concentration determination
MI/R model: Myocardial ischemia/reperfusion injury model
MKN-45 cells: MKN-45 human gastric adenocarcinoma cells
MPC-83 cells: Mouse pancreatic acinar cell line
MSSA: Methicillin-sensitive *Staphylococcus aureus*
MSU crystal model: Monosodium urate crystal-induced acute gouty arthritis model
MTT assay: 3-(4,5-Dimethylthiazol-2-yl)-2,5-diphenyltetrazolium bromide assay
NaT-SAP-ALI model: Sodium taurocholate-induced severe acute pancreatitis-associated acute lung injury model
NHDF cells: Normal human dermal fibroblasts
NOD/SCID: Non-obese diabetic/severe combined immunodeficiency
NRCFs: Neonatal rat cardiac fibroblasts
NRK-52E cells: the NRK-52E rat renal tubular epithelial cell line
NSAID ulcer model: Indomethacin-induced gastric ulcer model
OA chondrocytes: Osteoarthritis chondrocytes
OFs: Primary human orbital fibroblasts
OGD/R: Oxygen-glucose deprivation/reperfusion
OVA model: Ovalbumin sensitization and challenge model
OVX mice: Ovariectomized mouse model of postmenopausal osteoporosis
OXA model: Oxazolone-induced allergic contact dermatitis model
PBMCs: peripheral blood mononuclear cells
PI flow cytometry: Flow cytometric analysis with propidium iodide (PI) staining
PM (SRM 2786) model: Standard reference material 2786 fine particulate matter intranasal challenge model
PO/HX model: Potassium oxonate and hypoxanthine-induced hyperuricemia model
PO model: Potassium oxonate-induced hyperuricemia model
qRT-PCR: Quantitative reverse transcription polymerase chain reaction
QTOF-MS/MS: Quadrupole time-of-flight tandem mass spectrometry analysis
ROS detection: Reactive oxygen species detection
RTCA: Real-time cell analysis
SAP: Severe acute pancreatitis model
Scopolamine model: Scopolamine-induced memory impairment/cognitive deficit model
SEM: Scanning electron microscopy
SiHa cells: SiHa human cervical cancer cell line
SMMC-7721 cells: SMMC-7721 human hepatocellular carcinoma cells
Spike PsV: SARS-CoV-2 Spike protein-pseudo-typed lentivirus
Spore germination assay: Spore germination inhibition assay in liquid culture
STZ mice: Streptozotocin-induced type-1 diabetes mellitus model
SYTOX Green assay: Bacterial membrane permeability/integrity assay
TEM: Transmission electron microscopy
TFL: Tail-flick latency
THP-1: Tohoku hospital pediatrics-1
Time-kill assay: Time-kill kinetic assay
tMCAO model: Transient middle cerebral artery occlusion-induced focal cerebral ischemia model
TPA model: 12-O-Tetradecanoylphorbol-13-acetate-induced inflammation model
TUNEL assay: Terminal deoxynucleotidyl transferase nick-end labeling assay
UC: Ulcerative colitis model
WB analysis: Western blot analysis
WHO Larval Bioassay: World health organization standard larval bioassay
XDR-AB: Extensively drug-resistant *acinetobacter* baumannii
XME model: Xylene-induced mouse ear edema model
XTT assay: 2,3-bis-(2-methoxy-4-nitro-5-sulfophenyl)-2H-tetrazolium-5-carboxanilide reduction assay
