# Supplementary material for: Comprehensive review of tujia “Lian” medicinal botanical drugs: traditional classification system, phytochemical, and pharmacological profile
Source: Front Pharmacol. 2026 Feb 18;17:1747999. doi: 10.3389/fphar.2026.1747999 (PMC12957786; doi:10.3389/fphar.2026.1747999)
Supplement: Supplementary file 1 [file Table1.docx]

**SUPPLEMENTARY TABLE 1 Origin, nature, flavor, and effectiveness of "Lian" medicines [collection of 'Lian' medicine comes from ancient books and monographs** (Fang et al., 2007; Yuan, 2007; Yang et al., 2016a)**].**

| **Sr. No.** | **"Lian" drugs**  **name** | **Common name** | **Origin** | | | **Medicinal effects and uses** |
| --- | --- | --- | --- | --- | --- | --- |
|  |  |  | **Family** | **Species** | **Medicinal parts** |  |
| 1 | Banzhilian | Yashuacao, Bingtoucao, Waitoucao, Tongjingcao, zaishengcao | *Lamiaceae* | *Scutellaria barbata* D. Don | Botanical drug | Cold in nature, with a pungent and slightly bitter flavor. It eliminates toxins, clears heat, promotes qi flow, removes blood stasis, and enhances tissue regeneration. |
| 2 | Xinyebanzhilian | Waercao, Hunbingcao | *Lamiaceae* | *Scutellaria indica* L. | Botanical drug | Cold in nature, with a bitter and slightly spicy flavor. It clears heat and toxins, promotes blood circulation to remove stasis, dispels wind, and relieves pain. |
| 3 | Babaolian | Chouqinjiamu, Dahonghua, Mudanqi, Dahongpao, Yemudan | *Lamiaceae* | *Clerodendrum bungei* Steud. | Stem, leaf, and roots | Warm by nature, with a sweet and spicy flavor. It helps remove toxins and qi, accelerates the blood flow, reduces swelling, and relieves stasis and pain. |
| 4 | Fengkelian | Wuyueliang, Baihuacao, Denglongqiu | *Lamiaceae* | *Prunella asiatica* Nakai | Aerial part | Cold in nature and bitter in flavor. It eliminates toxins, supports liver health, promotes fluid drainage, reduces swelling, disperses nodules, and stimulates muscle growth. |
| 5 | Yimulian | Yuemucao, Xuanfengcao, Silingcao | *Lamiaceae* | *Leonurus japonicus* Houtt. | Botanical drug | Cold in nature, with a bitter and pungent flavor. It promotes blood circulation and regulates the meridians. It helps stimulate the flow of water to relieve swelling and reduce urine pressure, which drives qi. |
| 6 | Hudoulian* | Shandougen, Hudouqi, Wandouqi | *Fabaceae* | *Euchresta tubulosa* Dunn | Roots, leaves, and seeds | Cold in nature, with a bitter and pungent flavor. It clears heat and relieves throat pain. |
| 7 | Bixuelian | Gonglaoshuteng, Yetaocao, Baozhuxiao | *Menispermaceae* | *Stephania japonica* (Thunb.) Miers | Roots and rhizomes | Cold in nature, with a bitter and pungent flavor. It drives wind, activates collaterals, eliminates toxins, clears heat, relieves pain, and reduces swelling. |
| 8 | Baiweilian | Wuguiqi, Xiashangui, Jinguilian, Kujinlian | *Cucurbitaceae* | *Hemsleya chinensis* Cogn. ex F. B. Forbes & Hemsl. and *Hemsleya macrosperma* C. Y. Wu ex C. Y. Wu & C. L. Chen | Roots tuber | Cold in nature and bitter in taste. It stimulates the flow of qi to relieve pain, detoxify, and stop diarrhea. It cools the blood to stop bleeding, reduces swelling, and relieves pain. However, it is slightly poisonous. |
| 9 | Qiyelian | Qiyedan, Xiaokuyao, Qiyeshen | *Cucurbitaceae* | *Gynostemma pentaphyllum* (Thunb.) Makino | Botanical drug | Cold in nature, with a bitter, slightly sweet taste. It helps eliminate toxins, clear heat, reduce phlegm and cough, resist aging, and combat fatigue. |
| 10 | Kugualian* | Kuguaqi, Shankugua | *Cucurbitaceae* | *Trichosanthes cucumeroides* (Ser.) Maxim. | Roots | Cold in nature and bitter in flavor, it aids in detoxification and helps reduce inflammation and swelling. It also helps dispel heat, minimize pain, drive out wind, and relieve itching. Additionally, it neutralizes toxins. However, it is slightly poisonous. |
| 11 | Jixuelian | Tiebanjin, Tiejueji | *Thelypteridaceae* | *Pronephrium penangianum* (Hook.) Holttum | Botanical drug and rhizome | Cold in nature and bitter in taste. It drives qi, relieves pain, promotes blood circulation, and stops bleeding. |
| 12 | Gouyabanzhilian | Gouyacao, Gouyaban | *Crassulaceae* | *Sedum sarmentosum* Bunge | Botanical drug | Cold in nature, with a light and slightly bitter flavor. It clears heat and detoxifies, disperses swelling, and dissipates binds. |
| 13 | Guanyinzuolian | Tongdabusi, Masanqi, Sanqi | *Crassulaceae* | *Phedimus aizoon* (L.) 't Hart | Botanical drug | Neutral in nature and sour in flavor, it aids in breaking and cooling the blood and promotes blood circulation. |
| 14 | Lvdoulian | Candouqi, Huanghuashen | *Crassulaceae* | *Rhodiola yunnanensis* (Franch.) S. H. Fu | Botanical drug | Neutral in nature, it has a slightly bitter and astringent flavor. It dispels wind and dampness, disperses blood stasis, stops bleeding, and reduces swelling. |
| 15 | Shiliancao* | Shanwasong, Duishanhua, Honghuayansong | *Crassulaceae* | *Sinocrassula indica* (Decne.) A. Berger | Botanical drug | Neutral in nature and sour in taste. It aids in detoxification, helps to stop bleeding, and treats dysentery. |
| 16 | Banbianlian | Ximicao, Jijiesuo, Ganjicao, Guarencao, Banbianju, Banbianhua | *Campanulaceae* | *Lobelia chinensis* Lour. | Botanical drug | Neutral in nature, spicy in flavor. It clears heat, detoxifies, promotes diuresis, and reduces swelling. |
| 17 | Dazhongbanbianlian | Dabanbianlian | *Campanulaceae* | *Lobelia sessilifolia* Lamb. | Botanical drug | Neutral in nature, with a flavor that is sweet and spicy. It promotes lung health, reduces phlegm, clears heat and detoxifies, and alleviates swelling. |
| 18 | Dabanbianlian | Shetouhua, Honghuanaijiang, Yeyan | *Campanulaceae* | *Lobelia davidii* Franch. | Botanical drug | Neutral in nature and sweet in flavor. It clears heat, detoxifies, resolves phlegm, quenches thirst, promotes urination, and induces vomiting. It is slightly poisonous. |
| 19 | Huoxuelian | Huluqi, Baihulu | *Asteraceae* | *Adenocaulon himalaicum* Edgew. | Botanical drug | Neutral in nature, astringent, and slightly bitter in flavor. It clears heat, detoxifies, promotes blood circulation, removes stasis, reduces swelling, and relieves pain. |
| 20 | Dahanliancao | Tiankui, Xiquecao | *Asteraceae* | *Bidens tripartita* L. | Botanical drug | Neutral in nature, with a sweet and subtly bitter flavor. It clears heat, detoxifies, nourishes yin, and reduces sweating. |
| 21 | Baidulian* | Mantianxing, Kumaicai, Kudiezi | *Asteraceae* | *Ixeris polycephala* Cass. Ex. DC. | Botanical drug | Cold in nature, with a bitter and pungent flavor. It eliminates toxins, clears heat, relieves pain, and reduces swelling. |
| 22 | Mohanlian | Yekuihua, Hanliancao, Modoucao | *Asteraceae* | *Eclipta prostrata* (L.) L. | Botanical drug | Cold in nature, with a sweet-and-sour flavor. It nourishes Yin and strengthens the kidneys, promotes hemostasis, and relieves pain. |
| 23 | Guanyinlian | NIL | *Marattiaceae* | *Angiopteris evecta* (G. Forst.) Hoffm. | Fresh stem | It is slightly cold in nature and has an astringent flavor. It helps relieve coughing and disperse blood stasis. It is used to treat various forms of tuberculosis and injuries caused by falls and bruises. |
| 24 | Guanyinzuolian* | Xiaoyefengwocao | *Acanthaceae* | *Strobilanthes cyclus* C. B. Clarke ex W. W. Sm. | Botanical drug | Neutral in nature and slightly bitter in flavor. It drives wind, dampness, and detoxifies. |
| 25 | Jiujielian | Jianjingyao, Huatanqing, Xiangkaikou, Jiujieli | *Acanthaceae* | *Peristrophe japonica* (Thunb.) Bremek. | Botanical drug | Cold in nature with a spicy flavor. It drives away wind and fire, reduces phlegm and cough, relieves spasms, and calms the mind. |
| 26 | Luanjiaolian | Yandapao, Guoshangye, Paohuoqi | *Orchidaceae* | *Pholidota yunnanensis* Rolfe | Botanical drug | Cold in nature, with a bitter and slightly numbing flavor. It clears heat and dampness, disperses wind, and relieves pain. It is somewhat toxic. |
| 27 | Guazilian* | Zishangye, Shixiantao, Xiaokouzilan, Genshang | *Orchidaceae* | *Bulbophyllum inconspicuum* Maxim. | Botanical drug | Cold in nature, sweet and light in flavor. It benefits pregnancy, dispels heat, replenishes fluids, supports lung health, and combats tuberculosis. It also moisturizes meridians and relieves pain. |
| 28 | Bancenglian* | Shandouya, Qingtinglan, Hutoujiao | *Orchidaceae* | *Cephalanthera longifolia* (L.) Fritsch | Botanical drug | Cold in nature, slightly sweet, and light in flavor. It clears heat, detoxifies, and moisturizes the lungs. |
| 29 | Yinxianlian | Banyelan, Qisheyao | *Orchidaceae* | *Goodyera schlechtendaliana* Rchb. f. and *Goodyera repens* (L.) R. Br. | Botanical drug | Cold in nature and with a mild flavor, it clears heat and detoxifies, relieves cough, reduces swelling, promotes blood circulation, and alleviates pain. |
| 30 | Daosilian | Jiulongpan, Sanxuedan, Jixueqi, Xuesanqi | *Polygonaceae* | *Bistorta amplexicaulis subsp. Sinensis* (F. B. Forbes & Hemsl. ex Steward) Soják | Botanical drug | It is mildly warm in nature. It has an astringent and somewhat bitter flavor. It relieves blood stasis, stops bleeding, regulates qi, and eases pain. |
| 31 | Huoxuelian | Caoheche, Daosilian, Caoxuejie, Hongzaoxiu | *Polygonaceae* | *Bistorta officinalis* Raf. | Rhizome | It is mildly cold in nature, with a bitter and astringent flavor. It clears heat, detoxifies, cools the blood, disperses lumps, and is somewhat toxic. |
| 32 | Qiaokelian | Jiegusun, Laoshejin, Yunyao, Qiaokelian | *Polygonaceae* | *Persicaria chinensis* (L.) H. Gross | Botanical drug | Cold in nature, with a sour and slightly astringent flavor. It clears heat and detoxifies, relieves dampness and dysentery. It promotes blood circulation and reduces swelling. |
| 33 | Qiaozilian | Honghualian, Huahudie | *Polygonaceae* | *Persicaria runcinata var. sinensis* (Hemsl.) Bo Li | Rhizome and Botanical drug | Cold in nature, sour and astringent in flavor. It clears heat, detoxifies, promotes blood circulation, and relieves pain. |
| 34 | Matilian | Matihuang, Tanggudahuang, Nandahuang | *Polygonaceae* | *Rheum* *palmatum* L. | Rhizome | Cold in nature and bitter in flavor. It cools the fire, soothes the blood, removes blood stasis, unblocks meridians, and helps with food stagnation. |
| 35 | Yanqiaolian | Yanqiaomai | *Polygonaceae* | *Persicaria capitata* (Buch. Ham. ex D. Don) H. Gross | Botanical drug | Warm in nature, bitter in flavor, and spicy. It aids in detoxification, disperses blood stasis, promotes diuresis, and enhances lymphatic circulation. |
| 36 | Xionghuanglian | Zhushaqi, Zhushalian | *Polygonaceae* | *Pleuropterus ciliinervis* Nakai | Roots and tubers | It is slightly cold in nature and sweet in flavor. It clears heat, detoxifies, promotes blood circulation, and relieves pain. It is used to treat injuries sustained during labor and falls. |
| 37 | Shuihuanglian* | Qingyucao, Ganyancao, Danyancao, Qingyedan, Qingjiaodan | *Gentianaceae* | *Swertia angustifolia var. Pulchella* (D. Don) Burkill and *Swertia leducii* Franch. | Botanical drug | Cold in nature and bitter in flavor. It eliminates toxins, clears heat, dispels dampness, and reduces yellowing. |
| 38 | Shuihuanglian | Shuilingzhi | *Gentianaceae* | *Swertia davidii* Franch. | Botanical drug | Cold in nature and bitter in flavor. It clears heat, detoxifies, relieves dampness, and eases pain. |
| 39 | Sanxuelian* | Heihuqi, Jixuelian, Huoxuelian, Meifengcao, Fengweicao | *Pteridaceae* | *Coniogramme japonica* (Thunb.) Diels | Rhizome | It is slightly cool in nature, spicy and slightly bitter in flavor, relieves blood stasis, and treats injuries. It promotes blood circulation, stops bleeding, relaxes tendons, enhances blood flow, dispels wind and dampness, and cools the blood to eliminate stagnation. |
| 40 | Jijiaolian* | Tieganqi, Qinglongpayan, Dayegouyaqi | *Pteridaceae* | *Coniogramme intermedia* Hieron. | Rhizome | Warm in nature, with a sweet and astringent flavor. It effectively relieves rheumatism, strengthens muscles and bones, regulates qi, and improves blood circulation. |
| 41 | Chuanxinlian | Koudaiqi, Pomaohuanyang, Xiongzhangqi | *Ranunculaceae* | *Aconitum sinomontanum* Nakai | Root | Warm in nature, with a bitter and pungent flavor. It helps dispel wind and dampness, promotes blood circulation, relieves pain, and is toxic. |
| 42 | Dayemaweilian | Yandidong, Shisunhuanyang | *Ranunculaceae* | *Thalictrum acutifolium* (Hand. Mazz.) B. Boivin | Botanical drug | Cold in nature and bitter in flavor. It clears heat and detoxifies, dispels wind and dampness, and promotes digestion. |
| 43 | Maweilian | NIL | *Ranunculaceae* | *Thalictrum petaloideum* L. | Roots | Cold in nature and bitter in flavor. It clears heat and detoxifies, supports liver health, enhances vision, and promotes digestion. |
| 44 | Maweilian | Yanguocao | *Ranunculaceae* | *Thalictrum minus var. hypoleucum* (Siebold & Zucc.) Miq. | Root | Cold in nature and bitter in flavor. It clears heat and toxins. It is slightly toxic. |
| 45 | Ruanshuihuanglian* | Ruanganzi, Shuihuanglian, Tuhuanglian | *Ranunculaceae* | *Thalictrum ramosum B.* Boivin | Botanical drug | Cold in nature and bitter in flavor. It clears heat and removes dampness. |
| 46 | Tiexianlian | Wenbidan | *Ranunculaceae* | *Clematis chinensis* Osbeck | Roots and rhizomes | Warm in nature, spicy and salty in flavor. It helps dispel wind and dampness, activates meridians and collaterals, eliminates phlegm and saliva, and removes bone spurs, with slight toxicity. |
| 47 | Yanjielian* | NIL | *Ranunculaceae* | *Dichocarpum dalzielii* (J. R. Drumm. & Hutch.) W. T. Wang & P. K. Hsiao | Roots | Cold in nature, with a pungent and slightly bitter flavor. It reduces swelling and functions as an adaptogen. |
| 48 | Tushanhuanglian | Tiehao, Guijiantui | *Ranunculaceae* | *Thalictrum javanicum* Blume | Botanical drug | Cold in nature and bitter in flavor. It clears heat and toxins. |
| 49 | Xinanyinhualian | Tiecao, Gouniaocao | *Ranunculaceae* | *Anemone davidii* Franch. | Rhizome | Warm in nature, with a bitter and slightly sweet flavor. It promotes blood circulation, relieves pain, detoxifies, and reduces swelling. It is somewhat toxic. |
| 50 | Zhuyetiexianlian | Baibulingxian, Huangyaozi | *Ranunculaceae* | *Clematis terniflora* DC. | Roots | Neutral in nature and bitter in flavor. It cools the blood, reduces fever, detoxifies, and relieves swelling and pain. |
| 51 | Badongmulian* | NIL | *Magnoliaceae* | *Manglietia patungensis* Hu | Flowers | Cold by nature, sweet and astringent in flavor. It relieves blood stasis, eases pain, and also converges and stops bleeding. |
| 52 | Yajiaolian* | Xiaobayuezha, Qiyemutong | *Lardizabalaceae* | *Stauntonia duclouxii* Gagnep. | Rhizome | Warm by nature with a bitter flavor. It regulates qi, tonifies deficiencies, relieves pain, and stops dysentery. |
| 53 | Qiyelian | Suoluoguo | *Sapindaceae* | *Aesculus chinensis* Bunge and *Aesculus chinensis* *var. wilsonii* (Rehder) Turland & N. H. Xia | Seeds | Warm in nature and mildly sweet in flavor. It regulates qi, relieves stomach pain, kills insects, and prevents malaria. |
| 54 | Jixuelian* | Shanqincai, Sanxuecao, Hujiaoxiang | *Apiaceae* | *Sanicula orthacantha* S. Moore | Botanical drug | Warm in nature and bitter in flavor. It clears heat and detoxifies, relaxes tendons, and promotes blood circulation. |
| 55 | Zhenzhulian | Yanpipa, Bingzi, Yanteng | *Moraceae* | *Ficus sarmentosa var. henryi* (King ex Oliv.) Corner | Botanical drug | Fruits are neutral in nature and sweet in flavor. Roots, stems, and leaves are also neutral in nature but have a bitter taste. It helps eliminate wind and dampness, relieve pain, and reduce swelling. |
| 56 | Guanyinlian | Wenwangyizhibi, Jiemuhuaitai, Jixinqi, Bibaoqi, Getengjun | *Balanophoraceae* | *Balanophora involucrata* Hook. f. | Botanical drug | Cold in nature, with a bitter, astringent, and slightly sweet flavor. It cools the blood to stop bleeding, moistens the lungs to alleviate congestion, dispels qi and relieves stagnation, supplements deficiencies, astringes essence, expels fire, and detoxifies. |
| 57 | Guanyinlian | Wenwangyizhibi, Jiemuhuaitai | *Balanophoraceae* | *Balanophora fungosa* J. R. Forst. & G. Forst. | Botanical drug | Neutral in nature, with a sweet and astringent flavor, it moisturizes the lungs, relieves coughs, promotes blood circulation, disperses blood stasis, and relieves pain. |
| 58 | Guanyinlian | Huangjiang | *Dioscoreaceae* | *Dioscorea zingiberensis* C. H. Wright | Rhizome | Cold in nature, with a sweet and bitter flavor. It clears the lungs, relieves cough, detoxifies, reduces swelling, regulates qi, and alleviates pain. |
| 59 | Yeshulian | NIL | *Dioscoreaceae* | *Dioscorea polystachya* Turcz. | Roots tuber | Neutral in nature, sweet and juicy in flavor, it supports qi and fluids. Indications include spleen deficiency, prolonged diarrhea, white chancre buildup, and dry cough. |
| 60 | Niuxuelian | Zhushalian, Xuehulu, Zhushaqi, Hongyaozi, Xueshalian | *Dioscoreaceae* | *Dioscorea cirrhosa* Lour. | Roots tuber | Neutral in nature, with a flavor that is sweet and slightly sour. It regulates qi, promotes blood circulation, stops bleeding, and relieves pain. |
| 61 | Runxuelian | Feijingcao, Luxiancao | *Ericaceae* | *Pyrola calliantha* Andres | Botanical drug | Warm in nature, it has a bitter and slightly astringent flavor. It moisturizes the lungs, relieves coughs, dispels colds, and soothes a sore throat. Additionally, it heals bones, promotes blood circulation, stops bleeding, and dispels wind and dampness. |
| 62 | Jizhualian | Yiduoyun, Huajue, Jinjiduli | *Ophioglossaceae* | *Sceptridium ternatum* (Thunb.) Lyon | Botanical drug | Neutral in nature and sweet in flavor. It helps supplement deficiencies and relieve coughs. It treats dizziness, eliminates phlegm, and aids in the treatment of tuberculosis. |
| 63 | Baihelian | Baijieou, Baiyelian, Weilaoxianbaitou | *Saururaceae* | *Saururus chinensis* (Lour.) Baill. | Rhizome and Botanical drug | It is slightly cold in nature and sweet in flavor. It clears dampness and promotes convergence. Indications include: dizziness, headache, swelling, white discharge, and nocturnal emissions. |
| 64 | Zhuyelian* | Yanghuoqi, Baijiegudan | *Commelinaceae* | *Pollia japonica* Thunb. | Rhizome and Botanical drug | Neutral in nature and sweet in flavor, it supports qi and fluids. Indications include: windiness, dizziness, and lower back pain. |
| 65 | Huoxuelian* | Guanyinlian | *Polypodiaceae* | *Microsorum insigne* (Blume) Copel. | Botanical drug | Cold in nature and light in flavor. It clears heat and detoxifies, functions as a diuretic, promotes blood circulation, and removes stasis. |
| 66 | Mahuanglian* | Fengweisoushanhu, Gunlongcao, Doujiangcao | *Polypodiaceae* | *Selliguea mairei* (Brause) Christenh. | Rhizome | Warm in nature, with a spicy and sweet flavor. It helps dispel wind and cold, relieves pain, and detoxifies. |
| 67 | Baoshilian* | Yubiejinxing, Guamihuanyang, Baoshulian | *Polypodiaceae* | *Lemmaphyllum drymoglossoides* (Baker) Ching and *Lemmaphyllum diversum* (Rosenst.) De Vol & C. M. Kuo | Botanical drug | Neutral in nature and light in flavor. It clears heat, detoxifies, relieves cough, and stops bleeding. |
| 68 | Dengtailian | Shebaogu, Lvnanxing, Tiannanxing, Bainanxing, Banbiansan | *Araceae* | *Arisaema heterophyllum* Blume*, Arisaema erubescens* (Wall.) Schott and *Arisaema amurense* Maxim. | Stem tuber | Warm in nature, with a bitter and pungent flavor. It dispels wind, relieves convulsions, dries dampness, resolves phlegm, reduces swelling, and disperses nodules. However, it is toxic. |
| 69 | Dujiaolian | Gouzhuabanxia, Huzhang, Dasanbutiao | *Araceae* | *Pinellia pedatisecta* Schott | Stem tuber | Neutral in nature, spicy in flavor. It helps reduce swelling and detoxifies. However, it is toxic. |
| 70 | Dujiaolian | Baifuzi | *Araceae* | *Sauromatum giganteum* (Engl.) Cusimano & Hett. | Botanical drug | Warm in nature, with a spicy and sweet flavor. It dispels wind and phlegm, as well as cold and dampness, and relieves spasms and pain. However, it is highly toxic. |
| 71 | Leigonglian | Shinanteng, Qingzhubiao, Daruanjinteng | *Araceae* | *Amydrium sinense* (Engl.) H. Li | Aerial parts and the root | Cold in nature and spicy in flavor. It helps eliminate toxins and qi, disperse blood stasis, and reduce swelling. Nevertheless, it is toxic. |
| 72 | Daoshenglian | Shulingzhi, Dibanzhi, Shuerjue | *Aspleniaceae* | *Asplenium prolongatum* Hook*.* | Botanical drug | Cold in nature and bitter in flavor, it clears heat, detoxifies, promotes blood circulation, dispels blood stasis, and relieves cough and phlegm. |
| 73 | Bajiaolian | Duyeyizhihua, Heyelian, Yexiahua | *Berberidaceae* | *Dysosma versipellis* (Hance) M. Cheng | Rhizome | Cold in nature, with a spicy and bitter flavor. It relieves internal heat, disperses nodules, promotes blood flow, and reduces swelling. It is slightly toxic. |
| 74 | Shuibajiaolian | Jinbianqi, Bajiaowu, Chuanbajiaolian, Bajiaojinpan | *Berberidaceae* | *Dysosma delavayi* (Franch.) Hu | Stem tuber | Cold in nature and bitter in flavor, it promotes blood flow, eliminates toxins, and dispels blood stasis. However, it is toxic. |
| 75 | Bagualian | Baofulian | *Berberidaceae* | *Sinopodophyllum hexandrum* (Royle) T. S. Ying | Roots | Cold in nature, bitter and astringent in flavor. It promotes blood circulation, disperses stasis, relieves pain, and thereby promotes the flow of qi. |
| 76 | Dujiaolian* | Baofuqi, Huangbaofu | *Berberidaceae* | *Dysosma difformis* (Hemsl. & E. H. Wilson) T. H. Wang | Rhizome | Cold in nature, with a bitter and pungent flavor. It clears heat, detoxifies, promotes blood circulation, disperses stasis, reduces swelling, and relieves pain. It is slightly toxic. |
| 77 | Shuifulian | Shuihulu, Fengyanlian | *Pontederiaceae* | *Pontederia crassipes* Mart. | Botanical drugs and roots | It is slightly cold in nature, with a pungent and somewhat astringent flavor. It clears heat, detoxifies, and dehumidifies. |
| 78 | Baierlian | Tiandong, Xiaosanbaibang, Erduomuku | *Asparagaceae* | *Asparagus cochinchinensis* (Lour.) Merr. | Roots tuber | It has a cold nature, with a sweet, slightly bitter flavor. It helps moisturize the lungs to relieve coughing, reduce fever to quench thirst, regulate menstruation to aid in conception, and nourish deficiencies to generate essence. |
| 79 | Baierlian | NIL | *Asparagaceae* | *Asparagus densiflorus* (Kunth) Jessop | Roots tuber | It is slightly cold in nature, with a sweet, somewhat bitter flavor. It nourishes the lungs and fluids. Treats tuberculosis, cough, and infertility. |
| 80 | Dierlian | NIL | *Asparagaceae* | *Asparagus filicinus* D. Don | Roots tuber | Cold in nature, sweet with a slight bitterness. It relieves coughing and nourishes the lungs. It is used for conditions like infertility, tuberculosis, and hemoptysis. |
| 81 | Qiyelian | Tiedengtai, Qiyeyizhihua, Dujiaolian, Zaoxiu | *Melanthiaceae* | *Paris polyphylla var. Chinensis* (Franch.) Hara | Rhizome | Cold in nature, spicy and bitter in flavor. It expels fire, detoxifies, promotes blood circulation, heals injuries, calms the wind, and stops spasms. It is slightly toxic. |
| 82 | Huanghuadaoshuilian* | Jihezishu, Tuhuangqi, Daoshulian, Shushen | *Polygalaceae* | *Polygala arillata* Buch. Ham. ex D. Don | Roots | It has a mild, warm nature and a sweet flavor. It clears wind and dampness, tonifies deficiencies, reduces swelling, regulates meridians, promotes blood circulation, and calms the mind. |
| 83 | Shuihuanglian | Yanjiaocao, Chouhujiao, Tumahuang, Choujiecao | *Rutaceae* | *Boenninghausenia albiflora* (Hook.) Reichb. ex Meisn. | Botanical drug | Warm in nature, sour and bitter in flavor. It relieves blood stasis, intercepts malaria, and alleviates pain. |
| 84 | Wupaolian | Laowupao, Wulongbaiwei, Daoshuilian, Daoshenggen, Wulongguojiang | *Rosaceae* | *Rubus tephrodes* Hance | Roots or tender tips | Neutral in nature, with an astringent, slightly bitter flavor, it helps constrict and stop bleeding. It continues to strengthen muscles and bones, astringes the intestines to prevent diarrhea, firms the bladder, reduces urine, and detoxifies to eliminate toxins. |
| 85 | Shuikulian | Banbianshan, Shuizelan, Yaercao | *Plantaginaceae* | *Veronica anagallis-aquatica* L. | Botanical drug | Cold in nature and bitter in flavor. It dispels dampness, clears heat, alleviates qi and stasis, relieves pain, and stops bleeding. |
| 86 | Siyelian | Siziping | *Marsileaceae* | *Marsilea quadrifolia* L. | Botanical drug | Cold in nature and sweet in flavor. It clears heat, detoxifies, and is also diuretic, reducing swelling. |
| 87 | Honghanlian | Liujinv, Duiyecao, Hunanlianqiao | *Hypericaceae* | *Hypericum ascyron* L. | Aerial part | Cold in nature, with a slightly bitter and astringent flavor. It cools the blood to stop bleeding and clear heat while detoxifying. |
| 88 | Duiyuelian | Duiyecao, Shechakou | *Hypericaceae* | *Hypericum sampsonii* Hance | Botanical drug | Cold in nature and bitter in flavor. It helps eliminate toxins, clear heat, regulate menstruation, and stop bleeding. |
| 89 | Qiaozilian | Hongheierwan, Yanwanzi, Yinyangzi, Hongbaierwan | *Begoniaceae* | *Begonia grandis subsp. sinensis* (A. DC.) Irmsch. | Rhizome and Botanical drug | It has a slightly cold nature and a sour, spicy flavor. It dispels heat to stop diarrhea, moves qi to relieve pain, promotes blood circulation to treat injuries, converges and stops bleeding, and detoxifies to invigorate the throat. |
| 90 | Siyelian* | Sikuaiwa, Hongsikuaiwa, Siyeyizhihua | *Primulaceae* | *Lysimachia paridiformis* Franch. | Botanical drug | Warm in nature, with a spicy, subtly bitter, and slightly sweet flavor. It promotes blood and meridian circulation, stops bleeding, strengthens the collapse, replenishes qi, nourishes blood, and elevates the intestines. |
| 91 | Qikonglian | Zijinzhong, Yangtianzhong | *Melastomataceae* | *Osbeckia stellata* Buch. Ham. ex D. Don | Botanical drug | Cold in nature, sweet and astringent in flavor. It dispels toxins, clears heat, eliminates water retention and dampness, and alleviates coughing. |
| 92 | Bixuelian | Yidianxue, Bixuelei, Tongchenghu, Huangmuxiang, Nanmuxiang, Nanmuxiao | *Aristolochiaceae* | *Aristolochia tubiflora* Dunn | Roots | Slightly cool in nature, spicy and bitter in flavor. It alleviates qi and reduces swelling, eliminates fire, removes dampness, promotes blood circulation, relieves pain, and dispels wind to clear the tendons. It is slightly toxic. |
| 93 | Zhushalian | Beishesheng | *Aristolochiaceae* | *Aristolochia tuberosa* C. F. Liang & S. M. Hwang | Rhizome | Cold in nature, with a bitter and pungent flavor. It clears heat, detoxifies, regulates spasms, reduces reflux, alleviates swelling, and pain. |
| 94 | Qixinglian | Yebaicai, Tinongcao, Dibaicao | *Violaceae* | *Viola diffusa* Ging. in DC. | Botanical drug | Cold in nature and bitter in flavor. It clears heat, detoxifies, promotes urination, and aids in the discharge of pus. |
| 95 | Zhujielian* | Danzhuhua, Wanshouzhu, Baiweisun, Zhulinxiao | *Colchicaceae* | *Disporum uniflorum* Baker ex S. Moore | Roots and rhizomes | Neutral in nature, with a flavor that is sweet and slightly sour. It tonifies the three energies, moistens the lungs, alleviates coughing, and bleeding. |
| 96 | Jingoulian | Shuanggoucao, Jingouteng | *Rubiaceae* | *Uncaria sinensis* (Oliv.) Havil. | Stem | Cold in nature, with a flavor that is both sweet and bitter. It drives away the wind, clears heat, dispels poison, and halts shock. |

*no pharmacological data available

**References**

Fang, Z.X., Zhao, H., Zhao, J.H., 2007. Tujia Medicinal Chronicles (土家族药物志), China.

Yang, D. S., Li, L. H., Tian, X. R., and Yang, D. Q. 2016a. Practical Tujia ethnic medicine (实用土家族药物), China.

Yuan, D. P. 2007. Practical Tujia Medicine (实用土家族医药), China.
