# Supplementary material for: Comprehensive review of tujia “Lian” medicinal botanical drugs: traditional classification system, phytochemical, and pharmacological profile
Source: Front Pharmacol. 2026 Feb 18;17:1747999. doi: 10.3389/fphar.2026.1747999 (PMC12957786; doi:10.3389/fphar.2026.1747999)
Supplement: Supplementary file 2 [file Table2.docx]

**SUPPLEMENTARY TABLE 2 "Lian" drugs and their anti-inflammatory and analgesic effects.**

| **Sr. No.** | **"Lian" drugs**  **name** | **Source** | **Study** | **Model/Assay** | **Conc. /Dose range** | **Effective sites or metabolites** |
| --- | --- | --- | --- | --- | --- | --- |
|  | Chuanxinlian | *Aconitum sinomontanum* Nakai | *In vitro/ In vivo* | BV-2 murine microglial cells, CFA model, AAW model, RAW 264.7 cells, XME model | 1.60-16.20 *µ*M, 0.75-160 mg/kg | Lappaconitine (Xing et al., 2025); Lappaconitine hydrochloride (Li et al., 2022); Diterpenoid alkaloids (Li et al., 2021); Lappaconitine, N-deacetyllappaconitine, Ranaconitine (Zhang et al., 2020a); Alkaloid Lappaconitine (Chunyan et al., 2022) |
|  | Huoxuelian | *Adenocaulon himalaicum* Edgew. | *In vitro* | IL-1*β*-stimulated rat chondrocytes | 6.25-100 *µ*g/mL | 70% ethanol extract (Lee et al., 2025e) |
|  | Qiyelian | *Aesculus chinensis* Bunge and *Aesculus chinensis* *var. wilsonii* (Rehder) Turland & N. H. Xia | *In vitro/ In vivo* | CUMS, RAW 264.7 cells, DSS model, Inflammatory cytokines model | 0.63-10 *µ*M, 0.5-10 mg/kg/day | Aescin (Liu et al., 2024); Flavonoids (Cao et al., 2023); Escin Ia (Yan et al., 2025); Flavonoids, Phenolic acids (Zhang et al., 2024); Sodium aescinate (Wang et al., 2025a) |
|  | Leigonglian | *Amydrium sinense* (Engl.) H. Li | *In vivo* | ISO model | 4-8 g/kg/day | Water extract (Wu et al., 2024a) |
|  | Dengtailian | *Arisaema heterophyllum* Blume*, Arisaema erubescens* (Wall.) Schott and *Arisaema amurense* Maxim. | *In vitro/ In vivo* | CIA Model (Collagen), CIA model, RAW 264.7 cells | 25-100 *µ*g/mL, 1-4 g/kg, 4.50-18 g/kg/day | Aqueous extract, Ethanol extract (Lianrui and Mingsan, 2023) |
|  | Baierlian | *Asparagus cochinchinensis* (Lour.) Merr. | *In vitro/ In vivo* | Loperamide-induced constipation model, ApoE-/- + HFD model, RAW 264.7 cells, BV-2 murine microglial cells | 10-80 *µ*M, 25-100 *µ*g/mL, 500-1000 mg/kg, 200-400 mg/kg/day | Aqueous extract (Kim et al., 2019); Asparagus polysaccharide (Zhu et al., 2025); Phenylpropanoid derivatives (Yue et al., 2022); Ethyl acetate extract, Methyl protodioscin, Ethanol extract (Wang et al., 2022b) |
|  | Daoshenglian | *Asplenium prolongatum* Hook*.* | *In vivo* | XME model, AAW model | 0.10-0.20 mL/kg | Volatile oil (Shuang et al., 2019) |
|  | Guanyinlian | *Balanophora fungosa* J. R. Forst. & G. Forst. | *In vitro* | RAW 264.7 cells | 10 *µ*M | Butenolide (Zhou et al., 2021) |
|  | Guanyinlian | *Balanophora involucrata* Hook. f. | *In vitro* | RAW 264.7 cells | 6.25-100 *µ*g/mL | β-sitosterol, quercetin (Zhang et al., 2023b) |
|  | Qiaozilian | *Begonia grandis subsp. sinensis* (A. DC.) Irmsch. | *In vivo* | Bacteria-induced chronic pelvic inflammatory disease model | 5-15 g/kg/day | Ethanol extract (Dan-ping et al., 2015) |
|  | Dahanliancao | *Bidens tripartita* L. | *In vitro/ In vivo* | PBMCs, CIA model, OXA model | 50 *µ*g/mL, 100-300 mg/kg | Hydro-methanolic extracts (Antoniak et al., 2023); Cynaroside (Szekalska et al., 2020); Aqueous infusion (Pozharitskaya et al., 2010) |
|  | Huoxuelian | *Bistorta officinalis* Raf. | *In vitro/ In vivo* | CIA model, NHDF cells, NSAID ulcer model, DSS model | 0.1-10 *µ*M, 1-100 *µ*g/mL, 50-500 mg/kg, 2.5-10 g/kg | Polyphenolic metabolites (Duwiejua et al., 1999); Aqueous extract, Ellagic acid, Procyanidins, Catechin (Pawłowska et al., 2020); Hydro-alcoholic extract, Aqueous extract (Yulan et al., 2025) |
|  | Tiexianlian | *Clematis chinensis* Osbeck | *In vitro/ In vivo* | LPS model, RAW 264.7 cells, DSS model, AIA model, DMM, IL-1β-treated chondrocytes, PO model, CIA Model (Collagen) | 1-10 *µ*M, 50-200 *µ*g/mL, 10-200 mg/kg, 20-80 mg/kg/day, | Hederagenin (Lee et al., 2015); Clematichinenoside AR (Xiong et al., 2014); Indole alkaloids (Jiang et al., 2023a); Processed ethanol extract (Jiang et al., 2022); Asperosaponin VI (Qiao et al., 2025); Total saponin fraction (Liu et al., 2025d); CCP-1 (Wu et al., 2025); Clemomanshurinane C and D, Extract (Lin et al., 2021) |
|  | Zhuyetiexianlian | *Clematis terniflora* DC. | *In vitro/ In vivo* | RAW 264.7 cells, CIA model, AAW model, Formalin test, CIA-CNP model | 1-40 *µ*M, 12.50-100 *µ*g/mL, 25-400 mg/kg, 200 mg/kg/day | Ethanol extracts, Aurantiamide acetate (Liu et al., 2015); Phenols, lignan (Dong et al., 2024); Clemanphentetraol A, Clemanphenol D (Shao et al., 2024); Ethyl acetate extract (Chen et al., 2011) |
|  | Babaolian | *Clerodendrum bungei* Steud. | *In vitro/ In vivo* | RAW 264.7 cells, H2O2-PC12, XME model, AAW model | 12.5-100 *µ*g/mL, 25-100 mg/kg, | Total flavonoid extract (He et al., 2022b) |
|  | Niuxuelian | *Dioscorea cirrhosa* Lour. | *In vivo* | DSS model | 100-200 mg/kg | *n*-Butanol fraction (Wang et al., 2025e) |
|  | Yeshulian | *Dioscorea polystachya* Turcz. | *In vitro/ In vivo* | CIA Model (Collagen)， CD4+ T cells， RAW 264.7 cells， ALI | 0.10-10 *µ*M，100 mg/kg， 20-40 mg/kg/day | Dioscin (Cao et al., 2019); Inosine (Liu et al., 2025e) |
|  | Guanyinlian | *Dioscorea zingiberensis* C. H. Wright | *In vitro/*  *In vivo* | MCAO/R model, H/R model, MI/R model, Caerulein-treated acinar cells, CER-AP model, L-Arg SAP model, AIA model | 2.50-10 *µ*M, 5-200 mg/kg | Steroidal saponin Deltonin (Zhang et al., 2020c); Steroid saponins (Xue et al., 2021); Dihydrodiosgenin (Shen et al., 2018); Phenolic metabolites (Du et al., 2017); Total steroidal saponin (Zhang et al., 2018a) |
|  | Bajiaolian | *Dysosma versipellis* (Hance) M. Cheng | *In vitro* | BU-HPLC/MS | NIL | Lignans and flavonoids (Feng et al., 2022) |
|  | Mohanlian | *Eclipta prostrata* (L.) L. | *In vitro/*  *In vivo* | OVA model, HRMCs | 5-20 *µ*M, 100-300 mg/kg | Methanolic extract (Morel et al., 2024); Wedelolactone (Feng et al., 2019) |
|  | Qiyelian | *Gynostemma pentaphyllum* (Thunb.) Makino | *In vitro/*  *In vivo* | OA chondrocytes, ATDC5 cells, RAW 264.7 cells, ALI, AIRAP Model, DSS model | 1-20 *µ*M, 12.50-100 *µ*g/mL, 10-50 mg/kg, 10-100 mg/kg/day, 1.75-7 g/kg | Extract, Gypenoside L, Gypenoside LI (Lee et al., 2025c); Flavonoids and saponins (Xie et al., 2024); Polysaccharides (Hu et al., 2025); Extract, Quercetin, Kaempferol (Zhong et al., 2025); Gypenoside LXXV (Wu et al., 2024c); Gypenoside XLIX (Ping et al., 2025) |
|  | Baiweilian | *Hemsleya chinensis* Cogn. ex F. B. Forbes & Hemsl. | *In vitro/*  *In vivo* | RAW 264.7 cells, ALI model | 1-40 *µ*M, 100-400 *µ*g/mL, 200 mg/kg | Cucurbitane type triterpenoids (Lian et al., 2023); Cucurbitane glycosides (Chi et al., 2025); Oligosaccharides (Ma et al., 2024c) |
|  | Honghanlian | *Hypericum ascyron* L. | *In vitro/*  *In vivo* | PM (SRM 2786) model, HNE inhibition assay | 0.85 *µ*M, 100-200 mg/kg | Extract (Lee et al., 2020); Polyprenylated acylphloroglucinol derivatives (Li et al., 2019) |
|  | Duiyuelian | *Hypericum sampsonii* Hance | *In vitro/*  *In vivo* | RAW 264.7 cells, DSS model, LPS-induced sepsis model, AAW model, HPT, CIA model, XME model | 0.78-25 *µ*M, 20-50 mg/kg, 5-50 mg/kg/day | Xanthones, Phloroglucinol derivatives (Chen et al., 2020); Hyperampsonone H (Li et al., 2024b); Polycyclic polyprenylated acylphloroglucinols (Li et al., 2023c); Ethanol extract (Hsu et al., 2023); 4-geranyloxy-2,6-dihydroxybenzophenone (Wang et al., 2023); Extracts, Phloroglucinol derivatives, xanthones (Sun et al., 2023c) |
|  | Yimulian | *Leonurus japonicus* Houtt. | *In vitro/*  *In vivo* | HEECs, AE, Inflammatory cytokines, TFL, HPT | 25-100 *µ*g/mL, 25-50 mg/kg, 1.25-5 g/kg | Alkaloid (Dai et al., 2025); Ethanol extract (Zhang et al., 2023a) |
|  | Banbianlian | *Lobelia chinensis* Lour. | *In vitro/*  *In vivo* | HaCaT cells, Human skin explant culture model, MC903 model, RTCA, RAW 264.7 cells, CIA model, FMLP/cytochalasin B-stimulated human neutrophils | 0.10- 25 *µ*M, 10-200 *µ*g/mL, 100-200 mg/kg | Ethanol extract; Diosmetin (Park et al., 2022); Flavonoid glycosides (Wang et al., 2019); Aqueous extract (Li et al., 2015a); Lobechine, DMB, Vanillic acid (Kuo et al., 2011) |
|  | Qikonglian | *Osbeckia stellata* Buch.-Ham. ex D. Don | *In vitro/ In vivo* | RAW 264.7 cells, HCl/ethanol-induced acute gastritis model | 12.5-100 *µ*g/mL, 50-100 mg/kg | Methanol extract (Yang et al., 2012) |
|  | Qiyelian | *Paris polyphylla var. Chinensis* (Franch.) | *In vitro* | RAW 264.7 cells | 1-10 *µ*M | Steroidal saponins (Guan et al., 2024) |
|  | Jiujielian | *Peristrophe japonica* (Thunb.) Bremek. | *In vivo* | XME model, AAW model, HPT | 5-20 g/kg | Ethanol extract (Qin and Luo, 2006) |
|  | Yanqiaolian | *Persicaria capitata* (Buch. Ham. ex D. Don) H. Gross | *In vitro/*  *In vivo* | *H. pylori*-infected AGS cells, H. Pylori model, PO/HX model, MSU crystal model, XME model, AAW model | 12.50-100 *µ*M, 0.75-30 g/kg, 50 mg/kg/day | Quercetin (Zhang et al., 2017); Aqueous extract (Zhang et al., 2021a); Water extract (YUlan et al., 2021) |
|  | Qiaokelian | *Persicaria chinensis* (L.) H. Gross | *In vitro/*  *In vivo* | Ethanol-induced gastric ulcer model, RAW 264.7 cells, ALI, IAV-ALI model | 10-100 *µ*g/mL, 10-500 mg/kg, 3.90-7.80 g/kg/day | Aqueous extract (Ismail et al., 2012); Total flavonoids (Tao et al., 2016); Methanol extract (Hossen et al., 2015); Water decoction, Total flavonoids, Aurantiamide acetate (Fengfeng and Hua, 2025) |
|  | Guanyinzuolian | *Phedimus aizoon* (L.) 't Hart | *In vitro/*  *In vivo* | RAW 264.7 cells, Inflammatory cytokines, TPA model, CIA model | 50-800 mg/kg | Several extracts (PE, EtOAc, and H_2_O) (Wang et al., 2024a) |
|  | Luanjiaolian | *Pholidota yunnanensis* Rolfe | *In vitro* | RAW 264.7 cells, BV-2 cells | 1.25-57.70 *µ*M | Stilbenoids (Guo et al., 2006); Dihydrophenanthrene dimers (Qi et al., 2024) |
|  | Jixuelian | *Pronephrium penangianum* (Hook.) Holttum | *In vitro* | Activated pancreatic stellate cell model | 5-40 *µ*M | Ethanol extracts, Eruberin A (Tsang et al., 2015) |
|  | Runxuelian | *Pyrola calliantha* Andres | *In vitro/*  *In vivo* | RAW 264.7 cells, CIA Model (Collagen), XME model, AAW model | 10-100 *µ*g/mL, 25-100 mg/kg, 50-200 mg/kg/day | Ethanol extracts (Lee et al., 2007); Extract, Hyperin (He et al., 2022a); |
|  | Matilian | *Rheum* *palmatum* L. | *In vitro/*  *In vivo* | NRCFs, ISO model | 2.50-20 *µ*M, 10-20 mg/kg/day | Chrysophanol (Liu et al., 2025b) |
|  | Baihelian | *Saururus chinensis* (Lour.) Baill. | *In vitro/*  *In vivo* | RAW 264.7 cells, OVA model, Clinical Trial, DSS model, LPS model | 0.1-50 *µ*M, 10-100 *µ*g/mL, 100 mg/kg, 1800 mg/day, 100-200 mg/kg/day | Ethanol extract (Meng et al., 2016); Lignans (Jung et al., 2019); Extract (Lee et al., 2025a); LHF618 (Lee et al., 2025d); Methanol extract (Zhang et al., 2021b); Neolignan (Yoo et al., 2020) |
|  | Jizhualian | *Sceptridium ternatum* (Thunb.) Lyon | *In vivo* | OXA model | 0.5%-1% (weight/weight). | Methanol extracts(Lim et al., 2015) |
|  | Banzhilian | *Scutellaria barbata* D. Don | *In vitro/*  *In vivo* | RAW 264.7 cells, IL-1*β*, DMM, MPC-83 cells, AP, SAP, Calu-3 cells, ALI, FMLP, ARDS, UC | 1-50 *µ*M, 10-200 *µ*g/mL, 25-50 mg/kg, 100-500 mg/kg/day | Ethyl acetate fraction (Liu et al., 2018); Scutellarein (Ye et al., 2024); Eriodictyol (Tian et al., 2025); Extracts (Ran et al., 2025); Hot water extract (Chen et al., 2025); Neo-clerodane diterpenoids (Li et al., 2023b); Polysaccharides (Wu et al., 2022) |
|  | Xinyebanzhilian | *Scutellaria indica* L. | *In vitro* | RAW 264.7 cells | 1-30 *µ*M | Cupatolit and tilianin (Cuong et al., 2015) |
|  | Gouyabanzhilian | *Sedum sarmentosum* Bunge | *In vitro/*  *In vivo* | RAW 264.7 cells, LPS-AKI model, NR8383 cells, NaT-SAP-ALI model, NRK-52E cells | 6.25-100 *µ*M, 12.50-100 *µ*g/mL, 200-400 mg/kg | Ethanol extracts (Lu et al., 2019); Sarmentosin and quercetin (Guo et al., 2024a); Extracts, Sarmentosin, Luteolin, Flavonoid glycosides (Ying-Ying et al., 2020) |
|  | Bagualian | *Sinopodophyllum hexandrum* (Royle) T. S. Ying | *In vitro* | RAW 264.7 cells | 1-40 *µ*M | Podophyllotoxin (PTOX) (Liu et al., 2025f) |
|  | Bixuelian | *Stephania japonica* (Thunb.) Miers | *In vitro/*  *In vivo* | RAW 264.7 cells, MCAO/R model | 0.10-1.00 *µ*M, 2.50-10 mg/kg | Stepharine (Hao et al., 2020); Ceparanthine (Lin et al., 2019) |
|  | Maweilian | *Thalictrum minus var. hypoleucum* (Siebold & Zucc.) Miq. | *In vitro/*  *In vivo* | ALI, RAW 264.7 cells, Inflammatory cytokines model | 6.25-50 *µ*M, 20-100 mg/kg | Extract (Badamjav et al., 2021); Isoorientin (Zhang et al., 2022b) |
|  | Jingoulian | *Uncaria sinensis* (Oliv.) Havil. | *In vitro/*  *In vivo* | BV-2 murine microglial cells, tMCAO model | 1-100 *µ*g/mL, 100-300 mg/kg | Hexane extracts (Kang et al., 2015) |
|  | Shuikulian | *Veronica anagallis-aquatica* L. | *In vivo* | P-Benzoquinone-induced writhing test, CIA model | 100 mg/kg | Iridoid glucosides (Küpeli et al., 2005) |
|  | Qixinglian | *Viola diffusa* Ging. in DC. | *In vitro/*  *In vivo* | THP-1, HUVEC, ALI | 12.50-100 *µ*g/mL, 25-50 mg/kg | VDP-1 (Dai et al., 2023) |

**References**

Antoniak, K., Studzińska-Sroka, E., Szymański, M., Dudek-Makuch, M., Cielecka-Piontek, J., Korybalska, K., 2023. Antiangiogenic, Anti-Inflammatory and Antioxidant Properties of Bidens tripartite Herb, Galium verum Herb and Rumex hydrolapathum Root. Molecules 28 (13) . <https://doi.org/10.3390/molecules28134966.>

Badamjav, R., Zhang, L., Sonom, D., Wu, Y.H., Kou, J.P., Yu, B.Y., Li, F., 2021. Thalictrum minus L. ameliorates particulate matter-induced acute lung injury in mice. J. Ethnopharmacol. 264, 113379. <https://doi.org/10.1016/j.jep.2020.113379.>

Cao, H.N., Ruan, J.Y., Han, Y., Zhao, W., Zhang, Y., Gao, C., Wu, H.H., Ma, L., Gao, X.M., Zhang, Y., Wang, T., 2023. NO Release Inhibitory Activity of Flavonoids from Aesculus Wilsonii Seeds through MAPK (P38), NF-κB, and STAT3 Cross-Talk Signaling Pathways. Planta Med. 89 (1), 46-61. <https://doi.org/10.1055/a-1789-2983.>

Cao, Y.J., Xu, Y., Liu, B., Zheng, X., Wu, J., Zhang, Y., Li, X.S., Qi, Y., Sun, Y.M., Wen, W.B., Hou, L., Wan, C.P., 2019. Dioscin, a Steroidal Saponin Isolated from Dioscorea nipponica, Attenuates Collagen-Induced Arthritis by Inhibiting Th17 Cell Response. Am. J. Chin. Med. 47 (2), 423-437. <https://doi.org/10.1142/S0192415X19500216.>

Chen, R.Z., Cui, L., Guo, Y.J., Rong, Y.M., Lu, X.H., Sun, M.Y., Zhang, L., Tian, J.K., 2011. In vivo study of four preparative extracts of Clematis terniflora DC. for antinociceptive activity and anti-inflammatory activity in rat model of carrageenan-induced chronic non-bacterial prostatitis. J. Ethnopharmacol. 134 (3), 1018-1023. <https://doi.org/10.1016/j.jep.2011.01.004.>

Chen, W., Shen, X., Ma, L., Chen, R., Yuan, Q., Zheng, Y., Li, C., Peng, G., 2020. Phenolic Compounds from Polygonum chinense Induce Growth Inhibition and Apoptosis of Cervical Cancer SiHa Cells. Biomed Res Int 2020, 8868508. <https://doi.org/10.1155/2020/8868508.>

Chen, Y.C., Lee, Y.R., Chang, Y.C., Wang, Y.H., Fang, S.Y., Lin, C.H., Chen, P.J., Hwang, T.L., 2025. Scutellaria barbata ameliorates acute respiratory distress syndrome by inhibiting neutrophil-mediated inflammatory responses. J. Ethnopharmacol. 346, 119653. <https://doi.org/10.1016/j.jep.2025.119653.>

Chi, J., Li, M., Lian, F., Li, Y., Dai, L., 2025. Cucurbitane Glycosides and Their Potential Anti-Inflammatory Activities from Hemsleya chinensis Tubers. Molecules 30 (11) . <https://doi.org/10.3390/molecules30112349.>

Chunyan, J., Xiaolou, M., Yunhe, Z., Wenguang, Z., Dan, W., Yun, L.I., 2022. Research Progress on Processing Evolution, Chemical Compositions and Pharmacological Effects of Aconitum sinomontanum Nakai. Chinese Journal of Information on Traditional Chinese Medicine 29 (01), 143-148. <https://doi.org/10.19879/j.cnki.1005-5304.202105299.>

Cuong, T.D., Hung, T.M., Lee, J.S., Weon, K.Y., Woo, M.H., Min, B.S., 2015. Anti-inflammatory activity of phenolic compounds from the whole plant of Scutellaria indica. Biorg. Med. Chem. Lett. 25 (5), 1129-1134. <https://doi.org/10.1016/j.bmcl.2014.12.055.>

Dai, N., Li, G., Ni, J., Li, F., Tong, H., Liu, Y., 2023. A novel galactoxylan derived from Viola diffusa alleviates LPS-induced acute lung injury via antagonizing P-selectin-mediated adhesion function. Int. J. Biol. Macromol 242 (Pt 2), 124821. <https://doi.org/10.1016/j.ijbiomac.2023.124821.>

Dai, O., Fan, Y., Zhou, Q., Liu, J., Zuo, J., Wang, F., Li, L., Wang, F., Xiong, L., 2025. Effect of Leonurus japonicus alkaloids on endometrial inflammation and its mechanisms. J. Ethnopharmacol. 342, 119432. <https://doi.org/10.1016/j.jep.2025.119432.>

Dan-ping, L.I., Zhi-chao, T.U., Jing, N., Kai-yong, H.E., Xiao-jun, L., Li-hui, Z., Jiao, P., 2015. Influence of Effective Parts of Begonia grandis sinensis on Chronic Pelvic Inflammatory Disease Rats. Chinese Journal of Experimental Traditional Medical Formulae 21 (10), 166-169. <https://doi.org/10.13422/j.cnki.syfjx.2015100166.>

Dong, S., Zhang, J.Y., Zhao, J.L., Li, G.Q., Yan, B.X., Lv, C.N., Yuan, J.Z., Lu, J.C., 2024. Three new phenols and one new lignan from Clematis terniflora var. manshurica (Rupr.) Ohwi with their anti-inflammatory activity. Fitoterapia 177, 106043. <https://doi.org/10.1016/j.fitote.2024.106043.>

Du, D., Jin, T., Zhang, R., Hu, L., Xing, Z., Shi, N., Shen, Y., Gong, M., 2017. Phenolic compounds isolated from Dioscorea zingiberensis protect against pancreatic acinar cells necrosis induced by sodium taurocholate. Biorg. Med. Chem. Lett. 27 (6), 1467-1470. <https://doi.org/10.1016/j.bmcl.2017.01.014.>

Duwiejua, M., Zeitlin, I.J., Gray, A.I., Waterman, P.G., 1999. The anti-inflammatory compounds of Polygonum bistorta: isolation and characterisation. Planta Med. 65 (4), 371-374. <https://doi.org/10.1055/s-2006-960791.>

Feng, H., Chen, G., Zhang, Y., Guo, M., 2022. Potential Multifunctional Bioactive Compounds from Dysosma versipellis Explored by Bioaffinity Ultrafiltration-HPLC/MS with Topo I, Topo II, COX-2 and ACE2. J Inflamm Res 15, 4677-4692. <https://doi.org/10.2147/JIR.S371830.>

Feng, L., Zhai, Y.Y., Xu, J., Yao, W.F., Cao, Y.D., Cheng, F.F., Bao, B.H., Zhang, L., 2019. A review on traditional uses, phytochemistry and pharmacology of Eclipta prostrata (L.) L. J. Ethnopharmacol. 245, 112109. <https://doi.org/10.1016/j.jep.2019.112109.>

Fengfeng, X., Hua, Z., 2025. Research Progress on Chemical Constituents and Pharmacological Effects of Huotanmu(Herba Polygoni Chinensislor). Journal of Liaoning University of Traditional Chinese Medicine 27 (04), 182-192. <https://doi.org/10.13194/j.issn.1673-842X.2025.04.035.>Guan, L., Zheng, Z., Guo, Z., Xiao, S., Liu, T., Chen, L., Gao, H., Wang, Z., 2024. Steroidal saponins from rhizome of Paris polyphylla var. chinensis and their anti-inflammatory, cytotoxic effects. Phytochemistry 219, 113994. <https://doi.org/10.1016/j.phytochem.2024.113994.>

Guo, R., Yu, K., Huang, K., Li, J., Huang, J., Yang, X., et al. (2024a). Regulatory mechanism of Sarmentosin and Quercetin on lipid accumulation in primary hepatocyte of GIFT tilapia (Oreochromis niloticus) with fatty liver. *PLoS. ONE* 19 (9), e0309976. doi:10.1371/journal.pone.0309976

Guo, X.Y., Wang, J., Wang, N.L., Kitanaka, S., Liu, H.W., Yao, X.S., 2006. New stilbenoids from Pholidota yunnanensis and their inhibitory effects on nitric oxide production. Chem. Pharm. Bull. (Tokyo) 54 (1), 21-25. <https://doi.org/10.1248/cpb.54.21.>

Hao, T., Yang, Y., Li, N., Mi, Y., Zhang, G., Song, J., Liang, Y., Xiao, J., Zhou, D., He, D., Hou, Y., 2020. Inflammatory mechanism of cerebral ischemia-reperfusion injury with treatment of stepharine in rats. Phytomedicine 79, 153353. <https://doi.org/10.1016/j.phymed.2020.153353.>

He, C., Liu, J., Ke, T., Luo, Y., Zhang, S., Mao, T., et al. (2022a). Pyrolae herba: A review on its botany, traditional uses, phytochemistry, pharmacology and quality control. *J. Ethnopharmacol.* 298 115584. doi:10.1016/j.jep.2022.115584

He, Z., Fei, C., You, Q., and Shui-han, Z. (2022b). Research Progress on Chemical Constituents of Clerodendrum bungei Steud. and Its Pharmacological Activities. *Journal of Nanjing University of Traditional Chinese Medicine* 38 (04), 361-374. doi:10.14148/j.issn.1672-0482.2022.0361

Hossen, M.J., Kim, S.C., Son, Y.J., Baek, K.S., Kim, E., Yang, W.S., Jeong, D., Park, J.G., Kim, H.G., Chung, W.J., Yoon, K., Ryou, C., Lee, S.Y., Kim, J.H., Cho, J.Y., 2015. AP-1-Targeting Anti-Inflammatory Activity of the Methanolic Extract of Persicaria chinensis. Evid Based Complement Alternat Med 2015, 608126. <https://doi.org/10.1155/2015/608126.>

Hsu, Y.C., Ou, S.M., Zhuang, K.R., Kuo, A.L., Li, W.J., Huang, C.Y., Lin, C.H., Chen, J.J., Fu, S.L., 2023. Hypericum sampsonii exhibits anti-inflammatory activity in a lipopolysaccharide-induced sepsis mouse model. J Tradit Complement Med 13 (4), 379-388. <https://doi.org/10.1016/j.jtcme.2023.03.002.>

Hu, X., Wang, W., Zhang, D., Tian, X., Zhang, A., Xu, J., Feng, F., Li, W., Kikuchi, T., Zhang, J., 2025. Phytochemistry, bioactivities and application of Gynostemma pentaphyllum polysaccharide: A review. Int. J. Biol. Macromol 322 (Pt 1), 144964. <https://doi.org/10.1016/j.ijbiomac.2025.144964.>

Ismail, I.F., Golbabapour, S., Hassandarvish, P., Hajrezaie, M., Abdul Majid, N., Kadir, F.A., Al-Bayaty, F., Awang, K., Hazni, H., Abdulla, M.A., 2012. Gastroprotective Activity of Polygonum chinense Aqueous Leaf Extract on Ethanol-Induced Hemorrhagic Mucosal Lesions in Rats. Evid Based Complement Alternat Med 2012, 404012. <https://doi.org/10.1155/2012/404012.>

Jiang, H. B., Shao, S. Y., Wei, Y. Z., Lin, M. B., and Li, S. (2023a). Phenolic glycosides and indole alkaloids isolated from the roots and rhizomes of Clematis chinensis and their anti-inflammatory activity. *Phytochemistry* 215 113832. doi:10.1016/j.phytochem.2023.113832

Jiang, S.Q., Guo, Z.J., Pan, T., Xu, X.X., Yang, Y.N., Wang, H.Y., Li, P., Li, F., 2022. The multi-omics and analysis to reveal thermal processing enhanced anti-rheumatoid arthritis efficacy of Radix Clematidis in rats. J. Pharm. Biomed. Anal. 215, 114760. <https://doi.org/10.1016/j.jpba.2022.114760.>

Jung, Y.W., Lee, B.M., Ha, M.T., Tran, M.H., Kim, J.A., Lee, S., Lee, J.H., Woo, M.H., Min, B.S., 2019. Lignans from Saururus chinensis exhibit anti-inflammatory activity by influencing the Nrf2/HO-1 activation pathway. Arch. Pharm. Res. 42 (4), 332-343. <https://doi.org/10.1007/s12272-018-1093-4.>

Kang, B.K., Kim, M.K., Kim, S.Y., Lee, S.J., Choi, Y.W., Choi, B.T., Shin, H.K., 2015. Anti-Neuroinflammatory Effects of Uncaria sinensis in LPS-Stimulated BV2 Microglia Cells and Focal Cerebral Ischemic Mice. Am. J. Chin. Med. 43 (6), 1099-1115. <https://doi.org/10.1142/S0192415X15500639.>

Kim, J.E., Park, J.W., Kang, M.J., Choi, H.J., Bae, S.J., Choi, Y.S., Lee, Y.J., Lee, H.S., Hong, J.T., Hwang, D.Y., 2019. Anti-Inflammatory Response and Muscarinic Cholinergic Regulation during the Laxative Effect of Asparagus cochinchinensis in Loperamide-Induced Constipation of SD Rats. Int. J. Mol. Sci. 20 (4), 946. <https://doi.org/10.3390/ijms20040946.>

Kuo, P.C., Hwang, T.L., Lin, Y.T., Kuo, Y.C., Leu, Y.L., 2011. Chemical constituents from Lobelia chinensis and their anti-virus and anti-inflammatory bioactivities. Arch. Pharm. Res. 34 (5), 715-722. <https://doi.org/10.1007/s12272-011-0503-7.>

Küpeli, E., Harput, U.S., Varel, M., Yesilada, E., Saracoglu, I., 2005. Bioassay-guided isolation of iridoid glucosides with antinociceptive and anti-inflammatory activities from Veronica anagallis-aquatica L. J. Ethnopharmacol. 102 (2), 170-176. <https://doi.org/10.1016/j.jep.2005.05.042.>

Lee, C.W., Park, S.M., Zhao, R., Lee, C., Chun, W., Son, Y., Kim, S.H., Jung, J.Y., Jegal, K.H., Cho, I.J., Ku, S.K., Kim, Y.W., Ju, S.A., Kim, S.C., An, W.G., 2015. Hederagenin, a major component of Clematis mandshurica Ruprecht root, attenuates inflammatory responses in RAW 264.7 cells and in mice. Int. Immunopharmacol. 29 (2), 528-537. <https://doi.org/10.1016/j.intimp.2015.10.002.>

Lee, H. J., Kim, K. C., Lee, W. J., Nam, H. J., Ryu, S. J., Kim, S. H., et al. (2025a). Therapeutic effects of a combination of Chinese quince and Saururus chinensis extract on allergic airway inflammation in an ovalbumin-induced asthma mouse model. *Front Nutr* 12 1613413. doi:10.3389/fnut.2025.1613413

Lee, M.H., Lee, J.M., Jun, S.H., Lee, S.H., Kim, N.W., Lee, J.H., Ko, N.Y., Mun, S.H., Kim, B.K., Lim, B.O., Choi, D.K., Choi, W.S., 2007. The anti-inflammatory effects of Pyrolae herba extract through the inhibition of the expression of inducible nitric oxide synthase (iNOS) and NO production. J. Ethnopharmacol. 112 (1), 49-54. <https://doi.org/10.1016/j.jep.2007.01.036.>

Lee, S. A., Lee, C. H., Lee, S. H., Do, E., Kim, D. K., Huh, T. L., et al. (2025c). Inhibitory Effects of Heat-Processed Gynostemma pentaphyllum Extract (Actiponin(®)) and Its Components on Cartilage Breakdown in Osteoarthritis. *Int. J. Mol. Sci.* 26 (4), 1728. doi:10.3390/ijms26041728

Lee, Y., Lee, J. K., Park, J., Kim, N., Kang, H. R., and Kwon, Y. (2025d). Efficacy and Safety of Saururus chinensis Extract (LHF618) for Treating Allergic Rhinitis: A Randomized, Double-Blind, Placebo-Controlled, Multicenter Clinical Study. *J. Med. Food* 28 (8), 802-811. doi:10.1089/jmf.2025.k.0008

Lee, Y. M., Son, E., Kim, D. S., Shim, K. S., and Yu, S. H. (2025e). Evaluating the Anti-Inflammatory and Chondroprotective Effects of Adenocaulon himalaicum Extract Through Network Pharmacology and Experimental Validation. *Int. J. Mol. Sci.* 26 (3) . doi:10.3390/ijms26030877

Lee, Y.Y., Yang, W.K., Han, J.E., Kwak, D., Kim, T.H., Saba, E., Kim, S.D., Lee, Y.C., Kim, J.S., Kim, S.H., Rhee, M.H., 2020. Hypericum ascyron L. extract reduces particulate matter-induced airway inflammation in mice. Phytother Res . <https://doi.org/10.1002/ptr.6929.>

Li, K. C., Ho, Y. L., Huang, G. J., and Chang, Y. S. (2015a). Anti-oxidative and anti-inflammatory effects of Lobelia chinensis in vitro and in vivo. *Am. J. Chin. Med.* 43 (2), 269-287. doi:10.1142/S0192415X15500184

Li, S., Xu, D., Jia, J., Zou, W., Liu, J., Wang, Y., et al. (2023b). Structure and anti-inflammatory activity of neo-clerodane diterpenoids from Scutellaria barbata. *Phytochemistry* 213 113771. doi:10.1016/j.phytochem.2023.113771

Li, X., Wang, X., Li, Z., Mao, Y., Liu, Z., Liu, X., Zhu, X., Zhang, J., 2022. A Metabolomic Study of the Analgesic Effect of Lappaconitine Hydrobromide (LAH) on Inflammatory Pain. Metabolites 12 (10), 923. <https://doi.org/10.3390/metabo12100923.>

Li, Y., Wang, M., Su, J., Wang, Y., Zhao, Z., and Sun, Z. (2023c). Polycyclic polyprenylated acylphloroglucinols from Hypericum sampsonii Hance and their anti-inflammatory activity. *Fitoterapia* 169 105610. doi:10.1016/j.fitote.2023.105610

Li, Y., Wang, M., Su, J., Zhong, R., Yin, S., Zhao, Z., et al. (2024b). Hypersampsonone H attenuates ulcerative colitis via inhibition of PDE4 and regulation of cAMP/PKA/CREB signaling pathway. *Int. Immunopharmacol.* 128 111490. doi:10.1016/j.intimp.2024.111490

Li, Y., Zeng, J., Tian, Y.H., Hou, Y., Da, H., Fang, J., Gao, K., 2021. Isolation, identification, and activity evaluation of diterpenoid alkaloids from Aconitum sinomontanum. Phytochemistry 190, 112880. <https://doi.org/10.1016/j.phytochem.2021.112880.>

Li, Z.P., Kim, J.Y., Ban, Y.J., Park, K.H., 2019. Human neutrophil elastase (HNE) inhibitory polyprenylated acylphloroglucinols from the flowers of Hypericum ascyron. Bioorg. Chem. 90, 103075. <https://doi.org/10.1016/j.bioorg.2019.103075.>

Lian, F., Chi, J., Meng, Q., Li, Q., Chen, A., Wang, Z., Dai, L., 2023. Cucurbitane triterpenes from Hemsleya chinensis tubers and their anti-inflammatory activities. Fitoterapia 166, 105441. <https://doi.org/10.1016/j.fitote.2023.105441.>

Lianrui, W., Mingsan, M., 2023. Research Progress of Arisaematis Rhizoma and Predictive Analysis on Its Q-marker. Traditional Chinese Drug Research and Clinical Pharmacology 34 (11), 1640-1647. <https://doi.org/10.19378/j.issn.1003-9783.2023.11.019.>

Lim, D., Kim, M.K., Jang, Y.P., Kim, J., 2015. Sceptridium ternatum attenuates allergic contact dermatitis-like skin lesions by inhibiting T helper 2-type immune responses and inflammatory responses in a mouse model. J. Dermatol. Sci. 79 (3), 288-297. <https://doi.org/10.1016/j.jdermsci.2015.06.012.>

Lin, T.F., Wang, L., Zhang, Y., Zhang, J.H., Zhou, D.Y., Fang, F., Liu, L., Liu, B., Jiang, Y.Y., 2021. Uses, chemical compositions, pharmacological activities and toxicology of Clematidis Radix et Rhizome- a Review. J. Ethnopharmacol. 270, 113831. <https://doi.org/10.1016/j.jep.2021.113831>

Lin, X., Song, F., Zhou, L., Wang, Z., Wei, C., Xu, J., Zhao, J., Liu, Q., 2019. Cepharanthine suppresses osteoclast formation by modulating the nuclear factor-κB and nuclear factor of activated T-cell signaling pathways. J. Cell Biochem. 120 (2), 1990-1996. <https://doi.org/10.1002/jcb.27495.>

Liu, C., Qiu, S., Liu, X., Huang, R., and Fang, Z. (2025b). Chrysophanol Attenuates Cardiac Fibrosis and Arrhythmia by Suppressing the Endoplasmic Reticulum Stress/Pyroptosis Axis and Inflammation. *Phytother Res* . doi:10.1002/ptr.8476

Liu, F., Jia, Y., Zhao, L., Xiao, L.N., Cheng, X., Xiao, Y., Zhang, Y., Zhang, Y., Yu, H., Deng, Q.E., Zhang, Y., Feng, Y., Wang, J., Gao, Y., Zhang, X., Geng, Y., 2024. Escin ameliorates CUMS-induced depressive-like behavior via BDNF/TrkB/CREB and TLR4/MyD88/NF-κB signaling pathways in rats. Eur. J. Pharmacol. 984, 177063. <https://doi.org/10.1016/j.ejphar.2024.177063.>

Liu, H.L., Kao, T.H., Shiau, C.Y., Chen, B.H., 2018. Functional components in Scutellaria barbata D. Don with anti-inflammatory activity on RAW 264.7 cells. J. Food Drug Anal. 26 (1), 31-40. <https://doi.org/10.1016/j.jfda.2016.11.022.>

Liu, S., Dong, H., Geng, W., Liu, Y., Arachchige, B., and Wang, X. (2025d). A new utilization of saponins from the root of Clematis chinensis Osbeck: Hypouricemia effect. *Bioorg. Chem.* 163 108727. doi:10.1016/j.bioorg.2025.10872

Liu, T., Tian, C., Li, H., Zhang, C., Duan, W., Yuan, J., et al. (2025e). Therapeutic potential of inosine in acute lung injury: mechanistic insights into TLR4 suppression and macrophage polarization. *Phytomedicine* 143 156854. doi:10.1016/j.phymed.2025.15685

Liu, W., Zhao, D., Yin, D., Duan, K., and Wang, Z. (2025f). Plant Origin Source, Content Profile and Bioactivity of Podophyllotoxin as an Important Natural Anticancer Agent. *Chem. Biodivers.* 22 (4), e202402375. doi:10.1002/cbdv.202402375

Liu, X.B., Yang, B.X., Zhang, L., Lu, Y.Z., Gong, M.H., Tian, J.K., 2015. An in vivo and in vitro assessment of the anti-inflammatory, antinociceptive, and immunomodulatory activities of Clematis terniflora DC. extract, participation of aurantiamide acetate. J. Ethnopharmacol. 169, 287-294. <https://doi.org/10.1016/j.jep.2015.04.009.>

Lu, H., Cheng, S., Wu, C., Zheng, S., Hong, W., Liu, L., Bai, Y., 2019. Sedum sarmentosum Bunge extract alleviates inflammation and kidney injury via inhibition of M1-macrophage polarization. Phytomedicine 62, 152976. <https://doi.org/10.1016/j.phymed.2019.152976.>

Ma, Y., Zhang, F., Xie, Y., An, L., Zhang, B., Yu, B., et al. (2024c). Oligosaccharides from Asparagus cochinchinensis for ameliorating LPS-induced acute lung injury in mice. *Food Funct.* 15 (5), 2693-2705. doi:10.1039/d3fo05628g

Meng, X., Kim, I., Jeong, Y.J., Cho, Y.M., Kang, S.C., 2016. Anti-inflammatory effects of Saururus chinensis aerial parts in murine macrophages via induction of heme oxygenase-1. Experimental biology and medicine (Maywood, N.J.) 241 (4), 396-408. <https://doi.org/10.1177/1535370215614657.>

Morel, L., Carmona, F., Guimarães, C.C., Moreira, L., Leão, P., Crevelin, E.J., Batah, S.S., Fabro, A.T., França, S.C., Borges, M.C., Pereira, A., 2024. A methanolic extract of Eclipta prostrata (L.) L. decreases inflammation in a murine model of chronic allergic asthma via inhibition of the NF-kappa-B pathway. J. Ethnopharmacol. 318 (Pt B), 116930. <https://doi.org/10.1016/j.jep.2023.116930.>

Park, N.J., Jo, B.G., Bong, S.K., Park, S.A., Lee, S., Kim, Y.K., Yang, M.H., Kim, S.N., 2022. Lobelia chinensis Extract and Its Active Compound, Diosmetin, Improve Atopic Dermatitis by Reinforcing Skin Barrier Function through SPINK5/LEKTI Regulation. Int. J. Mol. Sci. 23 (15) . <https://doi.org/10.3390/ijms23158687.>

Pawłowska, K.A., Hałasa, R., Dudek, M.K., Majdan, M., Jankowska, K., Granica, S., 2020. Antibacterial and anti-inflammatory activity of bistort (Bistorta officinalis) aqueous extract and its major components. Justification of the usage of the medicinal plant material as a traditional topical agent. J. Ethnopharmacol. 260, 113077. <https://doi.org/10.1016/j.jep.2020.113077.>

Ping, K., Yang, R., Chen, H., Xie, S., Xiang, Y., Li, M., Lu, Y., Dong, J., 2025. Gypenoside XLIX Activates the Sirt1/Nrf2 Signaling Pathway to Inhibit NLRP3 Inflammasome Activation to Alleviate Septic Acute Lung Injury. Inflammation 48 (1), 42-60. <https://doi.org/10.1007/s10753-024-02041-2>

Pozharitskaya, O.N., Shikov, A.N., Makarova, M.N., Kosman, V.M., Faustova, N.M., Tesakova, S.V., Makarov, V.G., Galambosi, B., 2010. Anti-inflammatory activity of a HPLC-fingerprinted aqueous infusion of aerial part of Bidens tripartita L. Phytomedicine 17 (6), 463-468. <https://doi.org/10.1016/j.phymed.2009.08.001.>

Qi, J., Jia, Y., Zhou, D., Chen, G., Hao, J., Mi, Y., Xu, L., Lin, B., Hou, Y., Li, N., 2024. Axially chiral dihydrophenanthrene dimers from Pholidota yunnanensis with anti-neuroinflammatory activities. Bioorg. Chem. 150, 107570. <https://doi.org/10.1016/j.bioorg.2024.107570.>

Qiao, J., Feng, R., Yang, G., Yang, Z., Zhang, A., Xu, F., 2025. Asperosaponin VI mitigates mitochondrial dysfunction and chondrocyte apoptosis in osteoarthritis by modulating the AMPK-SIRT3 pathway. Cell Biol. Toxicol. 41 (1), 120. <https://doi.org/10.1007/s10565-025-10071-1.>

Qin, R.G., Luo, Z.S., 2006. Experimental Study on the Pharmacodynamics of Peristrophe japonica (Thunb.) Bremek Alcohol Extract. Journal of Chinese Medicinal Materials (09), 961-963. <https://doi.org/10.13863/j.issn1001-4454.2006.09.032.>

Ran, Y., Chen, Z., Sacramento, C.Q., Fan, L., Cui, Q., Rong, L., Du, R., 2025. Scutellaria barbata D. Don extracts alleviate SARS-CoV-2 induced acute lung injury by inhibiting virus replication and bi-directional immune modulation. Virol. Sin. 40 (3), 430-438. <https://doi.org/10.1016/j.virs.2025.04.004.>

Shao, P., Dong, S., Mu, L.T., Han, L., Lv, C.N., Yuan, J.Z., Lu, J.C., 2024. Two new anti-inflammatory compounds from the roots and rhizomes of Clematis terniflora var. manshurica (Rupr.) Ohwi. Nat. Prod. Res. 38 (11), 1874-1881. <https://doi.org/10.1080/14786419.2023.2227912.>

Shen, Y., Wen, L., Zhang, R., Wei, Z., Shi, N., Xiong, Q., Xia, Q., Xing, Z., Zeng, Z., Niu, H., Huang, W., 2018. Dihydrodiosgenin protects against experimental acute pancreatitis and associated lung injury through mitochondrial protection and PI3Kγ/Akt inhibition. Br. J. Pharmacol. 175 (10), 1621-1636. <https://doi.org/10.1111/bph.14169.>

Shuang, L., Dongyang, X., Minchang, X., Gang, D., Hua, Z., 2019. Extraction the Volatile Oil from Yao Medicine Asplenium prolongatum Hook.and Study on Its Antiinflammatory and Analgesic Activities. Traditional Chinese Drug Research and Clinical Pharmacology 30 (10), 1172-1177. <https://doi.org/10.19378/j.issn.1003-9783.2019.10.004.>

Sun, Z., Li, Y., Zhong, R., and Li, R. (2023c). Hypericum sampsonii Hance: a review of its botany, traditional uses, phytochemistry, biological activity, and safety. *Front. Pharmacol.* 14 1247675. doi:10.3389/fphar.2023.1247675

Szekalska, M., Sosnowska, K., Tomczykowa, M., Winnicka, K., Kasacka, I., Tomczyk, M., 2020. In vivo anti-inflammatory and anti-allergic activities of cynaroside evaluated by using hydrogel formulations. Biomedicine & pharmacotherapy = Biomedecine & pharmacotherapie 121, 109681. <https://doi.org/10.1016/j.biopha.2019.109681.>

Tao, J., Wei, Y., Hu, T., 2016. Flavonoids of Polygonum hydropiper L. attenuates lipopolysaccharide-induced inflammatory injury via suppressing phosphorylation in MAPKs pathways. BMC Complement. Altern. Med. 16, 25. <https://doi.org/10.1186/s12906-016-1001-8.>

Tian, S., Xin, G., Zhang, K., Wen, A., Yu, X., Ni, C., Liu, Y., Li, X., Cao, Y., Huang, W., 2025. Eriodictyol from Scutellariae Barbata alleviates inflammation and necrosis via ZBP1-dependent signaling in acute pancreatitis. Phytomedicine 145, 156926. <https://doi.org/10.1016/j.phymed.2025.156926.>

Tsang, S.W., Zhang, H.J., Chen, Y.G., Auyeung, K.K., Bian, Z.X., 2015. Eruberin A, a Natural Flavanol Glycoside, Exerts Anti-Fibrotic Action on Pancreatic Stellate Cells. Cellular physiology and biochemistry : international journal of experimental cellular physiology, biochemistry, and pharmacology 36 (6), 2433-2446. <https://doi.org/10.1159/000430204>

Wang, A., Xia, Y., Wang, H., Chang, H., Luo, D., Wu, S., et al. (2025a). Aesculi semen (Aesculus chinensis Bunge and Aesculus wilsonii Rehd): A review of its phytochemistry, pharmacology, clinical application and toxicity. *Fitoterapia* 185 106679. doi:10.1016/j.fitote.2025.106679

Wang, B. L., Ge, Z. K., Qiu, J. R., Luan, S. Q., Hao, X. C., and Zhao, Y. H. (2024a). Sedum aizoon L.: a review of its history, traditional uses, nutritional value, botany, phytochemistry, pharmacology, toxicology, and quality control. *Front. Pharmacol.* 15 1349032. doi:10.3389/fphar.2024.1349032

Wang, J., Chen, L., Qu, L., Li, K., Zhao, Y., Wang, Z., Li, Y., Zhang, X., Jin, Y., Liang, X., 2019. Isolation and bioactive evaluation of flavonoid glycosides from Lobelia chinensis Lour using two-dimensional liquid chromatography combined with label-free cell phenotypic assays. J Chromatogr A 1601, 224-231. <https://doi.org/10.1016/j.chroma.2019.04.073.>

Wang, M., Li, Y., Su, J., Bai, J., Zhao, Z., Sun, Z., 2023. Protective effects of 4-geranyloxy-2,6-dihydroxybenzophenonel on DSS-induced ulcerative colitis in mice via regulation of cAMP/PKA/CREB and NF-κB signaling pathways. Phytother Res 37 (4), 1330-1345. <https://doi.org/10.1002/ptr.7689.>

Wang, M., Peng, X., Yang, C., Yu, X., Tai, H., and Li, Y. (2025e). Enhanced therapeutic efficacy of Dioscorea cirrhosa Lour. n-butanol fractions in ulcerative colitis: a dual administration approach with comprehensive mucosal protection and anti-inflammatory effects. *Nat. Prod. Res.* 1-11. doi:10.1080/14786419.2025.2544320

Wang, M., Wang, S., Hu, W., Wang, Z., Yang, B., and Kuang, H. (2022b). Asparagus cochinchinensis: A review of its botany, traditional uses, phytochemistry, pharmacology, and applications. *Front. Pharmacol.* 13 1068858. doi:10.3389/fphar.2022.1068858

Wu, B., Zheng, R., Ouyang, M., Zhu, Y., Lu, H., Liao, K., et al. (2024a). The water extract of Amydrium sinense (Engl.) H. Li ameliorates Isoproterenol-induced cardiac hypertrophy through inhibiting the NF-κB signaling pathway. *Biomedicine & pharmacotherapy = Biomedecine & pharmacotherapie* 172 116241. doi:10.1016/j.biopha.2024.116241

Wu, W., Qu, X., Hu, C., Zhu, X., Wan, M., Zhou, Y., et al. (2024c). Gypenoside LXXV Alleviates Colitis by Reprograming Macrophage Polarization via the Glucocorticoid Receptor Pathway. *J. Agric. Food Chem.* 72 (37), 20444-20457. doi:10.1021/acs.jafc.4c04784

Wu, X., Xu, N., Ye, Z., Zhao, Q., Liu, J., Li, J., Wu, M., Zheng, Y., Li, X., Li, W., Zhang, T., Hu, X., Zhang, Q., 2022. Polysaccharide from Scutellaria barbata D. Don attenuates inflammatory response and microbial dysbiosis in ulcerative colitis mice. Int. J. Biol. Macromol 206, 1-9. <https://doi.org/10.1016/j.ijbiomac.2022.02.119.>

Wu, Z., Huang, B., Zhang, D., Yu, Q., Yan, C., 2025. Structural characterization of a pectin-like polysaccharide from Clematis chinensis Osbeck and its anti-rheumatoid arthritis activity. Int. J. Biol. Macromol 307 (Pt 4), 141917. <https://doi.org/10.1016/j.ijbiomac.2025.141917.>

Xie, P., Luo, H.T., Pei, W.J., Xiao, M.Y., Li, F.F., Gu, Y.L., Piao, X.L., 2024. Saponins derived from Gynostemma pentaphyllum regulate triglyceride and cholesterol metabolism and the mechanisms: A review. J. Ethnopharmacol. 319 (Pt 1), 117186. <https://doi.org/10.1016/j.jep.2023.117186.>

Xing, F., Su, H.Y., Zhong, H.Y., Li, Y.Z., Zhang, Y.Y., Chen, L., Zhou, X.L., 2025. Synthesis and biological evaluation of lappaconitine analogues as potential anti-neuroinflammatory agents by side chain modification and scaffold hopping strategy. Biorg. Med. Chem. 117, 118012. <https://doi.org/10.1016/j.bmc.2024.118012.>

Xiong, Y., Ma, Y., Han, W., Kodithuwakku, N.D., Liu, L.F., Li, F.W., Fang, W.R., Li, Y.M., 2014. Clematichinenoside AR induces immunosuppression involving Treg cells in Peyer׳s patches of rats with adjuvant induced arthritis. J. Ethnopharmacol. 155 (2), 1306-1314. <https://doi.org/10.1016/j.jep.2014.07.028.>

Xue, Z., Cao, Z., Jin, M., Zhang, X., Wang, X., Dou, J., Zhu, Y., Ito, Y., Guo, Z., 2021. New steroid saponins from Dioscorea zingiberensis yam and their medicinal use against I/R via anti-inflammatory effect. Food Funct. 12 (18), 8314-8325. <https://doi.org/10.1039/d1fo01301g.>

Yan, J., Xu, X., Zhu, Y., Wang, Y., Duan, X., 2025. Escin Ia ameliorates DSS-induced chronic colitis in mice by inhibiting inflammation and oxidative stress via the LOXL2/MMP-9 pathway. J. Ethnopharmacol. 345, 119623. <https://doi.org/10.1016/j.jep.2025.119623.>

Yang, Y., Hyun Moh, S., Yu, T., Gwang Park, J., Hyo Yoon, D., Woong Kim, T., Hwan Kim, S., Lee, S., Hong, S., Youl Cho, J., 2012. Methanol extract of Osbeckia stellata suppresses lipopolysaccharide- and HCl/ethanol-induced inflammatory responses by inhibiting Src/Syk and IRAK1. J. Ethnopharmacol. 143 (3), 876-883. <https://doi.org/10.1016/j.jep.2012.08.015.>

Ye, Z., Ge, Z., Yang, S., Hu, T., Ye, Q., Chen, H., 2024. Scutellarein alleviates osteoarthritis progression through the PI3K/Akt/NF-kappaB signaling pathway: In vitro and in vivo studies. Phytother Res 38 (7), 3509-3524. <https://doi.org/10.1002/ptr.8232.>

Ying-Ying, Y., Xin-Huan, W., Ying-Nan, L., Ling-Yan, G., Chang-Zheng, Z., 2020. [Research progress on chemical constituents and pharmacological effects of Sedum sarmentosum]. Zhongguo Zhong yao za zhi = Zhongguo zhongyao zazhi = China journal of Chinese materia medica 45 (18), 4341-4348. <https://doi.org/10.19540/j.cnki.cjcmm.20200623.601.>

Yoo, S.R., Ha, H., Shin, H.K., Seo, C.S., 2020. Anti-Inflamatory Activity of Neolignan Compound Isolated from the Roots of Saururus chinensis. Plants (Basel, Switzerland) 9 (8) . <https://doi.org/10.3390/plants9080932.>

Yue, J., Zhang, N., Xu, T., Wang, J., Cai, B., Yu, Y., 2022. Phenylpropanoid Derivatives from the Tuber of Asparagus cochinchinensis with Anti-Inflammatory Activities. Molecules 27 (22) . <https://doi.org/10.3390/molecules27227676.>

Yulan, C., Ya, T.U., Liming, B., Meirong, B., 2025. Research Progress on the Chemical Components and Pharmacological Effects and Quality Marker Prediction of Bistortae Rhizoma. Information on Traditional Chinese Medicine 42 (01), 76-83. <https://doi.org/10.19656/j.cnki.1002-2406.20250113.>

YUlan, L., Yi, S., Chunxing, H.U., Mei, L., Min, Z., Jinshuang, X.U., Huifang, C., 2021. Summary of the Pharmacological Activities of Miao Medicine Polygonum capitatum. Journal of Guizhou University of Traditional Chinese Medicine 43 (01), 81-84. <https://doi.org/10.16588/j.cnki.issn2096-8426.2021.01.021.>

Zhang, C. L., Zhang, J. J., Zhu, Q. F., Guan, H. Y., Yang, Y. X., He, X., et al. (2021a). Antihyperuricemia and antigouty arthritis effects of Persicaria capitata herba in mice. *Phytomedicine* 93 153765. doi:10.1016/j.phymed.2021.153765

Zhang, J., Rho, Y., Kim, M. Y., and Cho, J. Y. (2021b). TAK1 in the AP-1 pathway is a critical target of Saururus chinensis (Lour.) Baill in its anti-inflammatory action. *J. Ethnopharmacol.* 279 114400. doi:10.1016/j.jep.2021.114400

Zhang, L., Miao, X., Li, Y., Dai, H., Shang, X., Hu, F., et al. (2020a). Toxic and active material basis of Aconitum sinomontanum Nakai based on biological activity guidance and UPLC-Q/TOF-MS technology. *J. Pharm. Biomed. Anal.* 188 113374. doi:10.1016/j.jpba.2020.113374

Zhang, L., Zhu, X. Z., Badamjav, R., Zhang, J. Z., Kou, J. P., Yu, B. Y., et al. (2022b). Isoorientin protects lipopolysaccharide-induced acute lung injury in mice via modulating Keap1/Nrf2-HO-1 and NLRP3 inflammasome pathways. *Eur. J. Pharmacol.* 917 174748. doi:10.1016/j.ejphar.2022.17474

Zhang, P., Yu, L., Cao, H., Ruan, J., Li, F., Wu, L., Zhang, Y., Wang, T., 2024. Potential Anti-Inflammatory Constituents from Aesculus wilsonii Seeds. Molecules 29 (5) . <https://doi.org/10.3390/molecules29051136.>

Zhang, S., Huang, J., Xie, X., He, Y., Mo, F., Luo, Z., 2017. Quercetin from Polygonum capitatum Protects against Gastric Inflammation and Apoptosis Associated with Helicobacter pylori Infection by Affecting the Levels of p38MAPK, BCL-2 and BAX. Molecules 22 (5), 744. <https://doi.org/10.3390/molecules22050744.>

Zhang, S., Nie, H., Yang, Y., Yang, L., and He, J. (2023a). Activating Blood Circulation, Anti-Inflammatory and Diuretic Effects of Leonurus japonicus Extract on a Rat Model of Trauma Blood Stasis and Its Phytochemical Profiling. *Chem. Biodivers.* 20 (3), e202201176. doi:10.1002/cbdv.202201176

Zhang, X., Jin, M., Tadesse, N., Dang, J., Zhou, T., Zhang, H., et al. (2018a). Dioscorea zingiberensis C. H. Wright: An overview on its traditional use, phytochemistry, pharmacology, clinical applications, quality control, and toxicity. *J. Ethnopharmacol.* 220 283-293. doi:10.1016/j.jep.2018.03.017

Zhang, Y., Tian, Z., Wan, H., Liu, W., Kong, F., and Ma, G. (2020c). Deltonin Ameliorates Cerebral Ischemia/Reperfusion Injury in Correlation with Modulation of Autophagy and Inflammation. *Neuropsychiatr. Dis. Treat.* 16 871-879. doi:10.2147/NDT.S227988

Zhang, Y., Tu, X., Zhang, Y., Wen, D., Zhao, F., Yuan, L., et al. (2023b). [Anti-inflammatory mechanism of Balanophora involucrata: a network pharmacology and molecular docking-based analysis and verification in lipopolysaccharide-induced RAW264.7 cells]. *Nan fang yi ke da xue xue bao = Journal of Southern Medical University* 43 (3), 383-392. doi:10.12122/j.issn.1673-4254.2023.03.07

Zhong, J., Wu, X., Huang, C., Li, Y., Huang, M., Xu, L., Lu, J., Pang, L., Huang, Q., Chen, J., 2025. Network Pharmacology and Experimental Validation to Reveal the Pharmacological Mechanisms of Gynostemma pentaphylla against Acute Pharyngitis. Curr. Comput. Aided Drug Des. . <https://doi.org/10.2174/0115734099324793250116133159.>

Zhou, J., Du, S.Y., Fang, Z.Y., Zeng, Z., 2021. New butenolides with anti-inflammatory activity from Balanophora fungosa. Nat. Prod. Res. 35 (11), 1825-1829. <https://doi.org/10.1080/14786419.2019.1645663.>

Zhu, F., Xi, T., Liu, Q., Yu, N., Wang, F., Wen, Y., Wang, W., 2025. Asparagus polysaccharide attenuates atherosclerosis by modulating gut microbiota and metabolites. Int. J. Biol. Macromol 319 (Pt 3), 145492. <https://doi.org/10.1016/j.ijbiomac.2025.145492.>
