# Supplementary material for: Comprehensive review of tujia “Lian” medicinal botanical drugs: traditional classification system, phytochemical, and pharmacological profile
Source: Front Pharmacol. 2026 Feb 18;17:1747999. doi: 10.3389/fphar.2026.1747999 (PMC12957786; doi:10.3389/fphar.2026.1747999)
Supplement: Supplementary file 3 [file Table3.docx]

**SUPPLEMENTARY TABLE 3 "Lian" drugs and their anti-tumor effects.**

| **Sr. No.** | **"Lian" drugs**  **name** | **Source** | **Study** | **Model/Assay** | **Conc. /Dose range** | **Effective sites or metabolites** |
| --- | --- | --- | --- | --- | --- | --- |
|  | Chuanxinlian | *Aconitum sinomontanum* Nakai | *In vitro/*  *In vivo* | Various human cancer cell lines, Cytotoxicity assays, CDX, S180 solid tumor allograft model | 0.1-100 *µ*M | Lappaconitine sulfate, Lappaconitine hydrobromide (Ma et al., 2023); Lappaconitine (Zhang et al., 2024c); Diterpenoid alkaloids (Li et al., 2021); Sinomontanine N, Lappaconitine hydrobromide (Chunyan et al., 2022) |
|  | Huoxuelian | *Adenocaulon himalaicum* Edgew. | *In vitro* | Various human cancer cell lines, Cytotoxicity assays, Murine hepatoma detoxification model, MTT assay, Annexin V-FITC/PI assay, WB analysis, Flow cytometry | 1-100 *µ*g/mL | Methylene chloride fraction, Ethyl acetate fraction (Yun et al., 2013) |
|  | Qiyelian | *Aesculus chinensis* Bunge and *Aesculus chinensis* *var. wilsonii* (Rehder) Turland & N. H. Xia | *In vitro/ In vivo* | Various human cancer cell lines, Cytotoxicity assays, CDX | 5-100 *µ*M, 5-25 mg/kg/week | Triterpenoid saponin (Cheng et al., 2018); Escin (Wang et al., 2012c) |
|  | Xinanyinhualian | *Anemone davidii* Franch. | *In vitro/*  *In vivo* | CDX, ACC-M cells, H460 cells | 1-16 *µ*g/mL, 5-10 mg/kg/day | Raddeanin A, Total alkaloids, Saponins (Yu et al., 2019) |
|  | Dengtailian | *Arisaema heterophyllum* Blume*, Arisaema erubescens* (Wall.) Schott and *Arisaema amurense* Maxim. | *In vitro/*  *In vivo* | Various human cancer cell lines, CDX, Cytotoxicity assays | 10-160 *µ*M. 25-1000*µ*g/mL, 100 mg/kg/day | Agglutinin (Feng et al., 2016); Stigmasterol (Song et al., 2022a); Extracts, Polysaccharide (Lianrui and Mingsan, 2023) |
|  | Baierlian | *Asparagus cochinchinensis* (Lour.) Merr. | *In vitro/*  *In vivo* | Walker-256 rat hepatoma model, Various human cancer cell lines | 50-100 *µ*M, 200 mg/kg/day | polysaccharide (Weng et al., 2014); Furostanol saponins (Wang et al., 2022b) |
|  | Baierlian | *Asparagus densiflorus* (Kunth) Jessop | *In vitro* | MCF-7 cells, HepG2 cells | 10-200 *µ*g/mL | Methanolic extract (Mady et al., 2024) |
|  | Dierlian | *Asparagus filicinus* D. Don | *In vitro/*  *In vivo* | Various human cancer cell lines, Cytotoxicity assays, CDX, MTT assay | 1-28.90 *µ*M, 5-50 mg/kg | Steroidal saponins (Zhao et al., 2018); 25*S*-Spirostanol glycoside (Wu et al., 2010); Aspafilioside B (Liu et al., 2016) |
|  | Guanyinlian | *Balanophora involucrata* Hook. f. | *In vitro* | Enzymatic inhibitory activity | 1-40*µ*M | Polysaccharide (Chao et al., 2024) |
|  | Daosilian | *Bistorta amplexicaulis subsp. sinensis* (F. B. Forbes & Hemsl. ex Steward) Soják | *In vitro/*  *In vivo* | SMMC-7721 cells, HepG2 cells, MDA-MB-231 cells, MCF-7 cells, CDX | 5-40 *µ*M, 25-400 *µ*g/mL, 20 mg/kg | Total flavonoids (Xiang et al., 2015); Amplexicaule A (Xiang et al., 2016) |
|  | Huoxuelian | *Bistorta officinalis* Raf. | *In vitro* | Various human cancer cell lines, Cytotoxicity assays, | 30 *µ*M, 6.25-100 *µ*g/mL, 0.5-4 mg/mL | MeOH-H_2_O extract (Intisar et al., 2012); *β*-sitosterol (Manoharan et al., 2007); Aqueous extract (Liu et al., 2017); Phenolic metabolites (Yulan et al., 2025) |
|  | Tiexianlian | *Clematis chinensis* Osbeck | *In vitro* | Various human cancer cell lines, Cytotoxicity assays, Antiproliferative assays | 40 *µ*M, 50-100 *µ*g/mL | Oleanolic Acid, Quercetin (Chen et al., 2016); Triterpenoid saponin (Lin et al., 2021) |
|  | Babaolian | *Clerodendrum bungei* Steud. | *In vitro/*  *In vivo* | Cytotoxicity assay against KB cells, Syngeneic tumor model, Immunohistochemistry analysis, HepG2 cells, MTT assay, WB analysis, Annexin V-FITC/PI assay, PI flow cytometry | 40-60 *µ*M, 20-200 *µ*g/mL, 2-10 g/kg | 16-Hydroxyclerod-3,13-dien-15,16-olide, Clerodin, Clerodendrin B (Liu et al., 2008); Ethanol extract, Total flavonoids (He et al., 2022b) |
|  | Yeshulian | *Dioscorea polystachya* Turcz. | *In vitro/*  *In vivo* | Network pharmacology, Urethane-induced lung cancer model | 0.5-8 *µ*M, 20 -40 mg/kg/day | Dioscin (Xi et al., 2022); Diosgenin (Kumar et al., 2025) |
|  | Guanyinlian | *Dioscorea zingiberensis* C. H. Wright | *In vitro/*  *In vivo* | Various human cancer cell lines, CDX | 28.40-31.20 *µ*M, 15 mg/kg/day | Spirostanol-type steroidal saponin (Liu et al., 2021b); Deltonin (Yang et al., 2023b) |
|  | Bajiaolian | *Dysosma versipellis* (Hance) M. Cheng | *In vitro/*  *In vivo* | NOD/SCID, Limiting dilution assay, Various human cancer cell lines, Cytotoxicity assays, MTT assay, Zebrafish xenograft model | 0.1-100 *µ*M, 10-100 *µ*g/mL, 50 mg/kg/day | Quercetin (Nwaeburu et al., 2017); Lignans (Sun et al., 2023a); Fungal strains (Tan et al., 2018); Podophyllotoxin, 4'-Demethylpodophyllotoxin, Diphyllin (Yang et al., 2013); Podophyllotoxone, α-Peltatin, β-Peltatin (Xu et al., 2011) |
|  | Mohanlian | *Eclipta prostrata* (L.) L. | *In vitro/*  *In vivo* | Various human cancer cell lines, CCK-8 assay, Wound healing (scratch) assay, Transwell invasion assay, Annexin V-FITC/PI assay, PI flow cytometry, WB analysis, DLA ascites model, CDX | 5-80 *µ*M, 15.625-500 *µ*g/mL, 200-400 mg/kg | Wedelolactone (Zou et al., 2022);  Extract, Hydro-alcoholic extract, Chloroform fraction (Feng et al., 2019); Methanol extract (Timalsina and Devkota, 2021) |
|  | Yinxianlian | *Goodyera schlechtendaliana* Rchb. f. | *In vitro* | Enzymatic inhibitory activity | 8.26 ± 0.52 *µ*M | Goodyschle A (Dai et al., 2021) |
|  | Qiyelian | *Gynostemma pentaphyllum* (Thunb.) Makino | *In vitro/*  *In vivo* | Data source models, Network pharmacology analysis, Molecular docking, Molecular dynamics simulations, proposed biological mechanism model, MKN-45 cells, HGC-27 cells, CCK-8 assay, Annexin V-FITC/PI assay, PI flow cytometry, WB analysis, T-cell Killing Assay, Syngeneic tumor model, Various human cancer cell lines, Transwell chamber assay, CDX | 10-160 *µ*M, 10-20 mg/kg/day | Quercetin, Luteolin, Gypenoside XLIX (Zhang et al., 2024d); Saponin metabolites (Liu et al., 2025g); Gypenoside (Wu et al., 2024b); Flavonoids and saponins (Xie et al., 2024); Polysaccharides (Hu et al., 2025); Gypenoside LI (Liu et al., 2025c); Gypenoside L (Xiao et al., 2024) |
|  | Baiweilian | *Hemsleya chinensis* Cogn. ex F. B. Forbes & Hemsl. | *In vitro* | Mechanistic assays, Cytotoxicity assays, Various human cancer cell lines | 10-100 nM, 0.78-100 *µ*M | Curcurbitacin IIa (Li et al., 2018a); Polyhydroxy cucurbitane-type triterpenoids (Feng et al., 2025) |
|  | Honghanlian | *Hypericum ascyron* L. | *In vitro/*  *In vivo* | Various human cancer cell lines, Cytotoxicity assays, S180 solid tumor allograft model | 1-40 *µ*M, 72.70 *µ*g/mL, 25-100 mg/kg/day | Betulinic acid,3-α-hydroxylup-20(29)-en-28-oic acid (Li et al., 2018b); Spirocyclic polycyclic polyprenylated acylphloroglucinols (Hu et al., 2023); 2,3,4-nor-polycyclic polyprenylated acylphloroglucinols (Hu et al., 2019); Total flavonoids (Li et al., 2015b); Tomoeone F (Hashida et al., 2008) |
|  | Duiyuelian | *Hypericum sampsonii* Hance | *In vitro/*  *In vivo* | Mechanistic assays, Cytotoxicity assays, MTT assay, Annexin V-FITC/PI assay, TUNEL assay, DAPI staining, WB analysis, CDX, Various human cancer cell lines, Flow cytometry, CCK-8 assay | 0.1-100 *µ*M, 5-20 *µ*g/mL, 100 mg/kg/day | Chloroform-soluble fraction (Zeng et al., 2006); Phloroglucinol derivatives, Xanthone derivatives (Chen et al., 2020); Prenylated phloroglucinol derivatives (Xin et al., 2012); Polyprenylated acylphloroglucinols (Wang et al., 2025f); 7-Epiclusianone, Sampsonione I, Mangiferin, Naringenin, Quercetin, and Rutin (Sun et al., 2023c) |
|  | Yimulian | *Leonurus japonicus* Houtt. | *In vitro* | Dendritic cell maturation model, Antigen uptake and presentation model, T-cell activation model, Mechanistic model | 1-100 *µ*M | Leonurine (Chen et al., 2023) |
|  | Banbianlian | *Lobelia chinensis* Lour. | *In vitro/*  *In vivo* | CDX, DMH-induced ACF model, Various human cancer cell lines, Cytotoxicity assays | 1-200 *µ*g/mL, 50 mg/kg, 100-200 mg/kg/day, 40 mg/kg/two days, 1-4 g/kg | Ethanol extract (Luo et al., 2024); Lobetyol (Shen et al., 2016); Alkaloids; Kaempferol-3-*O*-glucoside; Extract (Chen et al., 2014); Flavonoids, Coumarins, Phenolic acids (Yang et al., 2014); Aqueous extract (Han et al., 2013); Hot water extract (Santosa et al., 1986) |
|  | Dabanbianlian | *Lobelia davidii* Franch. | *In vitro* | MTT assay, A549 cells | 26.72 ± 2.05 *µ*M | Triterpenoid (Juelin, 2024) |
|  | Qiyelian | *Paris polyphylla var. Chinensis* (Franch.) Hara | *In vitro/*  *In vivo* | Various human cancer cell lines, Cytotoxicity assays, Immunomodulatory antitumor model, MTT assay, Hoechst 33258 staining, Annexin V-FITC/PI assay, JC-1 staining, WB analysis, S180 ascites model | 1-16 *µ*M, 25-100 mg/kg, 50-400 mg/kg/day | Steroidal saponins (Li et al., 2013); Polysaccharide (Song et al., 2025); Homo-aro-cholestane glycoside (Guan et al., 2021) |
|  | Yanqiaolian | *Persicaria capitata* (Buch. Ham. ex D. Don) H. Gross | *In vitro* | Various human cancer cell lines, CCK-8 assay | 1-40 *µ*M | Ethanol extracts, Davidin (Hooper and Gordon, 2001; Neish, 2009), Phenolic metabolite (He et al., 2025a), Extract (YUlan et al., 2021) |
|  | Qiaokelian | *Persicaria chinensis* (L.) H. Gross | *In vitro* | SiHa cells, HCT-116 cells | 6.25-100 *µ*M, 25-200 *µ*g/mL | Phenolic acids (Chen et al., 2020); Extract (Fengfeng and Hua, 2025) |
|  | Guanyinzuolian | *Phedimus aizoon* (L.) 't Hart | *In vitro/*  *In vivo* | Preliminary cytotoxicity screening, HepG2 cells, MTT assay, Syngeneic tumor model | 40 *µ*M, 50-200 *µ*g/mL, 200-800 mg/kg | Flavonoid glycosides (Xu et al., 2015); Ethanol extracts, Total Flavonoids, Aizoon (Wang et al., 2024a) |
|  | Luanjiaolian | *Pholidota yunnanensis* Rolfe | *In vitro* | HepG2 cells | 6.25-100 *µ*M | Phoyunbene B (Wang et al., 2012a) |
|  | Dujiaolian | *Pinellia pedatisecta* Schott | *In vitro/*  *In vivo* | TC-1 tumor model, Various human cancer cell lines, Macrophage phagocytosis assay, Mouse clearance model | 10-40 *µ*M, 0.625-100 *µ*g/mL, 50 mg/kg/day | Lipid-soluble extract (Huang et al., 2018); Extract (Wang et al., 2024b); Alkaloids (DU et al., 2018); Pinellia pedatisecta agglutinin (Chen et al., 2013) |
|  | Xionghuanglian | *Pleuropterus ciliinervis* Nakai | *In vitro* | Histone Deacetylase (HDAC) enzyme activity, COLO 205 cells | 8.30-129.40 *µ*M | Bis(4-hydroxybenzyl) sulfide (Son et al., 2007) |
|  | Jixuelian | *Pronephrium penangianum* (Hook.) Holttum | *In vitro* | Various human cancer cell lines, Cytotoxicity assays | 10-40 *µ*M | Flavan-4-ol glycosides (Zhao et al., 2006) |
|  | Fengkelian | *Prunella asiatica* Nakai | *In vitro/*  *In vivo* | Syngeneic tumor model, Human lung cancer A549 cells and mouse lung cancer LLC (Lewis lung carcinoma) cells, MTT assay, Hoechst 33258 staining, Annexin V-FITC/PI assay, Flow cytometry | 6.25-100 *µ*g/mL, 50mg/kg/day | Ursolic acid, Oleanolic acid, Rosmarinic acid (Feng et al., 2010) |
|  | Runxuelian | *Pyrola calliantha* Andres | *In vitro* | Doxorubicin-resistant MG63 cells, Human chondrosarcoma SW1353 cells, HeLa human cervical adenocarcinoma cells | 8.41 ± 0.72 *µ*M, 9.85 ± 0.91 *µ*M, 6.25-100 *µ*g/mL | Chimaphilin (Daqian et al., 2015); Total volatile oil (Cai et al., 2013); Extract (He et al., 2022a) |
|  | Matilian | *Rheum* *palmatum* L. | *In vitro/*  *In vivo* | ³H-Thymidine Incorporation Assay, S180 solid tumor allograft model, MTT assay | 50-200 *µ*g/mL, 25-100 mg/kg/day | Chrysophanol (Liu et al., 2025b); Rhein (Wang et al., 2024f); Polysaccharide (Du Yun et al., 2022) |
|  | Dujiaolian | *Sauromatum giganteum* (Engl.) Cusimano & Hett. | *In vitro/*  *In vivo* | Various human cancer cell lines, Serum pharmacology, Cytotoxicity assays | 79.20 *µ*g/mL, 30 g/kg | Ethyl acetate fraction (Gao et al., 2015); Supercritical fluid CO2 extract (Li et al., 2011) |
|  | Baihelian | *Saururus chinensis* (Lour.) Baill. | *In vitro/*  *In vivo* | Various human cancer cell lines, TGF-*β*-Induced EMT model, BCSC model, MDA-MB-231 parental vs. CD44+/CD24- sorted xenograft model, CCK-8 assay, MTT assay, Wound healing (scratch) assay, Transwell invasion assay, Osteolysis assay, Molecular Docking | 1-100*µ*M, 20 mg/kg/week | Sauchinone (He et al., 2018); Machilin D (Zhen et al., 2020); Saucerneol (Yun et al., 2025); Ent-Sauchinone (He et al., 2025b); Extract (Eun et al., 2024); Tetrahydrofurofuranoid lignin (Yun et al., 2023) |
|  | Banzhilian | *Scutellaria barbata* D. Don | *In vitro/*  *In vivo* | Various human cancer cell lines, CDX, Chemically-Induced Models, Orthotopic Models, Syngeneic Models, CCK-8 assay, Annexin V-FITC/PI assay, PI flow cytometry, Transwell chamber assay, EMT, WB analysis, ROS detection, JC-1 staining, Data Source Models | 1-160*µ*M, 50-1000*µ*g/mL, 5-50 mg/kg, 5-500 mg/kg/day | Flavonoids, Neo-clerodane diterpenoids (Sun et al., 2024); Polysaccharide (Sun et al., 2017); Scutellarin (Ma et al., 2024b); Scutebarbatine B (Niu et al., 2025); Luteolin, Quercetin, Scutellarin (Cao et al., 2025); Extracts (Wang et al., 2025b); Luteolin (Yang et al., 2025a); Salvigenin (Shao et al., 2023); Rhamnazin, Eriodictyol (Yang et al., 2022); Chloroform extract (Xue et al., 2022); Acidic Polysaccharide (Su et al., 2022) |
|  | Gouyabanzhilian | *Sedum sarmentosum* Bunge | *In vitro/*  *In vivo* | CDX, Syngeneic Models, Various human cancer cell lines, Cytotoxicity assays | 10-40 *µ*M, 25-800 *µ*g/mL, 200 mg/kg/day | Ethyl acetate extract (Bai et al., 2016); Arabinogalactoglucan (Zhang et al., 2021e); Megastigmane glucosides (Doan et al., 2022); Total flavonoid fractions, Aqueous extract (Ying-Ying et al., 2020) |
|  | Bagualian | *Sinopodophyllum hexandrum* (Royle) T. S. Ying | *In vitro* | Various human cancer cell lines | 7.80-10.50 nM, 0.12-1.64 *µ*M | podophyllotoxin and its derivatives (Oh et al., 2021); VP-16, VM-26 (Zhang et al., 2018b; Xiao et al., 2020); Aryltetralin lignan (-)-podophyllotoxin (Decembrino et al., 2021); 4-Demethyl-picropodophyllotoxin 7'-*O*-*β*-D-glucopyranoside (Zhang et al., 2005) |
|  | Bixuelian | *Stephania japonica* (Thunb.) Miers | *In vitro* | Various human cancer cell lines | 8.85-10.20 *µ*M | Cepharanthine (Chen et al., 2024a) |
|  | Dayemaweilian | *Thalictrum acutifolium* (Hand. Mazz.) B. Boivin | *In vitro* | PLA-801 cells, 95-D cells | 0.10-20.00 *µ*g/mL | Tangsongcao A (Chen et al., 2002b); Acutiaporberine (Chen et al., 2002a) |

**References**

Bai, Y., Chen, B., Hong, W., Liang, Y., Zhou, M., Zhou, L., 2016. Sedum sarmentosum Bunge extract induces apoptosis and inhibits proliferation in pancreatic cancer cells via the hedgehog signaling pathway. Oncol. Rep. 35 (5), 2775-2784. <https://doi.org/10.3892/or.2016.4679.>

Cai, L., Ye, H., Li, X., Lin, Y., Yu, F., Chen, J., Li, H., Liu, X., 2013. Chemical constituents of volatile oil from Pyrolae herba and antiproliferative activity against SW1353 human chondrosarcoma cells. Int. J. Oncol. 42 (4), 1452-1458. <https://doi.org/10.3892/ijo.2013.1816.>

Cao, H., Song, H., Zhou, W., Lv, X., Liu, X., Xiang, Z., Fu, R., Cheng, Y., Chen, J., Wang, S., Hu, Y., Yan, H., You, W., Guo, C., Chen, B., Cao, G., Wang, W., Jia, J., 2025. Exploring the active ingredients of Banzhilian and its mechanism of action on diabetic Gastric cancer based on network pharmacology. Sci. Rep. 15 (1), 14808. <https://doi.org/10.1038/s41598-025-98214-6.>

Chao, W., Huajun, C., Chenhui, W., Wei, T., Hao, W.U., 2024. Study on the therapeutic mechanism of Balanophora involucrata on drug-induced liver injury by Network pharmacology. West China Journal of Pharmaceutical Sciences 39 (05), 515-521. <https://doi.org/10.13375/j.cnki.wcjps.2024.05.004.>

Chen, B., Chen, L., Yang, J., Hou, M., Cai, Q., Dai, W., et al. (2024a). Cepharanthine inhibits migration, invasion, and EMT of bladder cancer cells by activating the Rap1 signaling pathway in vitro. *American Journal of Translational Research* 16 (5), 1602-1619. doi:10.62347/WDFF743

Chen, C., He, L., Wang, X., Xiao, R., Chen, S., Ye, Z., Wang, X., Wang, Y., Zhu, Y., Dai, J., 2023. Leonurine promotes the maturation of healthy donors and multiple myeloma patients derived-dendritic cells via the regulation on arachidonic acid metabolism. Front. Pharmacol. 14, 1104403. <https://doi.org/10.3389/fphar.2023.1104403.>

Chen, J., Jing, L., Wu-gang, Z., Hui, L., Yu-lin, F., Shi-lin, Y., Ling, Z., 2016. Optimization of Purification Process of Anti-tumor Active Ingredient in Clematis manshurica by Macroporous Resin. Chinese Journal of Experimental Traditional Medical Formulae 22 (15), 14-17.

Chen, K., Yang, X., Wu, L., Yu, M., Li, X., Li, N., Wang, S., Li, G., 2013. Pinellia pedatisecta agglutinin targets drug resistant K562/ADR leukemia cells through binding with sarcolemmal membrane associated protein and enhancing macrophage phagocytosis. PLoS. ONE 8 (9), e74363. <https://doi.org/10.1371/journal.pone.0074363.>

Chen, M.W., Chen, W.R., Zhang, J.M., Long, X.Y., Wang, Y.T., 2014. Lobelia chinensis: chemical constituents and anticancer activity perspective. Chin J Nat Med 12 (2), 103-107. <https://doi.org/10.1016/S1875-5364(14)60016-9.>

Chen, Q., Di, L., Zhang, Y., Li, N., 2020. Chemical constituents with cytotoxic and anti-inflammatory activity in Hypericum sampsonii and the antitumor potential under the view of cancer-related inflammation. J. Ethnopharmacol. 259, 112948. <https://doi.org/10.1016/j.jep.2020.112948.>

Chen, Q., Peng, W., Qi, S., and Xu, A. (2002a). Apoptosis of human highly metastatic lung cancer cell line 95-D induced by acutiaporberine, a novel bisalkaloid derived from Thalictrum acutifolium. *Planta Med.* 68 (6), 550-553. doi:10.1055/s-2002-32546

Chen, Q., Peng, W., and Xu, A. (2002b). Apoptosis of a human non-small cell lung cancer (NSCLC) cell line, PLA-801, induced by acutiaporberine, a novel bisalkaloid derived from Thalictrum acutifolium (Hand.-Mazz.) Boivin. *Biochem. Pharmacol.* 63 (8), 1389-1396. doi:10.1016/s0006-2952(02)00871-7

Chen, W., Shen, X., Ma, L., Chen, R., Yuan, Q., Zheng, Y., Li, C., Peng, G., 2020. Phenolic Compounds from Polygonum chinense Induce Growth Inhibition and Apoptosis of Cervical Cancer SiHa Cells. Biomed Res Int 2020, 8868508. <https://doi.org/10.1155/2020/8868508.>

Chunyan, J., Xiaolou, M., Yunhe, Z., Wenguang, Z., Dan, W., Yun, L.I., 2022. Research Progress on Processing Evolution, Chemical Compositions and Pharmacological Effects of Aconitum sinomontanum Nakai. Chinese Journal of Information on Traditional Chinese Medicine 29 (01), 143-148. <https://doi.org/10.19879/j.cnki.1005-5304.202105299.>

Dai, L.Y., Yin, Q.M., Qiu, J.K., Zhang, Z.Y., Li, G., Huang, M.N., Liu, L., 2021. Goodyschle A, a new butenolide with significant BchE inhibitory activity from Goodyera schlechtendaliana. Nat. Prod. Res. 35 (23), 4916-4921. <https://doi.org/10.1080/14786419.2020.1744142.>

Daqian, W., Chuandong, W., Xinhua, Q., Songtao, A., Kerong, D., 2015. Chimaphilin inhibits proliferation and induces apoptosis in multidrug resistant osteosarcoma cell lines through insulin-like growth factor-I receptor (IGF-IR) signaling. Chem-Biol. Interact. 237, 25-30. <https://doi.org/10.1016/j.cbi.2015.05.008.>

Decembrino, D., Raffaele, A., Knöfel, R., Girhard, M., Urlacher, V.B., 2021. Synthesis of (-)-deoxypodophyllotoxin and (-)-epipodophyllotoxin via a multi-enzyme cascade in E. coli. Microb. Cell Fact. 20 (1), 183. <https://doi.org/10.1186/s12934-021-01673-5.>

Doan, D.X., Sun, S., Omar, A.M., Nguyen, D.T., Hoang, A., Fujiwara, H., Matsumoto, K., Pham, H., Awale, S., 2022. Chemical constituents and absolute configuration of megastigmanes' isolated from Sedum sarmentosum Bunge. Nat. Prod. Res. 36 (9), 2341-2348. <https://doi.org/10.1080/14786419.2020.1834549.>

DU, J., Ding, J., Mu, Z.Q., Guan, S.H., Cheng, C.R., Liu, X., Guo, D.A., 2018. Three new alkaloids isolated from the stem tuber of Pinellia pedatisecta. Chin J Nat Med 16 (2), 139-142. <https://doi.org/10.1016/S1875-5364(18)30040-2.>

Du Yun, Liang, K., Xiangrong, L., Shuhe, K., 2022. General Situation of Extraction Method,Structure and Pharmacological Activity of Rhubarb Polysaccharide. Shandong Chemical Industry 51 (18), 111-114. <https://doi.org/10.19319/j.cnki.issn.1008-021x.2022.18.065.>

Eun, S.Y., Chung, C.H., Cheon, Y.H., Park, G.D., Lee, C.H., Kim, J.Y., Lee, M.S., 2024. Saururus chinensis (Lour.) Baill. extract promotes skeletal muscle cell differentiation by positively regulating mitochondrial biogenesis and AKT/mTOR signaling in vitro. Mol. Med. Report. 30 (1) . <https://doi.org/10.3892/mmr.2024.13250.>

Feng, L., Jia, X.B., Jiang, J., Zhu, M.M., Chen, Y., Tan, X.B., Shi, F., 2010. Combination of active components enhances the efficacy of Prunella in prevention and treatment of lung cancer. Molecules 15 (11), 7893-7906. <https://doi.org/10.3390/molecules15117893.>

Feng, L., Zhai, Y.Y., Xu, J., Yao, W.F., Cao, Y.D., Cheng, F.F., Bao, B.H., Zhang, L., 2019. A review on traditional uses, phytochemistry and pharmacology of Eclipta prostrata (L.) L. J. Ethnopharmacol. 245, 112109. <https://doi.org/10.1016/j.jep.2019.112109.>

Feng, L.X., Sun, P., Mi, T., Liu, M., Liu, W., Yao, S., Cao, Y.M., Yu, X.L., Wu, W.Y., Jiang, B.H., Yang, M., Guo, D.A., Liu, X., 2016. Agglutinin isolated from Arisema heterophyllum Blume induces apoptosis and autophagy in A549 cells through inhibiting PI3K/Akt pathway and inducing ER stress. Chin J Nat Med 14 (11), 856-864. <https://doi.org/10.1016/S1875-5364(16)30102-9.>

Feng, W.B., Liu, M.S., Zheng, T.X., Lin, Z.X., Xu, H.W., Cai, H.T., Zhang, Y.B., Bai, J.L., Yang, M.X., Luo, H., Li, X.S., 2025. New polyhydroxy cucurbitane-type triterpenoids from the tubers of Hemsleya chinensis and their cytotoxic activities. Fitoterapia 186, 106840. <https://doi.org/10.1016/j.fitote.2025.106840.>

Fengfeng, X., Hua, Z., 2025. Research Progress on Chemical Constituents and Pharmacological Effects of Huotanmu(Herba Polygoni Chinensislor). Journal of Liaoning University of Traditional Chinese Medicine 27 (04), 182-192. <https://doi.org/10.13194/j.issn.1673-842X.2025.04.035.>

Gao, S.Y., Gong, Y.F., Sun, Q.J., Bai, J., Wang, L., Fan, Z.Q., Sun, Y., Su, Y.J., Gang, J., Ji, Y.B., 2015. Screening antitumor bioactive fraction from Sauromatum giganteum (Engl.) Cusimano & Hett and sensitive cell lines with the serum pharmacology method and identification by UPLC-TOF-MS. Molecules 20 (3), 4290-4306. <https://doi.org/10.3390/molecules20034290.>

Guan, L.J., Ding, L.S., Li, Y.M., Chen, L.M., Gao, H.M., Wang, Z.M., Wang, Z.J., 2021. A new homo-aro-cholestane glycoside from the rhizome of Paris polyphylla var. chinensis. J. Asian Nat. Prod. Res. 23 (11), 1107-1114. <https://doi.org/10.1080/10286020.2020.1849149.>

Han, S.R., Lv, X.Y., Wang, Y.M., Gong, H., Zhang, C., Tong, A.N., Yan, N., 2013. A study on the effect of aqueous extract of Lobelia chinensis on colon precancerous lesions in rats. African journal of traditional, complementary, and alternative medicines : AJTCAM 10 (6), 422-425. <https://doi.org/10.4314/ajtcam.v10i6.2.>

Hashida, W., Tanaka, N., Kashiwada, Y., Sekiya, M., Ikeshiro, Y., Takaishi, Y., 2008. Tomoeones A-H, cytotoxic phloroglucinol derivatives from Hypericum ascyron. Phytochemistry 69 (11), 2225-2230. <https://doi.org/10.1016/j.phytochem.2008.04.026.>

He, C., Liu, J., Ke, T., Luo, Y., Zhang, S., Mao, T., et al. (2022a). Pyrolae herba: A review on its botany, traditional uses, phytochemistry, pharmacology and quality control. *J. Ethnopharmacol.* 298 115584. doi:10.1016/j.jep.2022.115584

He, L., Zhong, F., Chen, X. J., Yang, Y. R., Yan, X. L., He, M. H., et al. (2025a). A new phenolic compound from Persicaria capitata. *Nat. Prod. Res.* 39 (14), 4097-4103. doi:10.1080/14786419.2024.2332485

He, Y. J., Xiao, D., Gong, Q. Z., Zhu, J. X., Jiang, X. Y., Dai, W. Q., et al. (2025b). Synthesis, biological evaluation, and molecular docking of ent-sauchinone-based amide derivatives with potential anti-invasion and anti-migration activities. *Nat. Prod. Res.* 1-8. doi:10.1080/14786419.2025.2459804

He, Z., Dong, W., Li, Q., Qin, C., Li, Y., 2018. Sauchinone prevents TGF-β-induced EMT and metastasis in gastric cancer cells. Biomedicine & pharmacotherapy = Biomedecine & pharmacotherapie 101, 355-361. <https://doi.org/10.1016/j.biopha.2018.02.121.>

He, Z., Fei, C., You, Q., and Shui-han, Z. (2022b). Research Progress on Chemical Constituents of Clerodendrum bungei Steud. and Its Pharmacological Activities. *Journal of Nanjing University of Traditional Chinese Medicine* 38 (04), 361-374. doi:10.14148/j.issn.1672-0482.2022.0361

Hooper, L.V., Gordon, J.I., 2001. Commensal host-bacterial relationships in the gut. Science 292 (5519), 1115-1118. <https://doi.org/10.1126/science.1058709.>

Hu, X., Wang, W., Zhang, D., Tian, X., Zhang, A., Xu, J., Feng, F., Li, W., Kikuchi, T., Zhang, J., 2025. Phytochemistry, bioactivities and application of Gynostemma pentaphyllum polysaccharide: A review. Int. J. Biol. Macromol 322 (Pt 1), 144964. <https://doi.org/10.1016/j.ijbiomac.2025.144964.>

Hu, Y.L., Hu, K., Kong, L.M., Xia, F., Yang, X.W., Xu, G., 2019. Norascyronones A and B, 2,3,4- nor-Polycyclic Polyprenylated Acylphloroglucinols from Hypericum ascyron. Org. Lett 21 (4), 1007-1010. <https://doi.org/10.1021/acs.orglett.8b04022.>

Hu, Y.L., Yue, G.G., Li, X.R., Xu, G., Lau, C.B., 2023. Structurally diverse spirocyclic polycyclic polyprenylated acylphloroglucinols from Hypericum ascyron Linn. and their anti-tumor activity. Phytochemistry 212, 113727. <https://doi.org/10.1016/j.phytochem.2023.113727.>

Huang, H., Zhang, M., Yao, S., Zhang, M., Peng, J., 2018. Immune modulation of a lipid-soluble extract of Pinellia pedatisecta Schott in the tumor microenvironment of an HPV(+) tumor-burdened mouse model. J. Ethnopharmacol. 225, 103-115. <https://doi.org/10.1016/j.jep.2018.04.037.>

Intisar, A., Zhang, L., Luo, H., Kiazolu, J.B., Zhang, R., Zhang, W., 2012. Anticancer constituents and cytotoxic activity of methanol-water extract of Polygonum bistorta L. African journal of traditional, complementary, and alternative medicines : AJTCAM 10 (1), 53-59. <https://doi.org/10.4314/ajtcam.v10i1.9.>

Juelin, Q. Study on the Chemical Constituents and BiologicalActivities of Lobelia davidii Franch ,2024.

Kumar, A., Amita, Singh, B., Sharma, P., Raina, R., Sharma, B., Haque, S., Tuli, H.S., 2025. Role of diosgenin in gastrointestinal cancers: recent trends and future perspectives. Medical oncology (Northwood, London, England) 42 (9), 397. <https://doi.org/10.1007/s12032-025-02947-3.>

Li, H., Chen, H., Li, R., Xin, J., Wu, S., Lan, J., et al. (2018a). Cucurbitacin I induces cancer cell death through the endoplasmic reticulum stress pathway. *J. Cell Biochem.* . doi:10.1002/jcb.27570

Li, Q., Jiang, C., Zu, Y., Song, Z., Zhang, B., Meng, X., Qiu, W., Zhang, L., 2011. SFE-CO2 extract from Typhonium giganteum Engl. tubers, induces apoptosis in human hepatoma SMMC-7721 cells involvement of a ROS-mediated mitochondrial pathway. Molecules 16 (10), 8228-8242. <https://doi.org/10.3390/molecules16108228.>

Li, X. M., Luo, X. G., He, J. F., Wang, N., Zhou, H., Yang, P. L., et al. (2018b). Induction of apoptosis in human cervical carcinoma HeLa cells by active compounds from Hypericum ascyron L. *Oncol. Lett.* 15 (3), 3944-3950. doi:10.3892/ol.2018.7812

Li, X. M., Luo, X. G., Ma, N., Li, K., Li, W., Ma, D. Y., et al. (2015b). Quality and antitumour activity evaluation of extract of Hypericum ascyron. *Biomed. Chromatogr.* 29 (1), 47-52. doi:10.1002/bmc.3169

Li, Y., Gu, J.F., Zou, X., Wu, J., Zhang, M.H., Jiang, J., Qin, D., Zhou, J.Y., Liu, B.X., Zhu, Y.T., Jia, X.B., Feng, L., Wang, R.P., 2013. The anti-lung cancer activities of steroidal saponins of P. polyphylla Smith var. chinensis (Franch.) Hara through enhanced immunostimulation in experimental Lewis tumor-bearing C57BL/6 mice and induction of apoptosis in the A549 cell line. Molecules 18 (10), 12916-12936. <https://doi.org/10.3390/molecules181012916.>

Li, Y., Zeng, J., Tian, Y.H., Hou, Y., Da, H., Fang, J., Gao, K., 2021. Isolation, identification, and activity evaluation of diterpenoid alkaloids from Aconitum sinomontanum. Phytochemistry 190, 112880. <https://doi.org/10.1016/j.phytochem.2021.112880.>

Lianrui, W., Mingsan, M., 2023. Research Progress of Arisaematis Rhizoma and Predictive Analysis on Its Q-marker. Traditional Chinese Drug Research and Clinical Pharmacology 34 (11), 1640-1647. <https://doi.org/10.19378/j.issn.1003-9783.2023.11.019.>

Lin, T.F., Wang, L., Zhang, Y., Zhang, J.H., Zhou, D.Y., Fang, F., Liu, L., Liu, B., Jiang, Y.Y., 2021. Uses, chemical compositions, pharmacological activities and toxicology of Clematidis Radix et Rhizome- a Review. J. Ethnopharmacol. 270, 113831. <https://doi.org/10.1016/j.jep.2021.113831.>

Liu, C., Qiu, S., Liu, X., Huang, R., and Fang, Z. (2025b). Chrysophanol Attenuates Cardiac Fibrosis and Arrhythmia by Suppressing the Endoplasmic Reticulum Stress/Pyroptosis Axis and Inflammation. *Phytother Res* . doi:10.1002/ptr.8476

Liu, S., Zhu, H., Zhang, S., Zhang, X., Yu, Q., Xuan, L., 2008. Abietane diterpenoids from Clerodendrum bungei. J. Nat. Prod. 71 (5), 755-759. <https://doi.org/10.1021/np0703489.>

Liu, T., Tian, C., Li, H., Zhang, C., Duan, W., Yuan, J., et al. (2025e). Therapeutic potential of inosine in acute lung injury: mechanistic insights into TLR4 suppression and macrophage polarization. *Phytomedicine* 143 156854. doi:10.1016/j.phymed.2025.156854

Liu, W., Ning, R., Chen, R.N., Huang, X.F., Dai, Q.S., Hu, J.H., Wang, Y.W., Wu, L.L., Xiong, J., Hu, G., Guo, Q.L., Yang, J., Wang, H., 2016. Aspafilioside B induces G2/M cell cycle arrest and apoptosis by up-regulating H-Ras and N-Ras via ERK and p38 MAPK signaling pathways in human hepatoma HepG2 cells. Mol. Carcinog. 55 (5), 440-457. <https://doi.org/10.1002/mc.22293.>

Liu, X., Zhou, P., He, K., Wen, Z., and Gao, Y. (2021b). Dioscorea Zingiberensis New Saponin Inhibits the Growth of Hepatocellular Carcinoma by Suppressing the Expression of Long Non-coding RNA TCONS-00026762. *Front. Pharmacol.* 12 678620. doi:10.3389/fphar.2021.678620

Liu, Y., Li, Y., Li, J., Rao, H., Sun, J., Xiu, J., et al. (2025g). Gypenosides alleviate oxidative stress in the hippocampus, promote mitophagy, and mitigate depressive-like behaviors induced by CUMS via SIRT1. *J. Ethnopharmacol.* 337 (Pt 2), 118823. doi:10.1016/j.jep.2024.11882

Liu, Y.H., Weng, Y.P., Lin, H.Y., Tang, S.W., Chen, C.J., Liang, C.J., Ku, C.Y., Lin, J.Y., 2017. Aqueous extract of Polygonum bistorta modulates proteostasis by ROS-induced ER stress in human hepatoma cells. Sci. Rep. 7, 41437. <https://doi.org/10.1038/srep41437.>

Luo, J., Chen, Q.X., Li, P., Yu, H., Yu, L., Lu, J.L., Yin, H.Z., Huang, B.J., Zhang, S.J., 2024. Lobelia chinensis Lour inhibits the progression of hepatocellular carcinoma via the regulation of the PTEN/AKT signaling pathway in vivo and in vitro. J. Ethnopharmacol. 318 (Pt A), 116886. <https://doi.org/10.1016/j.jep.2023.116886.>

Ma, H., Yue, G. G., Lee, J. K., Gao, S., Yuen, K. K., Cheng, W., et al. (2024b). Scutellarin, a flavonoid compound from Scutellaria barbata, suppresses growth of breast cancer stem cells in vitro and in tumor-bearing mice. *Phytomedicine* 128 155418. doi:10.1016/j.phymed.2024.155418

Ma, S., Zheng, Y., Ma, J., Zhang, X., Qu, D., Song, N., Sang, C., Hui, L., 2023. Lappaconitine sulfate inhibits proliferation and induces mitochondrial-mediated apoptosis via regulating PI3K/AKT/GSK3β signaling pathway in HeLa cells. Naunyn Schmiedebergs Arch. Pharmacol. 396 (12), 3695-3705. <https://doi.org/10.1007/s00210-023-02564-9.>

Mady, M.S., Sobhy, Y., Orabi, A., Sharaky, M., Mina, S.A., Abo-Zeid, Y., 2024. Preparation and characterization of nano-emulsion formulations of Asparagus densiflorus root and aerial parts extracts: evaluation of in-vitro antibacterial and anticancer activities of nano-emulsion versus pure plant extract. Drug Dev. Ind. Pharm. 50 (7), 658-670. <https://doi.org/10.1080/03639045.2024.2386001.>

Manoharan, K.P., Yang, D., Hsu, A., Huat, B.T., 2007. Evaluation of Polygonum bistorta for anticancer potential using selected cancer cell lines. Med Chem 3 (2), 121-126. <https://doi.org/10.2174/157340607780059495.>

Neish, A.S., 2009. Microbes in gastrointestinal health and disease. Gastroenterology 136 (1), 65-80. <https://doi.org/10.1053/j.gastro.2008.10.080.>

Niu, C., Li, R.T., Hao, X.S., Qi, X., Wang, F.Z., Fei, H.R., 2025. Scutebarbatine B Exerts Anti-Breast Cancer Activity by Inducing Cell Cycle Arrest and Apoptosis Through Multiple Pathways. Phytother Res 39 (8), 3432-3449. <https://doi.org/10.1002/ptr.70007.>

Nwaeburu, C.C., Abukiwan, A., Zhao, Z., Herr, I., 2017. Quercetin-induced miR-200b-3p regulates the mode of self-renewing divisions in pancreatic cancer. Mol. Cancer 16 (1), 23. <https://doi.org/10.1186/s12943-017-0589-8.>

Oh, H.N., Kwak, A.W., Lee, M.H., Kim, E., Yoon, G., Cho, S.S., Liu, K., Chae, J.I., Shim, J.H., 2021. Targeted inhibition of c-MET by podophyllotoxin promotes caspase-dependent apoptosis and suppresses cell growth in gefitinib-resistant non-small cell lung cancer cells. Phytomedicine 80, 153355. <https://doi.org/10.1016/j.phymed.2020.153355.>

Santosa, M.H., Herzog, R., Voelter, W., 1986. Antitumor Activity of the Hot Water Extract of Lobelia chinensis. Planta Med. (6), 555. <https://doi.org/10.1055/s-2007-969368.>

Shao, H., Chen, J., Li, A., Ma, L., Tang, Y., Chen, H., Chen, Y., Liu, J., 2023. Salvigenin Suppresses Hepatocellular Carcinoma Glycolysis and Chemoresistance Through Inactivating the PI3K/AKT/GSK-3β Pathway. Appl. Biochem. Biotechnol. 195 (8), 5217-5237. <https://doi.org/10.1007/s12010-023-04511-z.>

Shen, J., Lu, X., Du, W., Zhou, J., Qiu, H., Chen, J., Shen, X., Zhong, M., 2016. Lobetyol activate MAPK pathways associated with G1/S cell cycle arrest and apoptosis in MKN45 cells in vitro and in vivo. Biomedicine & pharmacotherapy = Biomedecine & pharmacotherapie 81, 120-127. <https://doi.org/10.1016/j.biopha.2016.03.046.>

Son, I.H., Lee, S.I., Yang, H.D., Moon, H.I., 2007. Bis(4-hydroxybenzyl)sulfide: a sulfur compound inhibitor of histone deacetylase isolated from root extract of Pleuropterus ciliinervis. Molecules 12 (4), 815-820. <https://doi.org/10.3390/12040815.>

Song, H., Sun, C., Li, N., Zhao, Q., He, Y., 2025. Anti-tumor and immunomodulation activity of polysaccharide from Paris polyphylla Smith var. chinensis in S180 tumor-bearing mice. J. Sci. Food Agric. . <https://doi.org/10.1002/jsfa.14415.>

Song, N., Wang, J., Lai, Z., Liang, S., Zou, W., Wang, J., et al. (2022a). Arisaema heterophyllum Blume Monomer Stigmasterol Targets PPARγ and Inhibits the Viability and Tumorigenicity of Lung Adenocarcinoma Cells NCI-H1975. *Evid Based Complement Alternat Med* 2022 5377690. doi:10.1155/2022/5377690

Su, W., Wu, L., Liang, Q., Lin, X., Xu, X., Yu, S., Lin, Y., Zhou, J., Fu, Y., Gao, X., Zhang, B., Li, L., Li, D., Yin, Y., Song, G., 2022. Extraction Optimization, Structural Characterization, and Anti-Hepatoma Activity of Acidic Polysaccharides From Scutellaria barbata D. Don. Front. Pharmacol. 13, 827782. <https://doi.org/10.3389/fphar.2022.827782.>

Sun, J., Cao, Y., Liu, Q., Zhou, Z., Xu, Y., Liu, C., 2024. Chemical Constituents, Anti-Tumor Mechanisms, and Clinical Application: A Comprehensive Review on Scutellaria barbata. Molecules 29 (17), 4134. <https://doi.org/10.3390/molecules29174134.>

Sun, P., Sun, D., Wang, X., 2017. Effects of Scutellaria barbata polysaccharide on the proliferation, apoptosis and EMT of human colon cancer HT29 Cells. Carbohydr. Polym. 167, 90-96. <https://doi.org/10.1016/j.carbpol.2017.03.022.>

Sun, Y., Wang, H., Han, R., Bai, H., Li, M., Wang, J., et al. (2023a). Lignans from the Roots and Rhizomes of Dysosma versipellis and Their Cytotoxic Activities. *Molecules* 28 (7) . doi:10.3390/molecules28072909

Sun, Z., Li, Y., Zhong, R., and Li, R. (2023c). Hypericum sampsonii Hance: a review of its botany, traditional uses, phytochemistry, biological activity, and safety. *Front. Pharmacol.* 14 1247675. doi:10.3389/fphar.2023.1247675

Tan, X.M., Zhou, Y.Q., Zhou, X.L., Xia, X.H., Wei, Y., He, L.L., Tang, H.Z., Yu, L.Y., 2018. Diversity and bioactive potential of culturable fungal endophytes of Dysosma versipellis; a rare medicinal plant endemic to China. Sci. Rep. 8 (1), 5929. <https://doi.org/10.1038/s41598-018-24313-2.>

Timalsina, D., Devkota, H.P., 2021. Eclipta prostrata (L.) L. (Asteraceae): Ethnomedicinal Uses, Chemical Constituents, and Biological Activities. Biomolecules 11 (11), 1738. <https://doi.org/10.3390/biom11111738.>

Wang, B. L., Ge, Z. K., Qiu, J. R., Luan, S. Q., Hao, X. C., and Zhao, Y. H. (2024a). Sedum aizoon L.: a review of its history, traditional uses, nutritional value, botany, phytochemistry, pharmacology, toxicology, and quality control. *Front. Pharmacol.* 15 1349032. doi:10.3389/fphar.2024.1349032

Wang, C., Zhang, M., Peng, J., Zhang, M., Lu, C., Qi, X., et al. (2024b). Combining cisplatin with Pinellia pedatisecta Schott lipid-soluble extract induces tumor immunogenic cell death in cervical cancer. *Phytomedicine* 128 155504. doi:10.1016/j.phymed.2024.155504

Wang, G., Guo, X., Chen, H., Lin, T., Xu, Y., Chen, Q., et al. (2012a). A resveratrol analog, phoyunbene B, induces G2/M cell cycle arrest and apoptosis in HepG2 liver cancer cells. *Biorg. Med. Chem. Lett.* 22 (5), 2114-2118. doi:10.1016/j.bmcl.2011.12.095

Wang, G., Jia, C., Wang, D., Liang, L., Li, L., Tian, G., et al. (2025b). Scutellaria Barbata inhibits epithelial-mesenchymal transformation through PI3K/AKT and MDM2 thus inhibiting the proliferation, migration and promoting apoptosis of Cervical Cancer cells. *PLoS. ONE* 20 (4), e0321556. doi:10.1371/journal.pone.0321556

Wang, M., Wang, S., Hu, W., Wang, Z., Yang, B., and Kuang, H. (2022b). Asparagus cochinchinensis: A review of its botany, traditional uses, phytochemistry, pharmacology, and applications. *Front. Pharmacol.* 13 1068858. doi:10.3389/fphar.2022.1068858

Wang, X. P., Li, X. H., Lei, J. J., Xiao, Y. W., Chi, Y., Sun, Q., et al. (2025f). Polyprenylated acylphloroglucinols from Hypericum sampsonii with cytotoxicity against pancreatic carcinomas. *J. Asian Nat. Prod. Res.* 27 (2), 136-142. doi:10.1080/10286020.2024.2380744

Wang, X., Sahibzada, K. I., Du, R., Lei, Y., Wei, S., Li, N., et al. (2024f). Rhein Inhibits Cell Development and Aflatoxin Biosynthesis via Energy Supply Disruption and ROS Accumulation in Aspergillus flavus. *Toxins (Basel)* 16 (7) . doi:10.3390/toxins16070285

Wang, Y. W., Wang, S. J., Zhou, Y. N., Pan, S. H., and Sun, B. (2012c). Escin augments the efficacy of gemcitabine through down-regulation of nuclear factor-κB and nuclear factor-κB-regulated gene products in pancreatic cancer both in vitro and in vivo. *J. Cancer Res. Clin. Oncol.* 138 (5), 785-797. doi:10.1007/s00432-012-1152-z

Weng, L.L., Xiang, J.F., Lin, J.B., Yi, S.H., Yang, L.T., Li, Y.S., Zeng, H.T., Lin, S.M., Xin, D.W., Zhao, H.L., Qiu, S.Q., Chen, T., Zhang, M.G., 2014. Asparagus polysaccharide and gum with hepatic artery embolization induces tumor growth and inhibits angiogenesis in an orthotopic hepatocellular carcinoma model. Asian Pacific journal of cancer prevention : APJCP 15 (24), 10949-10955. <https://doi.org/10.7314/apjcp.2014.15.24.10949.>

Wu, H., Lai, W., Wang, Q., Zhou, Q., Zhang, R., and Zhao, Y. (2024b). Gypenoside induces apoptosis by inhibiting the PI3K/AKT/mTOR pathway and enhances T-cell antitumor immunity by inhibiting PD-L1 in gastric cancer. *Front. Pharmacol.* 15 1243353. doi:10.3389/fphar.2024.1243353

Wu, J.J., Cheng, K.W., Zuo, X.F., Wang, M.F., Li, P., Zhang, L.Y., Wang, H., Ye, W.C., 2010. Steroidal saponins and ecdysterone from Asparagus filicinus and their cytotoxic activities. Steroids 75 (10), 734-739. <https://doi.org/10.1016/j.steroids.2010.05.002.>

Xi, P., Niu, Y., Zhang, Y., Li, W., Gao, F., Gu, W., Kui, F., Liu, Z., Lu, L., Du, G., 2022. The mechanism of dioscin preventing lung cancer based on network pharmacology and experimental validation. J. Ethnopharmacol. 292, 115138. <https://doi.org/10.1016/j.jep.2022.115138.>

Xiang, M., Su, H., Hong, Z., Yang, T., Shu, G., 2015. Chemical composition of total flavonoids from Polygonum amplexicaule and their pro-apoptotic effect on hepatocellular carcinoma cells: Potential roles of suppressing STAT3 signaling. Food Chem. Toxicol. 80, 62-71. <https://doi.org/10.1016/j.fct.2015.02.020.>

Xiang, M., Su, H., Shu, G., Wan, D., He, F., Loaec, M., Ding, Y., Li, J., Dovat, S., Yang, G., Song, C., 2016. Amplexicaule A exerts anti-tumor effects by inducing apoptosis in human breast cancer. Oncotarget 7 (14), 18521-18530. <https://doi.org/10.18632/oncotarget.7848.>

Xiao, J., Gao, M., Sun, Z., Diao, Q., Wang, P., Gao, F., 2020. Recent advances of podophyllotoxin/epipodophyllotoxin hybrids in anticancer activity, mode of action, and structure-activity relationship: An update (2010-2020). Eur. J. Med. Chem. 208, 112830. <https://doi.org/10.1016/j.ejmech.2020.112830.>

Xiao, M.Y., Pei, W.J., Li, S., Li, F.F., Xie, P., Luo, H.T., Hyun Yoo, H., Piao, X.L., 2024. Gypenoside L inhibits hepatocellular carcinoma by targeting the SREBP2-HMGCS1 axis and enhancing immune response. Bioorg. Chem. 150, 107539. <https://doi.org/10.1016/j.bioorg.2024.107539.>

Xie, P., Luo, H.T., Pei, W.J., Xiao, M.Y., Li, F.F., Gu, Y.L., Piao, X.L., 2024. Saponins derived from Gynostemma pentaphyllum regulate triglyceride and cholesterol metabolism and the mechanisms: A review. J. Ethnopharmacol. 319 (Pt 1), 117186. <https://doi.org/10.1016/j.jep.2023.117186.>

Xin, W.B., Man, X.H., Zheng, C.J., Jia, M., Jiang, Y.P., Zhao, X.X., Jin, G.L., Mao, Z.J., Huang, H.Q., Qin, L.P., 2012. Prenylated phloroglucinol derivatives from Hypericum sampsonii. Fitoterapia 83 (8), 1540-1547. <https://doi.org/10.1016/j.fitote.2012.08.022.>

Xu, T., Wang, Z., Lei, T., Lv, C., Wang, J., Lu, J., 2015. New flavonoid glycosides from Sedum aizoon L. Fitoterapia 101, 125-132. <https://doi.org/10.1016/j.fitote.2014.12.014.>

Xu, X., Gao, X., Jin, L., Bhadury, P.S., Yuan, K., Hu, D., Song, B., Yang, S., 2011. Antiproliferation and cell apoptosis inducing bioactivities of constituents from Dysosma versipellis in PC3 and Bcap-37 cell lines. Cell Div. 6 (1), 14. <https://doi.org/10.1186/1747-1028-6-14.>

Xue, S., Geng, A., Lian, T., Liu, Y., 2022. Scutellaria barbata D. Don inhibits cervical cancer cell proliferation, migration, and invasion via miR-195-5p/LOXL2 axis. Toxicol Res (Camb) 11 (5), 804-811. <https://doi.org/10.1093/toxres/tfac058>

Yang, A.Y., Liu, H.L., Yang, Y.F., 2022. Study on the mechanism of action of Scutellaria barbata on hepatocellular carcinoma based on network pharmacology and bioinformatics. Front. Pharmacol. 13, 1072547. <https://doi.org/10.3389/fphar.2022.1072547.>

Yang, L., Liu, Y. N., Gu, Y., and Guo, Q. (2023b). Deltonin enhances gastric carcinoma cell apoptosis and chemosensitivity to cisplatin via inhibiting PI3K/AKT/mTOR and MAPK signaling. *World. J. Gastrointest. Oncol.* 15 (10), 1739-1755. doi:10.4251/wjgo.v15.i10.1739

Yang, P. W., Ma, S. P., Chen, X. J., Yang, F. M., Wang, S. M., Wang, Q., et al. (2025a). Luteolin suppresses cell migration and invasion via targeting miR-6809-5p/FLOT1/FAK and eliciting EMT in hepatocellular carcinoma. *Transl. Oncol.* 61 102511. doi:10.1016/j.tranon.2025.102511

Yang, S., Shen, T., Zhao, L., Li, C., Zhang, Y., Lou, H., Ren, D., 2014. Chemical constituents of Lobelia chinensis. Fitoterapia 93, 168-174. <https://doi.org/10.1016/j.fitote.2014.01.007.>

Yang, Z., Liu, X., Wang, K., Cao, X., Wu, S., 2013. Novel linear and step-gradient counter-current chromatography for bio-guided isolation and purification of cytotoxic podophyllotoxins from Dysosma versipellis (Hance). J. Sep. Sci. 36 (6), 1022-1028. <https://doi.org/10.1002/jssc.201201038.>

Ying-Ying, Y., Xin-Huan, W., Ying-Nan, L., Ling-Yan, G., Chang-Zheng, Z., 2020. [Research progress on chemical constituents and pharmacological effects of Sedum sarmentosum]. Zhongguo Zhong yao za zhi = Zhongguo zhongyao zazhi = China journal of Chinese materia medica 45 (18), 4341-4348. <https://doi.org/10.19540/j.cnki.cjcmm.20200623.601.>

Yu, L., Lei, L., Cong-kui, T., Da-zhai, Z., 2019. Research progress of studies on chemical constituents and biologic activities of Anemone species. China Journal of Chinese Materia Medica 44 (05), 912-919. <https://doi.org/10.19540/j.cnki.cjcmm.20181226.011.>

Yulan, C., Ya, T.U., Liming, B., Meirong, B., 2025. Research Progress on the Chemical Components and Pharmacological Effects and Quality Marker Prediction of Bistortae Rhizoma. Information on Traditional Chinese Medicine 42 (01), 76-83. <https://doi.org/10.19656/j.cnki.1002-2406.20250113.>

YUlan, L., Yi, S., Chunxing, H.U., Mei, L., Min, Z., Jinshuang, X.U., Huifang, C., 2021. Summary of the Pharmacological Activities of Miao Medicine Polygonum capitatum. Journal of Guizhou University of Traditional Chinese Medicine 43 (01), 81-84. <https://doi.org/10.16588/j.cnki.issn2096-8426.2021.01.021.>

Yun, H.M., Kwon, Y.J., Kim, E., Chung, H.J., Park, K.R., 2023. Machilin D Promotes Apoptosis and Autophagy, and Inhibits Necroptosis in Human Oral Squamous Cell Carcinoma Cells. Int. J. Mol. Sci. 24 (5) . <https://doi.org/10.3390/ijms24054576.>

Yun, H.M., Nhiem, N.X., Park, S., Park, K.R., 2025. Saucerneol Inhibits the Growth, Migration, and Invasion of Osteosarcoma Cells In Vitro and Prevents Metastasis-Associated Osteolysis Ex Vivo. Mol. Nutr. Food Res. , e70187. <https://doi.org/10.1002/mnfr.70187.>

Yun, J.H., Lee, S.B., Kang, K., Lee, E.H., Lee, H.J., Jung, S.H., Nho, C.W., 2013. Bifunctional chemopreventive effects of Adenocaulon himalaicum through induction of detoxification enzymes and apoptosis. J. Med. Food 16 (8), 701-710. <https://doi.org/10.1089/jmf.2012.2625>

Zeng, J.Z., Sun, D.F., Wang, L., Cao, X., Qi, J.B., Yang, T., Hu, C.Q., Liu, W., Zhang, X.K., 2006. Hypericum sampsonii induces apoptosis and nuclear export of retinoid X receptor-alpha. Carcinogenesis 27 (10), 1991-2000. <https://doi.org/10.1093/carcin/bgl046.>

Zhang, Q.Y., Jiang, M., Zhao, C.Q., Yu, M., Zhang, H., Ding, Y.J., Zhai, Y.G., 2005. Apoptosis induced by one new podophyllotoxin glucoside in human carcinoma cells. Toxicology 212 (1), 46-53. <https://doi.org/10.1016/j.tox.2005.04.006.>

Zhang, W., Mi, S., He, X., Cui, J., Zhi, K., and Zhang, J. (2024c). Advancements in Non-Addictive Analgesic Diterpenoid Alkaloid Lappaconitine: A Review. *Int. J. Mol. Sci.* 25 (15) . doi:10.3390/ijms25158255

Zhang, X., Bi, C., Chen, Q., Xu, H., Shi, H., and Li, X. (2021e). Structure elucidation of arabinogalactoglucan isolated from Sedum sarmentosum Bunge and its inhibition on hepatocellular carcinoma cells in vitro. *Int. J. Biol. Macromol* 180 152-160. doi:10.1016/j.ijbiomac.2021.03.051

Zhang, X., Rakesh, K. P., Shantharam, C. S., Manukumar, H. M., Asiri, A. M., Marwani, H. M., et al. (2018b). Podophyllotoxin derivatives as an excellent anticancer aspirant for future chemotherapy: A key current imminent needs. *Biorg. Med. Chem.* 26 (2), 340-355. doi:10.1016/j.bmc.2017.11.026

Zhang, Y., Ji, P., Xiao, X., Wang, J., Wan, Z., Cao, H., et al. (2024d). Network pharmacology, molecular docking, and molecular dynamics simulations shed light on the mechanism behind Gynostemma pentaphyllum's efficacy against osteosarcoma. *Medicine* 103 (35), e39454. doi:10.1097/MD.0000000000039454

Zhao, Y.Z., Zhang, Y.Y., Han, H., Fan, R.P., Hu, Y., Zhong, L., Kou, J.P., Yu, B.Y., 2018. Advances in the antitumor activities and mechanisms of action of steroidal saponins. Chin J Nat Med 16 (10), 732-748. <https://doi.org/10.1016/S1875-5364(18)30113-4.>

Zhao, Z., Ruan, J., Jin, J., Zou, J., Zhou, D., Fang, W., Zeng, F., 2006. Flavan-4-ol Glycosides from the Rhizomes of Abacopteris penangiana. J. Nat. Prod. 69 (2), 265-268. <https://doi.org/10.1021/np050191p.>

Zhen, X., Choi, H.S., Kim, J.H., Kim, S.L., Liu, R., Yun, B.S., Lee, D.S., 2020. Machilin D, a Lignin Derived from Saururus chinensis, Suppresses Breast Cancer Stem Cells and Inhibits NF-κB Signaling. Biomolecules 10 (2), 245. <https://doi.org/10.3390/biom10020245.>

Zou, Y.X., Mu, Z.Q., Wang, J., Tian, S., Li, Y., Liu, Y., 2022. Wedelolactone, a Component from Eclipta prostrata (L.) L., Inhibits the Proliferation and Migration of Head and Neck Squamous Cancer Cells through the AhR Pathway. Curr. Pharm. Biotechnol. 23 (15), 1883-1892. <https://doi.org/10.2174/1389201023666220307110554.>
